# An AMPK phosphoregulated RhoGEF feedback loop tunes cortical flow–driven amoeboid migration in vivo

**DOI:** 10.1126/sciadv.abo0323

**Published:** 2022-09-14

**Authors:** Benjamin Lin, Jonathan Luo, Ruth Lehmann

**Affiliations:** ^1^ Skirball Institute and Department of Cell Biology, NYU Grossman School of Medicine, New York, NY 10016, USA.; ^2^ Whitehead Institute for Biomedical Research, Cambridge, MA 02142, USA.; ^3^ Department of Biology, Massachusetts Institute of Technology, Cambridge, MA 02139, USA.

## Abstract

Development, morphogenesis, immune system function, and cancer metastasis rely on the ability of cells to move through diverse tissues. To dissect migratory cell behavior in vivo, we developed cell type–specific imaging and perturbation techniques for *Drosophila* primordial germ cells (PGCs). We find that PGCs use global, retrograde cortical actin flows for orientation and propulsion during guided developmental homing. PGCs use RhoGEF2, a RhoA-specific RGS-RhoGEF, as a dose-dependent regulator of cortical flow through a feedback loop requiring its conserved PDZ and PH domains for membrane anchoring and local RhoA activation. This feedback loop is regulated for directional migration by RhoGEF2 availability and requires AMPK rather than canonical Gα_12/13_ signaling. AMPK multisite phosphorylation of RhoGEF2 near a conserved EB1 microtubule-binding SxIP motif releases RhoGEF2 from microtubule-dependent inhibition. Thus, we establish the mechanism by which global cortical flow and polarized RhoA activation can be dynamically adapted during natural cell navigation in a changing environment.

## Teaser

Primordial germ cells liberate RhoGEF from microtubule inhibition to tune and orient cortical actin flow for directed migration.

## INTRODUCTION

A shared mechanism among most migrating cells is the coupling of retrograde actin flow to the environment to generate forward motion. Retrograde actin flow is generally confined to the leading lamellipodia and lamellum in slowly migrating, adhesion-dependent mesenchymal cells (*1*), while in a subset of rapidly moving amoeboid cells which do not use protrusions, cortical actin can flow across the entire cell cortex. This protrusion-independent, global cortical flow–driven amoeboid migration mode, referred to hereafter as global cortical flow migration, has many manifestations and can be categorized by the absence (*2*–*4*) or presence of a leading bleb that is stable (*5*–*8*) or unstable (*9*). What is common among global cortical flow migrating cells is a pronounced actomyosin contractility at the rear and a relative independence from adhesion as compared to mesenchymal cells. This migration mode is widespread and has been observed in a variety of cell types in three-dimensional (3D) environments and/or under confinement, including breast cancer cells (*2*), zebrafish progenitor cells (*6*, *10*), melanoma cells (*7*), carcinosarcoma cells (*9*), and various mesenchymal, immune, and epithelial cell lines (*4*, *5*, *11*). Current evidence for global cortical flow migration in vivo has been complicated because definitive observations, such as direct visualization of retrograde cortical flow, required ectopic induction of contractility via expression of constitutively active RhoA or wounding (*6*, *12*). Despite observation in many cell types, its morphological hallmarks in migrating cancer cells in live mice (*8*), and its rapid induction by purely environmental factors (*5*), the molecular underpinnings and regulation of global cortical flow migration remain poorly understood.

An intriguing question is how global cortical flows are organized and maintained in migrating cells on a minute to hour time scale. Cortical actin flows are drawn toward regions of high actomyosin contractility (*13*) and are thought to be stabilized by the advection of front-back polarity factors, such as myosin II (*5*, *6*, *14*, *15*), as well as F-actin polymerization at the front and depolymerization at the rear (*2*, *5*, *6*, *16*). Upstream regulation of cortical flow, however, remains elusive but likely depends on the conserved small Rho guanosine triphosphatase (GTPase) RhoA. A lysophosphatidic acid (LPA)→RhoA axis was required for its induction (*6*), and pharmacological inhibition of its canonical downstream targets Dia (*2*) and ROCK (*2*, *5*, *6*) perturbs migration. Rho guanine nucleotide exchange factors (RhoGEFs) and Rho GTPase-activating proteins promote and repress RhoA activity, respectively, with the regulator of G protein signaling (RGS) family of RhoGEFs serving as a well-described link between G protein–coupled receptor (GPCR)–Gα_12/13_ signaling and RhoA (*17*). PDZ-RhoGEF, a prototypical RGS-RhoGEF, activates RhoA at the rear of migrating neutrophils (*18*), but unlike other RGS-RhoGEFs, its exchange activity is not enhanced by Gα_12/13_ binding (*19*), suggesting alternative forms of regulation. Current studies suggest that external cues, such as confinement, induce but do not orient global cortical flow migration as a means to escape crowded environments (*5*, *6*, *10*, *11*).

**Fig. 1.**
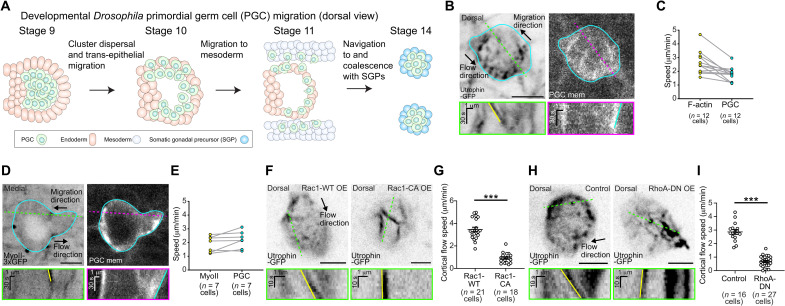
PGC cortical flows are necessary for migration in vivo. (**A**) Schematic of milestones in PGC migration during embryogenesis. (**B** and **D**) Two-photon time-lapse imaging of the dorsal plane of a representative PGC expressing utrophin-GFP (green fluorescent protein) (B) or medial plane of a representative PGC expressing myosin II–3xGFP (D) while coexpressing tdKatushka2-CAAX (PGC membrane marker). Cyan outline traces the boundary of the PGC as defined in the membrane image. Opposing flow and migration directions are marked with black arrows. Green and magenta dashed lines indicate where kymographs below the respective images were taken. The yellow line in the kymograph indicates the retrograde flow of an actin cluster (B) or myosin II foci (D), while the cyan line tracks the rear of the cell. (**C** and **E**) Quantification of actin flow versus cell speeds (C) or myosin II flow versus cell speeds (E). (**F** and **H**) Two-photon time-lapse imaging of the dorsal plane of a representative PGC expressing utrophin-GFP and either (F) Rac1-WT (wild type) (left) or Rac1-CA (constitutively active) (right). Utrophin-GFP is expressed by itself in the control (left) or with RhoA-DN (dominant-negative) in (H). Flow direction is marked with a black arrow. Green dashed lines indicate where kymographs below the respective images were taken. The yellow line in the kymograph indicates retrograde flow in control cells, which is disrupted by the indicated perturbations. All scale bars, 5 μm. (**G** and **I**) Quantification of mean cortical flow speed under the specified conditions. Error bars are SEM. Statistical comparisons are pairwise from a Mann-Whitney test in (G) and (I). ***P < 0.001.

Primordial germ cells (PGCs) in many species are tasked with migrating across a complex, crowded cellular landscape that continuously changes as development proceeds (Fig. 1A) (*20*, *21*). Hence, they require a flexible migration strategy that enables rapid movement through different cells expressing divergent cell adhesion molecules. One such strategy has been outlined in zebrafish PGCs, where F-actin accumulation templates the site of bleb formation and the subsequent local retrograde cortical actin flow following bleb expansion generates the necessary friction for forward movement (*12*, *22*, *23*). Different strategies are likely to exist for other PGCs, including in *Drosophila*, where, in contrast to zebrafish PGCs, the activity of the small Rho GTPase Rac1 is not polarized (*24*) and expression of a dominant-negative Rac1 does not impair trans-epithelial PGC migration (*25*). In this study, we establish that *Drosophila* PGCs use global cortical flow migration during guided developmental homing in vivo and reveal an upstream cortical flow tuning pathway necessary for accurate guidance.

## RESULTS

### PGCs use global cortical flow migration to navigate in vivo

*Drosophila* PGCs maintain a spherical morphology and intact cortex while rapidly migrating through diverse adhesive cellular substrates during development (*24*). This migratory morphology is characteristic of a subtype of amoeboid migration whereby rear-end contractility directs global cortical actin flow used by epithelial cells (*4*) and cancer cells (*2*) in 3D matrix, zebrafish progenitor cells upon wounding in vivo (*6*), and a variety of cell lines upon confinement and low adhesion (*5*). To determine whether PGCs endogenously use this type of migration in vivo, we optimized imaging parameters to visualize F-actin dynamics along the cortex of PGCs. We overexpressed the F-actin–binding protein utrophin-GFP (green fluorescent protein) in embryos and identified PGCs by a PGC-specific membrane marker (tdKatushka2-CAAX). Viewed from the dorsal surface of embryo, we followed directed migration toward the lateral mesoderm with a fine temporal resolution (2.5 s every frame) and observed a clear retrograde flow of cortical actin clusters commensurate with PGC movement in the opposite direction (Fig. 1B and movie S1). Cortical actin flow speeds were consistently faster than PGC migration speeds (Fig. 1C). Cortical actin flows can advect actin-binding proteins, such as myosin II, toward the cell rear to establish and maintain front-rear migratory polarity (*5*, *6*, *14*, *15*). To determine whether myosin II underwent a similar retrograde flow as actin during PGC migration, we imaged PGCs expressing a myosin II–3xGFP transgene under endogenous regulation. Although we were unable to resolve myosin II clusters on the dorsal plane of PGCs, we observed the retrograde flow of myosin II in the medial cortex near the cell rear during migration (Fig. 1D, fig. S1A, and movie S2). As opposed to actin flow, myosin II flow speed was on par with migration speed (Fig. 1E), suggesting that reduced cortical flow speeds near the cell rear correlate with propulsion, as observed in other migrating cells (*5*, *6*).

To determine whether retrograde cortical flows are required for PGC migration, we characterized cortical organization and flow under known small Rho GTPase perturbations, which had been shown to impair PGC migration in fixed samples (*25*). Overexpression of constitutively active Rac1 (Rac1-G12V) under live observation generated thick, immobile filamentous cortical actin structures in most PGCs (15 of 18 PGCs) at the expense of typical, motile cortical clusters present when overexpressing Rac1-WT (wild type) (Fig. 1, F and G, and movie S3). Overexpression of constitutively active RhoA (RhoA-G14V) similarly abolished motile cortical clusters found in control PGCs but, in contrast, engendered a bright, homogeneous cortex qualitatively devoid of motion (41 of 41 PGCs) (Fig. 1H, fig. S1B, and movie S4). A subset of these PGCs had static ring structures (11 of 41 PGCs) (fig. S1C). Last, overexpression of dominant-negative RhoA (RhoA-T19N) also perturbed cortical architecture and generated larger, stationary F-actin aggregates with significantly reduced cortical flow speed (Fig. 1, H and I, and movie S4). In more than half of these PGCs (16 of 28), cortical filaments were discernible (Fig. 1H), suggesting that cortical actin density had been reduced. PGC cortical flows are thus exquisitely sensitive to alterations in cortical organization. We conclude that WT PGCs use global cortical flow migration during external cue–guided developmental migration in vivo.

### Extracted PGCs maintain cortical flows dependent on actomyosin contractility and formin-mediated actin polymerization

**Fig. 2.**
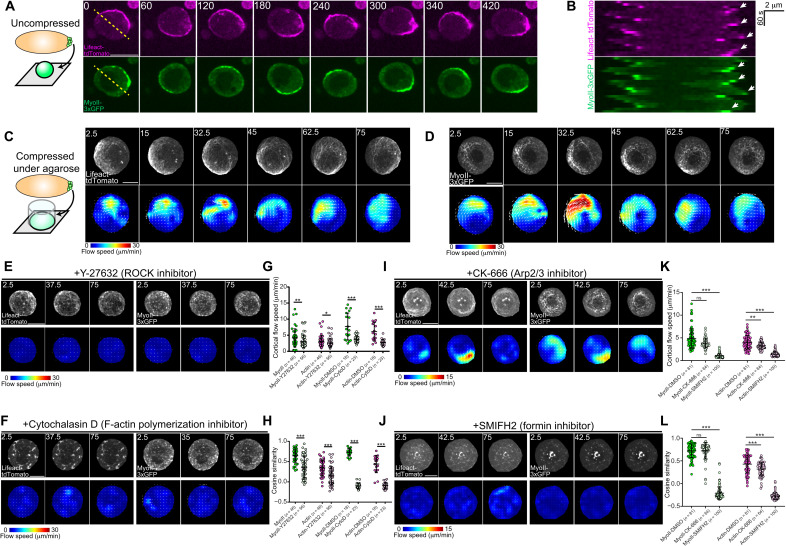
Molecular requirements for PGC cortical flow. (**A**) Time-lapse imaging of an extracted PGC on a coverslip in medium without serum expressing lifeact-tdTomato and myosin II–3xGFP. The yellow dashed line indicates where the kymograph in (**B**) was taken. (B) Kymograph from (A), with white arrows indicating periodic enriched F-actin and myosin II due to circular flow. (**C** and **D**) Top: Time-lapse imaging of a representative extracted PGC expressing lifeact-tdTomato (C) and myosin II–3xGFP (D) under agarose. Bottom: PIV flow analysis between consecutive images. Flow speed is color-coded with the indicated color bar. White arrows are flow vectors scaled to flow magnitude. (**E**, **F**, **I**, and **J**) Top: Time-lapse imaging of a representative extracted PGC expressing lifeact-tdTomato (left set) and myosin II–3xGFP (right set) under agarose with Y-27632 (E), cytochalasin D (F), CK-666 (I), or SMIFH2 (J). Bottom: PIV flow analysis between consecutive images. Flow speed is color-coded with the indicated color bar. White arrows are flow vectors scaled to flow magnitude. (**G** and **K**) Quantification of mean cortical flow speed of myosin II and actin under the indicated treatment conditions. *n* = number of cells analyzed under each condition. (**H** and **L**) Quantification of mean cosine similarity of myosin II and actin vectors over consecutive time points under the indicated treatment conditions. Error bars are SD. Time is in seconds in all images. All scale bars, 10 μm. Statistical comparisons are pairwise from a Mann-Whitney test in (G), (H), (K), and (L). *P < 0.05, **P < 0.01, and ***P < 0.001. ns, not significant.

The induction of global cortical flow migration in most cells requires high levels of contractility, induced by confinement and/or contractile stimuli, such as LPA and serum (*2*, *4*–*7*). We asked whether PGC cortical flows require equivalent cues from the embryonic environment and developed methods to rapidly extract PGCs from stage 5 embryos to observe PGC dynamics in culture (Fig. 2, A and B, and fig. S2; see Materials and Methods). Extracted PGCs expressing lifeact-tdTomato and myosin II–3xGFP plated onto uncoated glass in serum-free medium maintained their spherical morphology and exhibited four classes of cell behaviors: (i) periodic, circular flows of cortical actin and myosin II (Fig. 2, A and B, and movie S5) with a mean period of 86 ± 21.7 s (SD) (41%, 78 of 188 cells), (ii) stochastic accumulations of myosin II along the cortex (29%, 54 of 188 cells) (fig. S2, A and B), (iii) blebbing (19%, 35 of 188 cells) (fig. S2, C and D), and (iv) inactive, where myosin II remained cytoplasmic (11%, 21 of 188 cells) (fig. S2, E and F). Thus, as opposed to other cells that require an ectopically induced increase in contractility and/or confined environment to observe global cortical flows (*2*, *4*–*7*), most of the extracted PGCs exhibit inherent continuous cell-scale cortical flow. We propose that embryonic guidance cues act by harnessing and directing these autonomous flows for developmental PGC navigation in vivo.

Placing PGCs under agarose substantially increased the surface area of the ventral cortex (nearest to glass), allowing us to image the flow of actin and myosin II cortical networks under high spatiotemporal resolution (Fig. 2, C and D, and movie S6). Particle image velocimetry (PIV) analysis indicated that myosin II foci traveled with the cortical actin network in sweeping, circular flows across the cell, mirroring our observations in uncompressed PGCs (Fig. 2, A and B). Cortical actin and myosin II flow speeds reached upward of 30 μm/min (Fig. 2, C and D), an order of magnitude greater than the mean flow speeds that we measured in vivo (Fig. 1, C and E). These higher speeds are likely due to an uncoupling between actin flow and the environment, as PGCs remained stationary under these conditions, and may also arise from increased contractility under confinement, as observed in a variety of other cells (*5*, *10*).

Our ability to extract PGCs for in vitro experimentation allowed us to compare and extend the molecular requirements we identified for cortical flow in vivo (Fig. 1, F to I, and fig. S3). Cortical flows have previously been shown to be dependent upon cortical contractility and actin polymerization (*2*, *5*, *6*), in line with the perturbed flow that we observed when manipulating RhoA activity in PGCs (Fig. 1, H and I, and fig. S1, B and C). We assessed the contribution of each process individually with specific inhibitors (Fig. 2, E to L). Reducing actomyosin contractility via inhibition of the canonical upstream activator of myosin II, ROCK, with a high dose of Y-27632 (100 μM) led to a significant reduction in mean cortical actin and myosin II flow speeds, a shift in the cumulative distribution of all flow speeds, and a decrease in flow coordination (assessed by cosine similarity between vectors at the same *XY* coordinate over consecutive time points) in the bulk population (Fig. 2, E, G, and H; fig. S3, A and B; and movie S6). However, we did observe cells that appeared to have WT flow speeds and organized flows (Fig. 2, E and G), suggesting that PGCs may use other kinases to activate myosin II and/or use actin polymerization to drive flow. In contrast to the heterogeneity that we observed with ROCK inhibition, inhibiting actin polymerization with cytochalasin D fractured the actin cortex and led to diffusive myosin II movements, essentially stopping cortical flow in all cells as compared to control (Fig. 2, F to H; fig. S3, C, D, and I; and movie S7). Together, these results suggest that extracted PGCs maintain intrinsic cortical actin and myosin II flows observed in vivo, which are dependent upon contractility and actin polymerization.

To distinguish the actin nucleators required for cortical flow in PGCs, we inhibited two canonical actin nucleators involved in cell motility, the Arp2/3 complex and formins (Fig. 2, I to L, and movie S8). Arp2/3 complex inhibition with CK-666 at concentrations (100 μM) previously shown to inhibit dendritic actin networks in insect cell lamellipodia (*26*) did not generate a visually distinct change in cortical flow but led to a ~20% reduction in actin cortical flow speed and decreased organization without significantly affecting myosin II flow (Fig. 2, I, K, and L, and fig. S3, E, F, and J). In contrast, the formin inhibitor SMIFH2 disassembled much of the actin cortex, mimicking the effects of bulk actin polymerization inhibition with cytochalasin D (Fig. 2, J to L; fig. S3, G, H, and J; and movie S8). Both actin and myosin II flow speeds significantly decreased (actin flow speed reduced by ~65% and myosin II flow speed reduced by ~79%), and coherence was lost (Fig. 2, K and L). We conclude that global cortical flows in PGCs are largely dependent on formin-mediated linear actin polymerization rather than Arp2/3 complex–mediated branched actin polymerization, with the latter assertion requiring further genetic evidence.

### RhoGEF2 is enriched at the rear of PGCs throughout developmental migration

Given that PGCs are guided by external cues during developmental migration (*20*), there must be an upstream module to control the inherent cortical flows in PGCs. In migrating amoeboid cells, actin flow speed can be tuned by GPCR signaling but the downstream pathways connecting the two remain unclear (*27*). One pathway likely involved is the conserved contractility pathway downstream of the small Rho GTPase, RhoA, whereby guanosine triphosphate (GTP)–bound, active RhoA binds and relieves autoinhibition of ROCK, which then phosphorylates and activates myosin II. Cortical flow is drawn toward regions of high actomyosin contractility (*13*), and we have previously shown that RhoA is active at the rear of migrating PGCs (*24*). We thus hypothesized that regulation of a RhoGEF may modulate cortical flow.

**Fig. 3.**
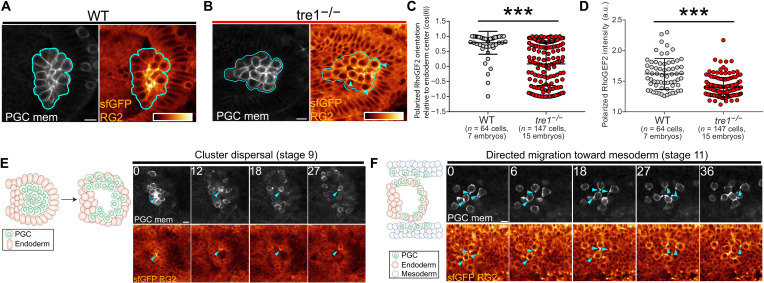
RhoGEF2 is posteriorly enriched throughout developmental migration. (**A** and **B**) Representative two-photon image of the central plane of a WT (A) or tre1^−/−^ (B) PGC cluster expressing sfGFP-RhoGEF2 (RG2) and tdKatushka2-CAAX (PGC membrane marker). Cyan outlines the PGC cluster, while cyan arrows indicate regions of polarized RhoGEF2. sfGFP-RhoGEF2 is pseudo-colored with the indicated color bar and scaled to the same intensity range. (**C** and **D**) Quantification of polarized RhoGEF2 orientation relative to the center of the cluster (C) or polarized RhoGEF2 intensity (D). Error bars are SD. a.u., arbitrary units. (**E** and **F**) Two-photon time-lapse imaging of representative PGCs expressing sfGFP-RhoGEF2 and tdKatushka2-CAAX (PGC membrane marker) during cluster dispersal (*n* = 7 embryos imaged) (E) and migration toward mesoderm (*n* = 6 embryos imaged) (F). Cyan arrows indicate regions of polarized RhoGEF2 accumulation. sfGFP-RhoGEF2 is pseudo-colored with the color bar in (A). Time is in minutes in all images. All scale bars, 10 μm. Statistical comparisons are pairwise from a Mann-Whitney test in (C) and (D). ***P < 0.001.

To identify candidate RhoGEFs involved in PGC migration, we assessed available GFP-tagged RhoGEFs via live imaging for enrichment at the rear of migrating PGCs (Fig. 3). During stage 9 of embryogenesis, PGCs reside in a cluster and radially align front-rear polarity outward under the guidance of the GPCR, Tre1, thus providing a robust readout for rear-end proteins that enrich in the center of the cluster (Fig. 1A) (*24*). We found that superfolder GFP (sfGFP)–tagged RhoGEF2, a RhoA-specific RhoGEF, driven by a ubiquitous *squash* (*Drosophila* myosin II regulatory light chain) promoter was concentrated at the rear of polarized PGCs (Fig. 3, A and C). This enrichment was not due to overexpression, as an sfGFP-RhoGEF2 fosmid, where RhoGEF2 is driven by native regulation, had a similar enrichment and immunostaining of endogenous RhoGEF2 revealed a similar rear-end polarization in migrating PGCs (fig. S4). In the absence of Tre1, PGCs are unable to separate (*24*, *25*, *28*–*30*) and exhibit extensive intracluster motility and cell turning. As a result, rear enriched proteins, such as myosin II, normally found in the center of the cluster appear randomly distributed in *tre1* mutant PGCs. We similarly observed that polarized RhoGEF2 appeared randomly oriented in *tre1* mutants and it was not as strongly polarized as in WT PGCs (Fig. 3, B to D). This suggests that RhoGEF2 stochastically accumulates on the membrane during unguided motility and can be fixated and further polarized under GPCR guidance. There was a significant genetic interaction between RhoGEF2 and Tre1 in PGC guidance (fig. S5, A and B), suggesting that GPCR signaling can act through RhoGEF2. Two-photon live imaging of migrating PGCs during various stages of development further confirmed that RhoGEF2 was enriched at the rear during all phases of PGC migration (Fig. 3, E and F, and movies S9 and S10).

### RhoGEF2 regulates cortical flow and is necessary for accurate guidance

**Fig. 4.**
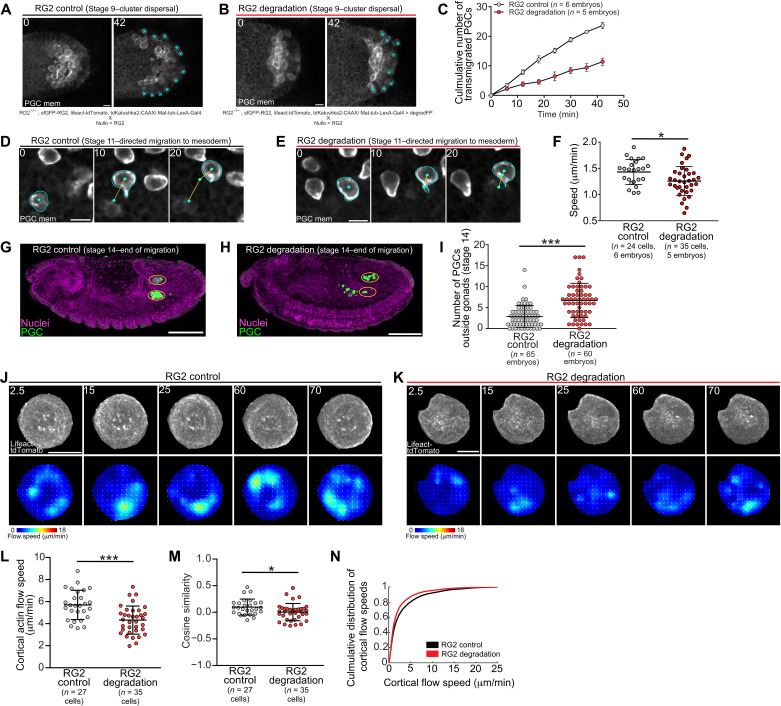
RhoGEF2 regulates cortical flow and is required for accurate guidance. (**A**, **B**, **D**, and **E**) Representative two-photon time-lapse imaging of PGCs expressing tdKatushka2-CAAX (PGC membrane marker) under the indicated conditions during cluster dispersal (A and B) or directed migration toward the mesoderm (D and E). Genotypes are listed below images. Cyan asterisks mark transmigrated PGCs in (A) and (B). Cyan outlines cell membranes, cyan dots track PGC positions, and yellow lines are cell tracks in (D) and (E). Scale bars, 10 μm. Times are in minutes. (**C**, **F**, and **I**) Quantification of the total number of transmigrated PGCs over time (C), mean cell speed (F), and number of mismigrated PGCs (I) under the indicated conditions. Error bars are SEM in (C) and SD in (F) and (I). (**G** and **H**) Representative immunofluorescence images of stage 14 embryos under the indicated conditions. Yellow ovals indicate the gonads. Scale bars, 100 μm. (**J** and **K**) Top: Time-lapse imaging of representative extracted PGCs under agarose with indicated experimental conditions expressing lifeact-tdTomato. Bottom: PIV flow analysis between consecutive images. Flow speed is color-coded with the indicated color bar. White arrows are flow vectors scaled to flow magnitude. Scale bars, 10 μm. Times are in seconds. (**L** and **M**) Quantification of mean cortical actin flow speed (L) and mean cosine similarity between vectors over consecutive time points (M) under the indicated conditions. Error bars are SD. (**N**) Cumulative distributions of flow speeds under the indicated conditions. Statistical comparisons are pairwise from a Mann-Whitney test in (F), (I), (L), and (M). *P < 0.05 and ***P < 0.001.

To assess whether RhoGEF2 is necessary for PGC migration, we used a GFP degradation system, degradFP (*31*), to degrade maternal sfGFP-RhoGEF2 in a genetic background where sfGFP-RhoGEF2 is the sole source of RhoGEF2 in the embryo. We created a PGC-targeted LexA inducible degradFP transgene and drove it with a newly constructed maternal LexA driver (maternal tubulin promoter). As RhoGEF2 is necessary for somatic cellularization (*32*), we rescued any somatic defects via overexpression of an untagged RhoGEF2 using an early soma–specific Gal4 driver, *nullo*-Gal4. The use of orthogonal LexA and Gal4 systems prevented any potential cross-activation between these constructs. Live imaging indicated that RhoGEF2 was significantly depleted (reduced to ~35%) before the dispersal of PGC clusters, allowing us to assess its role in directed migration–dependent cluster dispersal and subsequent steps in developmental migration (Fig. 1A and fig. S5, C and D). RhoGEF2 depletion perturbed the Tre1-dependent outward alignment of front-back polarity in PGC clusters, as shown by the broader distribution of active RhoA [shown by active RhoA biosensor, Anillin-RhoA–binding domain (RBD) fused to tdTomato] in the cluster (fig. S5, E and F), and significantly decreased the rate at which PGCs detached from clusters and transmigrated through the endoderm (Fig. 4, A to C, and movie S11), suggesting defects in generating and orienting motility. Following delayed cluster detachment, we similarly observed that PGC migration speed was reduced during directed migration toward the mesoderm (Fig. 4, D to F, and movie S12). Consequently, more PGCs failed to reach the gonad at stage 14, when developmental migration has concluded (Fig. 4, G to I).

The differences in RhoGEF2 polarity in unguided versus guided migration and its interactions with Tre1 (Fig. 3, A, B, and D, and fig. S5, A, B, E, and F) suggest that RhoGEF2 has dual roles in driving inherent flows and orienting them. Accordingly, the migration defects that we observed after RhoGEF2 depletion could arise from perturbing both processes, as a flatter actomyosin contractility gradient in these PGCs (fig. S5, E and F) would less efficiently drive and organize flow. We directly tested whether RhoGEF2 regulates inherent flows by extracting control and RhoGEF2-depleted PGCs from embryos and imaging cortical actin flow using our agarose assay. Extracted PGCs are no longer under the influence of embryonic cues, similar to *tre1* mutants at stage 9 of embryogenesis, allowing us to decouple specific contributions of RhoGEF2 to generating constitutive flows from a role in guided migration. Cortical flow speeds were significantly slower and more disorganized in RhoGEF2-depleted PGCs, suggesting that RhoGEF2 plays an important role in organizing and driving basal flows (Fig. 4, J to N, and movie S13). In sum, our results suggest that RhoGEF2-dependent cortical flow modulation is necessary for PGC migration in vivo.

### Excess RhoGEF2 activation enhances cortical flow and polarity but impairs guidance

**Fig. 5.**
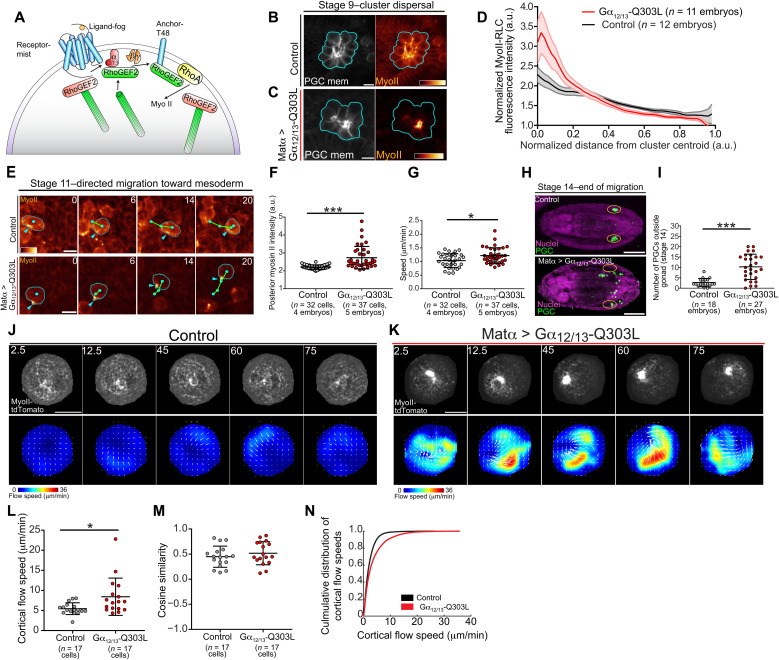
Excess RhoGEF2 activation enhances polarity and speed but impairs homing. (**A**) Canonical RhoGEF2 pathway in gastrulation. (**B** and **C**) Representative two-photon image of a central plane from PGC clusters expressing myosin II–tdTomato and tdKatushka2-CAAX under the indicated conditions. Cyan outlines the perimeter of the cluster, and myosin II–tdTomato is pseudo-colored with the indicated color bar to the same intensity range. Scale bars, 10 μm. (**D**) Quantification of myosin II–tdTomato intensity relative to distance from cluster centroid. Error bars are SEM. (**E**) Representative two-photon time-lapse imaging of PGCs expressing myosin II–tdTomato migrating to the mesoderm under the indicated conditions. Cyan outlines the PGC periphery, cyan dots track PGC positions, cyan arrows highlight polarized myosin II, and green lines are cell tracks. Times are in minutes. Scale bars, 10 μm. (**F**, **G**, and **I**) Quantification of polarized myosin II intensity (F), speed (G), and mismigrated PGCs (I) in the indicated conditions. Error bars are SD. (**H**) Representative immunofluorescence images of stage 14 embryos under the indicated conditions. Yellow ovals mark the gonads. Scale bars, 100 μm. (**J** and **K**) Top: Time-lapse imaging of representative extracted PGCs under agarose with indicated experimental conditions expressing myosin II–tdTomato. Bottom: PIV flow analysis between consecutive images. Flow speed is color-coded with the indicated color bar. White arrows are flow vectors scaled to flow magnitude. Scale bars, 10 μm. Times are in seconds. (**L** to **N**) Quantification of mean cortical actin flow speed (L), mean cosine similarity between vectors over consecutive time points (M), or cumulative flow speed distributions (N) under the indicated conditions. Error bars are SD. Statistical comparisons are pairwise from a Mann-Whitney test in (F), (G), (I), and (L). *P < 0.05 and ***P < 0.001.

Our results suggest that RhoGEF2 activity may control PGC migration by orienting and tuning actin flow speed. Therefore, we asked whether enhanced RhoGEF2 activity would alter cortical actin flow and affect PGC migration. RhoGEF2 is a microtubule plus-end tracking RGS-RhoGEF best known for its role in gastrulation, where it lies downstream of a well-described linear cascade (Fig. 5A). At the top of this pathway, the ligand, Fog, activates a GPCR, Mist, leading to Gα_12/13_ (known as Concertina in *Drosophila*) activation, which then binds and activates RhoGEF2 (*33*–*37*). Gα_12/13_, however, is not required for PGC migration (*25*). Given that RhoGEF2 is the only known target of Gα_12/13_ in *Drosophila*, expression of a constitutively active Gα_12/13_ (Gα_12/13_-Q303L) would selectively activate RhoGEF2 (*38*). Thus, we targeted Gα_12/13_-Q303L translation specifically to PGCs with a hybrid 3′ untranslated region (UTR) driven by maternal Gal4-VP16 expression (*24*) and examined the effects of constitutively active Gα_12/13_ on RhoA activity with live two-photon imaging of a RhoA activity sensor. Contrary to our expectation of global activation, upon examining stage 9 PGC clusters, we observed increased RhoA activation solely in the cluster center, suggesting that global RhoGEF2 activation did not disrupt but rather enhanced front-back polarity (fig. S6). This enhanced posterior RhoA activity led to a concomitant increase in myosin II enrichment in the cluster center, as assessed with a specifically designed transgenic line expressing myosin II–tdTomato and tdKatushka2-CAAX (PGC membrane marker) in PGCs (Fig. 5, B to D). Live imaging of PGC migration toward the mesoderm revealed enhanced myosin II polarity in individual cells and a significant increase in migration speed (Fig. 5, E to G, and movie S14). This enhanced front-back polarity and speed, however, severely disrupted PGC guidance to the gonad (Fig. 5, H and I), suggesting potential defects in orienting or stopping migration.

The enhanced myosin II accumulation that we observed after Gα_12/13_-Q303L overexpression could arise from the acceleration of cortical flow, as faster cortical flows can steepen front-back gradients of actin-binding polarity factors, such as myosin II, as they travel further for a given time period (*14*). We tested this idea by extracting control and Gα_12/13_-Q303L–overexpressing PGCs and imaging cortical myosin II flow. Activating RhoGEF2 led to a significant increase in basal cortical myosin II flow speeds without affecting flow organization, and we identified large myosin II foci in Gα_12/13_-Q303L–overexpressing PGCs (Fig. 5, J to N, and movie S15), in accord with our observations in vivo (Fig. 5, E and F). Our results collectively indicate that levels of RhoGEF2 activity correlate with cortical flow and migration speeds and further suggest that cortical flow speeds must be tuned for accurate homing.

### RhoGEF2 PDZ and PH domains are required for polarity and migration

**Fig. 6.**
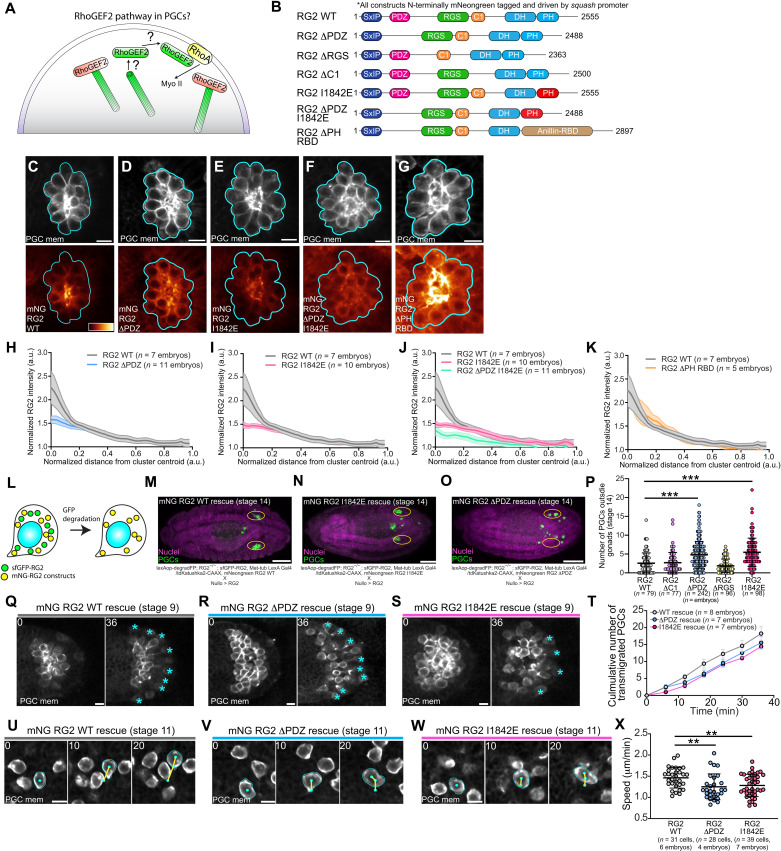
RhoGEF2 PDZ and PH domains are necessary for polarity and migration. (**A**) Unknown RhoGEF2 pathway in PGCs. (**B**) Schematic of RhoGEF2 protein domains and constructs created based on domain truncations and mutations. All constructs are driven by a ubiquitous squash (myosin II regulatory light chain) promoter and are N-terminally tagged with mNeongreen. (**C** to **G**) Representative two-photon image of a central plane from PGC clusters expressing the indicated mNeongreen RhoGEF2 transgene and tdKatushka2-CAAX (PGC membrane marker). mNeongreen-RhoGEF2 transgenes are pseudo-colored with the color bar in (C) and are scaled to the same intensity range. Cyan outlines the perimeter of the cluster. Scale bars, 10 μm. (**H** to **K**) Quantification of the indicated mNeongreen RhoGEF2 transgene intensity relative to distance from cluster centroid versus WT. Error bars are SEM. (**L**) Experimental scheme to assess domain-specific requirements of RhoGEF2. (**M** to **O**) Representative immunofluorescence images of stage 14 embryos under the indicated rescue conditions. Genotypes are stated below. Yellow ovals mark the gonads. Scale bars, 100 μm. (**P**, **T**, and **X**) Quantification of the number of PGCs outside gonads (P), transmigrated PGCs over time (T), or mean speed (X) under indicated rescue conditions. Error bars are SD in (P) and (X) and SEM in (T). (**Q** to **S** and **U** to **W**) Two-photon time-lapse imaging of representative PGC clusters expressing tdKatushka2-CAAX (PGC membrane marker) dispersing (Q to S) or individual PGCs migrating to the mesoderm (U to W) under the indicated rescue conditions. Cyan asterisks mark transmigrated PGCs in (Q) to (S), while cyan outlines cell membranes, cyan dots track nuclei, and yellow lines are cell tracks in (U) to (W). Scale bars, 10 μm. Times are in minutes. Statistical comparisons are pairwise from a Mann-Whitney test in (P) and (X). **P < 0.01 and ***P < 0.001.

To determine how RhoGEF2 is polarized and activated in migrating PGCs, we sought to identify potential regulatory domains and their contacts (Fig. 6A). Like many RhoGEFs, RhoGEF2 is a large multidomain protein and has four notable domains: an N-terminal PDZ domain, an RGS domain responsible for binding Gα_12/13_, a C1 domain, and an invariable tandem Dbl homology (DH) and pleckstrin homology (PH) domain necessary for catalytic activity (Fig. 6B). Previous work has shown that the PDZ domain regulates RhoGEF2 localization through interaction with transmembrane protein anchors, such as T48 during gastrulation (*39*) (Fig. 5A) and Slam during cellularization (*40*). However, these anchors are not expressed in PGCs (*39*, *41*). Moreover, although Gα_12/13_ is the canonical RhoGEF2 activator in *Drosophila* (*36*), we have previously shown that Gα_12/13_ is not required for PGC migration (*25*). Thus, an alternative localization and activation mechanism must exist in PGCs (Fig. 6A).

To identify which domains are necessary for RhoGEF2 function in PGCs, we created a panel of transgenic lines driving mNeongreen-tagged RhoGEF2 (Fig. 6, B to K) lacking the PDZ, RGS, or C1 domain from a ubiquitous *squash* promoter (Fig. 6B). In insect S2 cells, all constructs retained their ability to track microtubule plus-ends and activated myosin II in a similar dose-dependent manner, with the highest activation levels with the ΔRGS construct, suggesting that these truncations did not grossly affect RhoGEF2 function (fig. S7, A to C). The PDZ and RGS domains were, however, essential for viability, while the ΔC1 transgene provided a comparable rescue to WT (Table S1). These differences did not arise from expression levels, as all transgenes were expressed at comparable levels in embryos (fig. S7, D and E). Live imaging of stage 5 embryos confirmed that only the ΔPDZ transgene was not enriched on cellularization furrows (fig. S7, F and G), in line with previous work (*40*). Live imaging of stage 9 PGC clusters expressing these RhoGEF2 transgenes and a PGC-specific membrane marker (tdKatushka2-CAAX) revealed that removal of the PDZ domain reduced but did not completely abolish RhoGEF2 polarization (Fig. 6, C, D, and H, and fig. S7, H to K).

Given that RhoGEF2 ΔPDZ retained some polarization in PGCs, we sought to identify other functional domains in RhoGEF2 that could provide localized RhoGEF2 function. Recent work has shown that the PH domain of most RGS and related RhoGEFs can bind active RhoA-GTP in vitro. The PH domain in these RhoGEFs is located just distal of the catalytic DH domain and provides a localization-based positive feedback mechanism upon activation (*17*). Vesicle-bound, active RhoA potentiates the exchange activity of PDZ-RhoGEF up to 40-fold in vitro, while dampening this feedback by mutating conserved hydrophobic residues in its PH domain critical for active RhoA interaction substantially attenuates RhoA activation upon overexpression in mammalian cells (*17*).

To assess whether this localization-mediated feedback loop exists for RhoGEF2, we first asked whether the RhoGEF2 PH domain could bind RhoA-GTP in an in vitro binding assay. An in vitro translated RhoGEF2 PH domain bound purified glutathione *S*-transferase (GST)–RhoA–guanosine diphosphate (GDP) and GST-RhoA-GTPγS but not GST, suggesting that this interaction was specific for RhoA (fig. S8, A to C). Introducing a point mutation (I1842E) in one of two conserved hydrophobic residues in the PH domain critical for RhoA-GTP binding in other RGS-RhoGEFs decreased both affinities but had a stronger effect on RhoA-GTP binding, suggesting that these residues are also important for RhoGEF2 PH-RhoA interaction. Overexpression of mNeongreen-RhoGEF2 with either PH point mutation (I1842E and F1840A) expectedly reduced myosin II activation in S2 cells at comparable expression levels versus WT but did not affect microtubule plus-end tracking (fig. S8, D to F). This result is consistent with the finding that mutating these residues suppresses the lethality associated with constitutive expression of an optogenetic RhoA activation system using the DH-PH domain from RhoGEF2 (*42*).

To determine whether this feedback was functionally important in vivo, we established *squash* promoter–driven mNeongreen-tagged RhoGEF2 transgenic lines containing these PH domain mutants (Fig. 6B). Both RhoGEF2 PH mutant lines were expressed at similar levels as WT but could not rescue the lethality of RhoGEF2 mutants (fig. S7, D and E, and table S1), suggesting that the RhoGEF2 PH-RhoA interaction is critical for RhoGEF2 function. These PH mutations did not perturb enrichment on cellularization furrows in stage 5 embryos (fig. S8, G and H). In stage 9 PGC clusters, however, both PH mutations reduced RhoGEF2 polarization to a similar extent as the ΔPDZ construct, suggesting that this RhoA binding feedback contributes to RhoGEF2 localization and likely RhoA activation in PGCs (Fig. 6, E and I, and fig. S8, I and J). To assess whether the PDZ and PH domains localize RhoGEF2 in parallel, we generated a double mNeongreen ΔPDZ, I1842E RhoGEF2 transgenic line and observed a further reduction in RhoGEF2 polarity in PGC clusters compared to either domain perturbation alone (Fig. 6, B, F, and J). Collectively, our results suggest that RhoGEF2 is regulated in PGCs through two parallel localization mechanisms: (i) PDZ domain binding to an unknown anchor to enrich it at the cell rear and (ii) PH domain binding to RhoA-GTP to amplify nascent sites of RhoA activity triggered by its basal activity.

If the PH domain acts to enhance RhoGEF2 activity by retaining it near its substrate, a domain with a similar function should be able to compensate for its loss. We tested this idea by generating a transgenic line where the RhoGEF2 PH domain was swapped with the RBD from Anillin (Fig. 6B). Although this transgene could not rescue lethality associated with a RhoGEF2 mutant, it recapitulated WT levels of RhoGEF2 polarity in stage 9 PGC clusters (Fig. 6, G and K), suggesting that RhoA binding is the main function of the PH domain in PGCs.

We next sought to determine whether the same RhoGEF2 domains necessary for polarity were likewise important for PGC migration. Since the mNeongreen ΔPDZ, ΔRGS, and PH domain mutant RhoGEF2 transgenes could not rescue RhoGEF2 mutants (table S1), we could not directly assess if these domains were critical for PGC migration without the confounding presence of the WT protein. To overcome this limitation, we rescued RhoGEF2 null embryos with sfGFP-RhoGEF2 while coexpressing a given mNeongreen RhoGEF2 transgene. We then removed sfGFP-RhoGEF2 with degradFP using the experimental scheme described earlier, allowing us to determine whether the remaining mNeongreen RhoGEF2 protein was sufficient for PGC migration (Fig. 6L). In accord with the polarity phenotypes we observed above, removing the PDZ domain or mutating the PH domain disrupted PGC homing more severely than any other domain perturbation (Fig. 6, M to P). Live imaging further confirmed that these homing defects stemmed from a decreased cluster dispersal rate and slowed migration (Fig. 6, Q to X, and movies S16 and S17).

The PDZ and PH domains could mediate distinct aspects of RhoGEF2-dependent flow generation and/or orientation. We tested this idea by evaluating basal flow in extracted, rescued PGCs (fig. S9). As both domains are required for accurate PGC navigation (Fig. 6, M to P), unperturbed basal flow would suggest that a given domain is required to direct flow by localizing RhoGEF2 under embryonic guidance cues. PGCs lacking the PDZ domain had comparable flow speed and slightly reduced coherence as compared to WT (fig. S9, A, B, and D to F), while cortical flow speed and organization were both significantly reduced in PGCs with a mutated PH domain (fig. S9, C to E and G). Together, we propose that RhoGEF2 polarity and activity in PGCs require its PDZ and PH domains to potentiate RhoA activity and orient flow, with the PH domain additionally involved in driving flow.

### RhoGEF2-EB1 inhibition is relieved by phosphorylation

Our results thus far suggest that RhoGEF2 activity must be finely tuned to achieve accurate migration, as increasing or decreasing its activity impairs PGC guidance. While RhoGEF2 localization and activity are regulated via its PH and PDZ domains, it remains unclear how RhoGEF2 activity is spatially controlled in PGCs. Previous studies in insect culture cells showed that RhoGEF2 is inactive when bound to microtubule plus-ends and is liberated by Gα_12/13_, suggesting titration of free RhoGEF2 as a regulation strategy (Fig. 5A) (*43*). However, Gα_12/13_ is not necessary for PGC migration (Fig. 5A) (*28*) and deletion of the RGS domain in RhoGEF2, which is generally believed to mediate interaction with Gα_12/13_, did not affect PGC migration (Fig. 6P).

To first clarify whether RhoGEF2 is associated with microtubule plus-ends in PGCs (*43*), we isolated PGCs coexpressing EB1-GFP and red fluorescent protein (RFP)–RhoGEF2 (fig. S10, A and B). RFP-RhoGEF2 colocalized with EB1-GFP on comets emanating from a bright focus, likely a centrosome, and became cytoplasmic along with EB1-GFP upon microtubule depolymerization with colchicine (fig. S10, A and B). Colchicine-treated PGCs underwent extensive cycles of deformation and were significantly more dynamic than control PGCs (fig. S10, B and C, and movie S18), suggesting that RhoGEF2 release from microtubules increased contractility, in line with previous work (*43*). To better understand microtubule organization in migrating PGCs, we next performed live imaging with NOD-GFP, a microtubule minus-end marker (*44*). Microtubules extensively wrapped both actively migrating and arrested PGCs, and minus-ends were enriched at the cell rear (fig. S10D and movie S19), suggesting that microtubule growth occurs primarily from back to front. We confirmed this by imaging EB1-GFP, which additionally revealed that the centrosome, the microtubule-organizing center (MTOC) in PGCs, was positioned at the rear (fig. S10E and movie S20). Given that RhoGEF2 is enriched on centrosomes (fig. S10A) (*43*), the posterior localization of centrosomes in migrating PGCs could serve as a local reservoir of RhoGEF2.

**Fig. 7.**
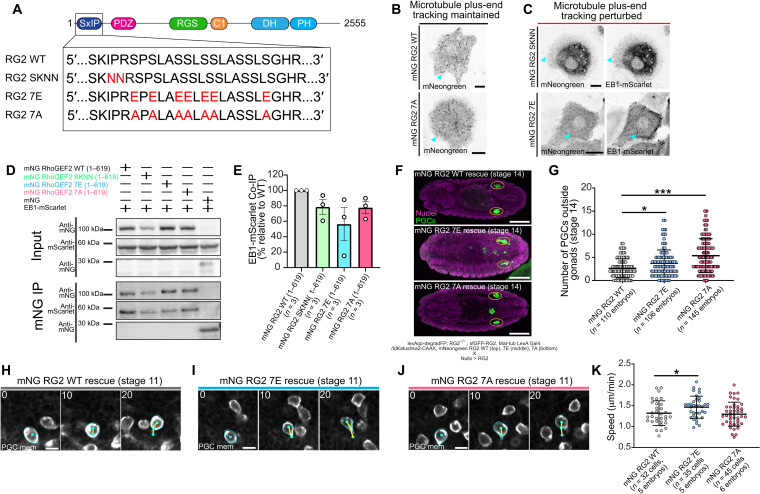
RhoGEF2 phospho-regulation controls free RhoGEF2 for accurate migration. (**A**) Schematic of the RhoGEF2 SKIP motif and protein domains along with the series of constructs created with mutagenesis. All constructs are driven as described in Fig. 6. (**B** and **C**) Representative images from insect S2 cells expressing the indicated mNeongreen RhoGEF2 constructs. EB1-mScarlet is coexpressed in (C) to identify microtubule plus-ends. Cyan arrows highlight microtubule plus-end tracking. Scale bars, 10 μm. (**D**) Representative immunoblot from a coimmunoprecipitation (co-IP) experiment from S2 cells overexpressing the indicated constructs. Cell lysates were immunoprecipitated with mNeongreen-trap and probed with the indicated antibodies. (**E**) Quantification of EB1-mScarlet co-IP with the indicated mNeongreen RhoGEF2 constructs. *n* = independent replicates. Error bars are SEM. (**F**) Representative immunofluorescence images from stage 14 embryos under the indicated rescue conditions. Yellow ovals mark the gonads. Genotypes are listed below the images. Scale bars, 100 μm. (**G** and **K**) Quantification of the number of PGCs outside gonads (G) or mean cell speed (K) under indicated rescue conditions. Error bars are SD. (**H** to **J**) Two-photon time-lapse imaging of representative PGCs migrating toward the mesoderm under the indicated rescue conditions. Cyan outlines cell membranes, cyan dots track PGC positions, and yellow lines are cell tracks. Scale bars, 10 μm. Times are in minutes. Statistical comparisons are pairwise from a Mann-Whitney test in (G) and (K). *P < 0.05 and ***P < 0.001.

Having outlined RhoGEF2 microtubule regulation in PGCs, we searched for an alternative, RGS-independent microtubule plus-end regulation mechanism to release RhoGEF2. We noted that RhoGEF2 has an N-terminal SxIP motif (SKIP), a motif that indirectly allows a variety of proteins to track microtubule plus-ends via interaction with EB1 (*45*). To establish whether the SKIP motif in RhoGEF2 is required for microtubule plus-end tracking, we introduced two point mutations previously shown to abrogate EB1 interaction (SKIP→SKNN) (Fig. 7A). Live imaging of insect cells expressing mNeongreen RhoGEF2 SKNN and EB1-mScarlet revealed that this construct was unable to track plus-ends, while the control construct did (Fig. 7, B and C). These results suggest the RhoGEF2 SKIP motif mediates microtubule plus-end tracking.

We next asked how the RhoGEF2 SKIP motif is regulated. SxIP motifs are typically flanked by numerous serine residues, which, when phosphorylated, can sterically inhibit EB1 interaction, allowing upstream regulation of microtubule plus-end release by a kinase (*45*). The RhoGEF2 SKIP motif is likewise surrounded by several serine residues, many of which are phosphorylated in the embryo, as indicated in a phospho-proteomic database from the Perrimon laboratory (Fig. 7A) (*46*). To determine whether multisite phosphorylation near the SKIP motif could affect RhoGEF2-EB1 interaction, we substituted glutamic acids or alanines for these seven proximal serines to create phospho-mimetic (7E) and phospho-null (7A) RhoGEF2 constructs, respectively (Fig. 7A). Using myosin II activation as a measure of RhoGEF2 activity, we noted that, upon overexpression in insect S2 cells, all constructs, including RhoGEF2 SKNN, exhibited slightly higher levels of myosin II activation as compared to WT at intermediate levels, but this was only significant for RhoGEF2 SKNN. At high expression levels, there were no significant differences with WT RhoGEF2, presumably because the buffering capacity of EB1 had been exceeded (fig. S11, A and B). Live imaging of insect cells coexpressing mNeongreen RhoGEF2 7E and EB1-mScarlet revealed that, similar to RhoGEF SKNN, RhoGEF2 7E could not track microtubule plus-ends (Fig. 7C), while the mNeongreen RhoGEF2 7A construct retained this ability (Fig. 7B). To further validate these conclusions, we performed coimmunoprecipitation (co-IP) experiments from S2 cells coexpressing truncated mNeongreen RhoGEF2 constructs with these mutations and EB1-mScarlet, with mNeongreen itself as a control. These truncated proteins maintained the microtubule plus-end phenotypes observed with the full-length protein (fig. S11, C to F). We found that the truncated RhoGEF2 SKNN, 7E, and 7A constructs all had a reduced affinity for EB1-mScarlet as compared to WT, with the 7E construct exhibiting the lowest affinity overall (Fig. 7, D and E). The reduced affinity of the 7A construct was unexpected, given its microtubule plus-end tracking in live imaging experiments (fig. S11F), and may reflect a change in protein behavior when extracted from a cellular environment. Our results collectively suggest that the free pool of signaling-competent RhoGEF2 can be titrated by multisite phosphorylation near the RhoGEF2 SKIP motif.

We next asked whether perturbing RhoGEF2-EB1 phospho-regulation had consequences for PGC migration. Following a similar strategy as above, we generated transgenic lines harboring mNeongreen RhoGEF2 SKNN, 7E, and 7A, which expressed at comparable levels to the WT (fig. S11, G and H) and polarized to a similar extent as WT in PGC clusters (fig. S11, I to N). However, in contrast to the ΔPDZ, ΔRGS, and PH domain mutant RhoGEF2 transgenes, these lines were able to rescue the lethality of RhoGEF2 mutants. Perturbing RhoGEF2-EB1 phospho-regulation impaired PGC homing, as there were significantly more PGCs that did not arrive at the gonad in stage 14 embryos when we performed rescue experiments, suggesting that this mechanism is necessary for PGC migration (Fig. 7, F and G). To investigate why PGC guidance was disturbed, we performed two-photon live imaging of PGCs expressing RhoGEF2 7E and 7A during cluster dispersal and migration toward the mesoderm. Although the cluster dispersal rate was similar under all conditions (fig. S11, O to R), PGCs expressing RhoGEF2 7E migrated significantly faster than WT (Fig. 7, H to K, and movie S21), suggesting that the increased free levels of RhoGEF2 accelerated migration speed. However, when comparing basal flow between extracted RhoGEF2 WT and 7E rescued PGCs, we found that there was no significant difference between flow speed and organization (fig. S12), suggesting that SKIP domain phospho-regulation regulates flow orientation, akin to the PDZ domain. The migration defects that we observed in the RhoGEF 7A rescue experiments, which do not perturb PGC speed, could arise from defects in PGC migration in developmental steps that we did not assess. Collectively, our findings suggest that dynamic, phosphorylation-mediated control of free RhoGEF2 levels controls flow orientation for directed migration.

### AMPK directly phosphorylates RhoGEF2 and is necessary for PGC guidance

**Fig. 8.**
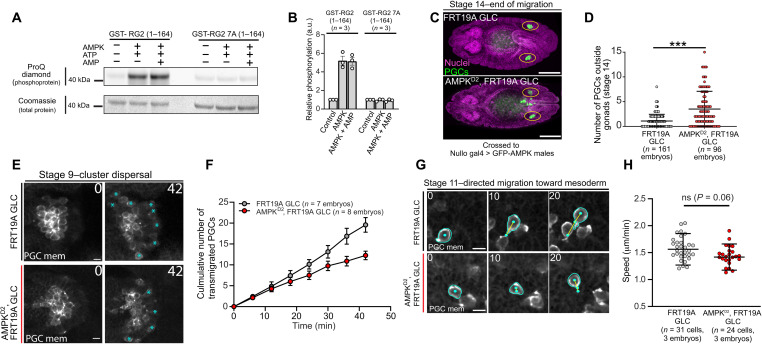
AMPK directly phosphorylates RhoGEF2 and is necessary for PGC migration. (**A**) Representative SDS–polyacrylamide gel electrophoresis from an in vitro kinase assay with purified AMPK enzyme and GST-RG2 fusion proteins under indicated conditions. The gel was probed with a ProQ diamond stain to visualize phosphorylated proteins and was subsequently stained with Coomassie blue to visualize total proteins. (**B**) Quantification of GST-RG2 protein phosphorylation relative to control under the indicated conditions. *n* = independent replicates. Error bars are SD. (**C**) Representative immunofluorescent images from stage 14 embryos with indicated female genotypes. Yellow ovals mark the location of the gonads. Scale bars, 100 μm. GLC, germline clone. (**D**, **F**, and **H**) Quantification of the number of PGCs outside gonads (D), transmigrated PGCs over time (F), or mean cell speed (H) under indicated conditions. Error bars are SD in (D) and (H) and SEM in (F). (**E** and **G**) Two-photon time-lapse imaging of representative PGC clusters expressing tdKatushka2-CAAX (PGC membrane marker) dispersing (E) or individual PGCs migrating to the mesoderm (G) with the described genotypes. Cyan asterisks mark transmigrated PGCs in (E), while cyan outlines cell membranes, cyan dots track PGC positions, and yellow lines are cell tracks in (G). Scale bars, 10 μm. Times are in minutes. Statistical comparisons are pairwise from a Mann-Whitney test in (D) and (H).

Last, we sought to determine the upstream kinase responsible for modulating RhoGEF2-EB1 binding to orient flow and migration. The iProteinDB database, which contains the phospho-proteomic data from *Drosophila* embryos and provides putative kinase predictions based on known motifs, predicted that adenosine monophosphate (AMP)–activated protein kinase (AMPK) would phosphorylate the serines proximal to the RhoGEF2 SKIP motif (*46*). AMPK is best known for its ubiquitous role in nutrient sensing but has recently been shown to regulate polarity and migration through phosphoregulation of the CLIP-170 microtubule-binding protein (*47*). In *Drosophila*, AMPK regulates epithelial polarity in the embryo and can phosphorylate myosin II, providing an alternate pathway to activate contractility (*48*). To determine whether AMPK can phosphorylate RhoGEF2, we performed an in vitro phosphorylation assay with purified AMPK holoenzyme and an N-terminal GST-RhoGEF2 fragment containing the SKIP motif and the nearby serines of interest (amino acids 1 to 164). A phosphorylation-specific staining revealed that AMPK readily phosphorylated GST-RhoGEF2 (1–164) specifically at the seven serines near the SKIP motif, as a GST-RhoGEF2 7A (1–164) fragment (seven serines changed to alanine) did not show a detectable change in signal intensity upon incubation with AMPK (Fig. 8, A and B). Having confirmed that AMPK can phosphorylate RhoGEF2 in vitro, we next asked whether removing AMPK would perturb PGC migration. We generated AMPK null germline clone embryos and rescued known somatic defects by driving a GFP-AMPK with *nullo*-Gal4. We observed a significant decrease in the number of PGCs that successfully reached the gonads in stage 14 embryos (Fig. 8, C and D), suggesting that AMPK was necessary for PGC migration. Live imaging revealed that these migration defects stemmed from a decreased cluster dispersal rate and slower migration speed, although the latter was not significant (Fig. 8, E to H, and movies S22 and S23).

**Fig. 9.**
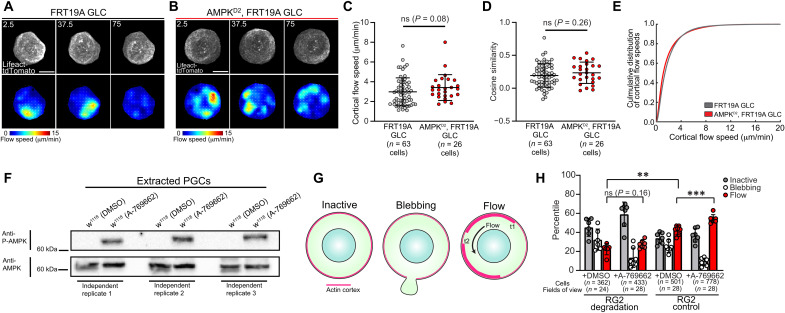
AMPK modulates cortical flow in a RhoGEF2-dependent manner. (**A** and **B**) Top: Time-lapse imaging of representative extracted PGCs under agarose with indicated genotypes expressing lifeact-tdTomato. Bottom: PIV flow analysis between consecutive images. Flow speed is color-coded with the indicated color bar. White arrows are flow vectors scaled to flow magnitude. Scale bars, 10 μm. Time is in seconds. (**C** and **D**) Quantification of mean cortical actin flow speed (C) and mean cosine similarity between vectors over consecutive time points (D) with the described genotypes. Error bars are SD. (**E**) Cumulative distributions of flow speeds with the labeled genotypes. (**F**) Representative immunoblot from PGCs extracted from a WT background (w^1118^) under the noted treatment conditions from three independent replicates. Cell lysates were first probed with phospho-AMPK antibody, stripped, and reprobed with total AMPK antibody. (**G**) Illustration of the classes of behavior shown by extracted PGCs in vitro. PGCs with stochastic accumulations of F-actin fell within the inactive category. (**H**) Quantification of the percentile of behaviors shown by extracted PGCs under the indicated treatment conditions with (RG2 control) and without (RG2 degradation) sfGFP-RG2. Representative images are shown in fig. S13. Individual data points are from quantifying the percentage of cell phenotypes per four view fields in a given experiment. Error bars are SD. Statistical comparisons are pairwise from a Mann-Whitney test in (C), (D), and (H). **P < 0.01 and ***P < 0.001.

**Fig. 10.**
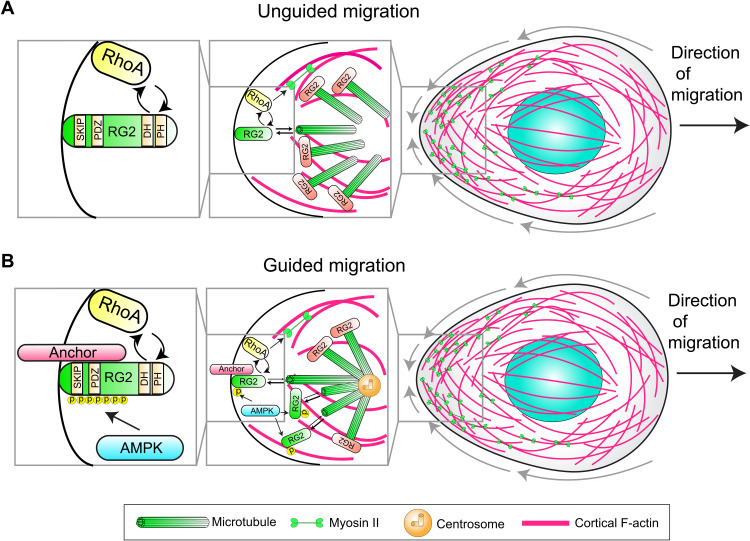
Model for RhoGEF2 cortical flow regulation with and without guidance from external cues. (**A**) In the absence of external guidance cues, the free pool of RhoGEF2 is set by its affinity to EB1. RhoGEF2 can stochastically accumulate on the membrane and use its basal activity to amplify RhoA signaling through a localization-based feedback loop involving its PH domain to regulate constitutive flow. (**B**) During guided migration, AMPK augments the free pool of RhoGEF2 by phosphorylating serines proximal to the SKIP motif to inhibit EB1 binding. The posteriorly localized centrosome may serve as a local reservoir of RhoGEF2. Free RhoGEF2 accumulates on the membrane by binding an unknown anchor with its PDZ domain and triggers RhoA activation with its basal activity. Nascent RhoA activation allows for the recruitment of more RhoGEF2 through a positive feedback by binding the RhoGEF2 PH domain, generating robust RhoA activation. RhoGEF2 activity levels alter the actomyosin contractility gradient in a cell to organize and drive cortical flow. This cortical flow module allows PGCs to orient their migration for accurate guidance in vivo.

To determine whether AMPK regulates basal cortical actin flow, we analyzed flows in extracted control and AMPK mutant PGCs. Similar to what we observed with RhoGEF2 7E rescued PGCs (fig. S12), we found no significant differences in flow speed and organization (Fig. 9, A to E, and movie S24), suggesting that AMPK regulates cortical flow under embryonic guidance cues. AMPK was not active in extracted PGCs but could be potentiated with the pharmacological AMPK activator A-769622 (Fig. 9F). Our ability to directly activate AMPK in extracted PGCs further allowed us to test whether AMPK can directly modulate cortical flow and if it does so in a RhoGEF2-dependent manner. We isolated RhoGEF2-depleted and control PGCs and compared their response phenotypes to AMPK activation (Fig. 9, G and H). Upon dimethyl sulfoxide (DMSO) (control) treatment, the percentage of RhoGEF2-depleted PGCs displaying circular flow was significantly decreased compared to control PGCs, confirming our prior observation that RhoGEF2 regulates basal flows (Fig. 4, J to N; fig. S13; and movies S25 and S26). Notably, AMPK activation led to a significant increase in PGCs displaying circular cortical flow in control but not RhoGEF2-depleted PGCs, suggesting that AMPK can directly regulate cortical flow in a RhoGEF2-dependent manner (Fig. 9H, fig. S13, and movies S25 and S26). On the basis of these results, we propose that AMPK spatiotemporally controls RhoGEF2 activity to orient flow for accurate homing in vivo (Fig. 10).

## DISCUSSION

Many cells have the latent potential to adopt protrusion-independent, amoeboid global cortical flow migration within confined, nonadhesive environments that promote contractility (*5*). The hypothesized in vivo function of this type of migration is to allow directional migration away from a contractile stimulus (*6*), such as a wound, and/or to rapidly escape confinement through nuclear mechanosensation (*11*, *16*). In this study, we develop optimized imaging approaches and show that *Drosophila* PGCs endogenously use global cortical flow migration to navigate in vivo. While PGCs experience a confined cellular environment in vivo, we find that PGCs maintain cortical flows in vitro without confinement and serum stimulation (Fig. 2, A and B), suggesting that cell-intrinsic properties, such as basal actomyosin contractility, are themselves sufficient. These cortical flows are strongly dependent on linear actin polymerization through formins (Fig. 2, I to L), in contrast to the branched actin-dependent retrograde actin flows used by mesenchymal cells (*26*, *49*). Other factors that may contribute to the global flow that we observe include the physical properties of the PGC actin cortex itself. Work in the *Caenorhabditis elegans* zygote has indicated that cortical viscosity can determine whether a local contraction induces a long-range flow (*13*). Our work also points to cortical architecture as a determinant (Fig. 1, F to I, and fig. S1, B and C), as changes in cortical connectivity can influence the length scale of flow propagation (*50*, *51*). Elegant in vitro studies using reconstituted actin cortices have established that a percolation threshold exists such that intermediate levels of actin crosslinking are required for flow propagation across the entire network (*52*). Future studies need to determine the cortical properties and architecture that enable global cortical flow in PGCs.

PGC cluster dispersal and transepithelial migration depend on the GPCR Tre1 (*24*, *25*, *28*–*30*), which radially orients migration outward, implying that GPCR signaling can orient cortical flow. How this mechanistically occurs is unclear, but it is likely to be distinct from the detailed GPCR-mediated pathways outlined in neutrophils and *Dictyostelium*, which follow the protrusive front and contractile rear paradigm. We have previously shown that active CDC42 and Rac are not polarized in PGC clusters (*24*) and that overexpression of constitutively active or dominant-negative CDC42 does not impair migration (*25*). Others have also noted that phosphatidylinositol 3,4,5-trisphosphate [PI(3,4,5)P_3_], a phosphoinositide important for orienting protrusion-driven cells, is similarly not polarized (*30*). One common strategy to orient flow is to locally inhibit contractility, thereby creating an actomyosin contractility gradient that drives flow toward the opposite end of the cell. In both the *C. elegans* zygote and *Xenopus* oocyte, local inhibition occurs proximal to the microtubule-organizing center, suggesting a role for microtubules in this process, although this remains unclear in *C. elegans* (*53*, *54*). Centrosomes are instead located near regions of high contractility in PGCs (fig. S10E), but GPCR signaling may specify an axis of motility through a similar mechanism by determining which regions should not be the “rear.”

Our work establishes a RhoGEF regulatory logic responsible for tuning cortical flow (Fig. 10). During unguided migration, the amount of free RhoGEF2 is set by its affinity for EB1. RhoGEF2 stochastically accumulates on the membrane and triggers nascent sites of RhoA activity, which are further amplified by a PH domain–dependent positive feedback loop (Fig. 10A and fig. S8, A to C). When RhoGEF2 is depleted, cortical actin flows are slower and more disorganized (Fig. 4, J to N), suggesting that RhoGEF2 establishes an actomyosin contractility gradient to induce sufficiently fast cortical flows for accurate migration. The RhoGEF regulation that we uncover here does not require Gα_12/13_ and thus expands our knowledge of how RGS-RhoGEFs are regulated in space and time. We speculate that this feedback loop could similarly be important for PDZ-RhoGEF, which operates at the rear of migrating neutrophils (*18*).

We further find that regulating RhoGEF2 availability allows potential directional control of cortical flow (Fig. 10B). Under embryonic guidance cues, AMPK directly phosphorylates RhoGEF2 near its N-terminal SKIP motif, augmenting the free pool of RhoGEF2 by liberating it from EB1-dependent inhibition (Figs. 8, A and B, and 9F). RhoGEF2 is then anchored at the membrane through its PDZ domain and establishes a zone of high RhoA activity through a feedback between RhoA and its PH domain to orient flow and migration (Fig. 10B). Disrupting RhoGEF2 phosphoregulation impairs pathfinding, suggesting that PGCs need to orient and/or tune flow during their journey. Globally regulating AMPK activity, alternatively, could allow PGCs to modulate their constitutive cortical flow speeds to, for example, help PGCs stop once they reach their target by decreasing flow. Another plausible scenario is that PGCs may need to accelerate cortical flow to maintain their speed on different cellular substrates, such as when transitioning from an E-cadherin–expressing endoderm to an N-cadherin–expressing mesoderm. Such adaptive dynamics have been demonstrated in dendritic cells (*55*).

Our findings further expand the burgeoning role of AMPK in cell migration. Such regulation has precedence, as AMPK has previously been shown to phosphorylate CLIP-170 to reduce its affinity for microtubules (*47*). Disrupting CLIP-170 phosphoregulation perturbs migration (*47*), as we have similarly observed for RhoGEF2 in PGCs.

To conclude, the role of global cortical flow migration in vivo has been proposed to be a specialized response to external stimuli to enable rapid, context-dependent persistent motility (*6*). We find that this migration is the prevalent mode by which *Drosophila* PGCs move and that it is receptive to external guidance, conceptually similar to recently described directed fat body cell movement (*56*). Organization and dynamics of cortical flow depend on two pathways coordinated within one molecule, RhoGEF2. AMPK-regulated phosphorylation releases RhoGEF2 from microtubule-dependent inhibition, while membrane localization and local amplification of RhoA activity via RhoGEF2’s PH and PDZ domains lead to rear-end myosin II contraction and rearward flow of cortical actin. How this activation cascade is receptive to external guidance via GPCR signaling remains unclear. Our results further show how RhoGEF2 can be spatially regulated independently of Gα_12/13_ and its RGS domain. Instead, we demonstrate a novel role for AMPK in regulating RhoGEF2 to orient PGC migration (Fig. 10). Given the widespread use of amoeboid migration, a choice between Gα_12/13_ and AMPK-mediated RhoGEF2 regulation may more generally account for dynamic migratory responses under changing environmental conditions.

## MATERIALS AND METHODS

### Fly strains

All fly strains were maintained at 25°C, with experimental genotypes listed in table S2. w^1118^ was used as the negative control. Transgenic lines were produced by Bestgene Inc. using phiC31 integrase–mediated transgenesis. The landing sites used in this study were su(Hw)attP8 on the X chromosome, attP40 and su(HW)attP5 on the second chromosome, and attP2, VK27, VK28, and VK33 on the third chromosome.

### Constructs

Infusion (Clontech) cloning was used to create all constructs, and Q5 High-Fidelity DNA polymerase (New England Biolabs) was used for polymerase chain reaction (PCR). The *nos* and UAS with *nos*TCE-*pgc* 3′UTR (allows PGC targeting) backbones have previously been described (*24*). pJFRC19 [G. Rubin (*57*); Addgene, 26224] was the backbone for LexAop-driven constructs. pJFRC19 was modified to permit efficient, targeted expression in PGCs by removing the hsp70 promoter and SV40 3′UTR with a restriction digest and replacing them with the p-element promoter from pwalium22 and *nos*TCE-*pgc* 3′UTR, respectively. The *squash* (myosin II RLC in *Drosophila*) promoter–driven RhoGEF2 constructs used pWALIUM22 [Perrimon laboratory (*58*)] as a backbone. The UAS sites and K10 3′UTR were replaced via PCR with the *squash* promoter and *squash* 3′UTR from pBS-Squ-mCherry [E. Wieschaus (*59*); Addgene, 20163]. The RhoGEF2 open reading frame (ORF) was obtained from the Drosophila Genomics Research Center (DGRC) (SD04476). All mutations were introduced with site-directed mutagenesis (Q5 site-directed mutagenesis kit, New England Biolabs, E0554S). pGEX6P1-N-HA (A. Jackson and M. Reijns; Addgene, 119756) was used as the backbone for recombinant protein expression in *Escherichia coli*. All constructs were sequence-verified before sending for injection.

#### LexAop2-p-degradFP-nosTCE-pgc 3′UTR

The degradFP system [M. Affolter (*31*); Addgene, 35579] was amplified by PCR and inserted into pJFRC19 containing the p-element promoter and *nos*TCE-*pgc* 3′UTR as described above. Fly lines were generated on su(Hw)attP8, attP40, and attP2.

#### Mat-tub LexA-GAD

Three fragments were cloned into pWALIUM22 with UAS sites and K10 3′UTR removed: (i) α*Tub67C* promoter and 5′UTR were amplified via PCR from genomic DNA from P(matα4-GAL-VP16)67; P(matα4-GAL-VP16)15 females (BL 80361), (ii) nlsLexA::GADfl ORF was amplified from pBPnlsLexA::GADflUw [G. Rubin (*57*); Addgene, 26232], and (iii) α*Tub84B* 3′UTR was ordered as a Gblock from IDT Technologies. Fly lines were generated on attP40 and attP2.

#### UASp-Gα_12/13_-Q303L-nosTCE-pgc 3′UTR

The Gα_12/13_ (known as concertina in *Drosophila*) ORF (DGRC, LD04530) was amplified via PCR and cloned into pWALIUM22 containing the *nos*TCE-*pgc* 3′UTR described previously. Site-directed mutagenesis was then used to generate the Q303L mutation. Fly lines were generated on attp40 and attP2.

#### Nos-myosin-II-tdTomato-P2A-tdKatushka2-CAAX

*Nos* regulator elements drive the myosin II RLC [E. Wieschaus (*59*); Addgene, 20163] fused to tdTomato [M. Davidson (*60*); Addgene, 54653], a P2A peptide, and tdKatushka2 [M. Davidson (*60*); Addgene, 56041] with a CAAX box from human KRAS for membrane targeting. Three fragments were amplified via PCR and cloned into pWALIUM22 with *nos* regulatory elements described previously. The P2A and CAAX sequences were added to tdKatushka2 via primer. Fly lines were generated on attP40 and su(HW)attP5 on the second chromosome and attP2 and VK27 on the third chromosome.

#### Squ-mNeongreen-RhoGEF2 constructs

For all constructs, mNeongreen (allele biotech) and RhoGEF2 (DGRC, SD04476) were first subcloned into pUC19 and sequence-verified before subsequent cloning as a single fragment into pWALIUM22 with *squash* regulatory elements described above. For squ-mNeongreen-RhoGEF2-ΔPH RBD, the Anillin RBD was subcloned from the nos-tdTomato-Anillin-RBD-P2A-tdKatushka2-CAAX transgene generated previously (*61*). Fly lines were generated on attP2, VK27, VK28, or VK33 on the third chromosome.

#### pAc EB1-mScarlet

EB1 (DGRC, RE41364) and mScarlet [D. Gadella (*62*); Addgene, 85042] were amplified with PCR and cloned into Ac5-Stable2-neo [R. Barrio and J. Sutherland (*63*); Addgene, 32426] with inserts removed by PCR.

#### pAc mNeongreen RhoGEF2 (1–619) constructs

These constructs were subcloned via PCR from the corresponding squ-mNeongreen-RhoGEF2 full-length constructs into Ac5-Stable2-neo [R. Barrio and J. Sutherland (*63*); Addgene, 32426].

#### pGEX-RG2-164, pGEX-RG2-164 7A

These constructs were subcloned via PCR from the corresponding squ-mNeongreen-RhoGEF2 full-length constructs into pGEX6P1-N-HA (A. Jackson and M. Reijns; Addgene, 119756).

#### pGEX-Rho1

The Rho1 ORF (DGRC, LD03419) was cloned into pGEX6P1-N-HA (A. Jackson and M. Reijns; Addgene, 119756).

### Live imaging

Embryos were produced at 25°C. For live imaging experiments from embryos, embryos were first dechorionated in 50% bleach for 3 min, extensively washed, collected onto a nylon mesh, and placed onto apple juice agar plates for visual staging. Appropriately staged embryos were subsequently oriented with their dorsal surface facing up with a fine needle, adhered to #1.5 glass coverslips (Thermo Fisher Scientific, 12-544-BP) with heptane glue, overlaid with halocarbon oil 27 (Sigma-Aldrich, H8733), and placed onto a gas-permeable membrane (YSI, 098094). Live embryo imaging was performed on a custom Prairie Ultima (Bruker technologies) using a Nikon CFI Apo IR 60× 1.27 numerical aperture (NA) water objective, a Chameleon Discovery tunable femtosecond laser with total power control, and four external GaAsp photomultiplier tubes (Hamamatsu, 7422PA-40), and driven by Prairie View 5.0 software.

A four-channel upper nondescanned detector module was used for simultaneous acquisition of four channels. An initial dichroic (t560lpxr) split the emission to two custom filter cubes: (i) band-pass filters ET575/50m-2p and ET660/60M-2p with a T612LPXR-UF1 dichroic to simultaneously detect the emission from the fluorescent proteins tdTomato and tdKatushka2 used in this study and (ii) band-pass filters ET460/50m-2p and ET525/50m-2p with a T495lpxr dichroic to detect the emission from the fluorescent proteins sfGFP and mNeongreen used in this study. The fourth channel, for detecting emission from fluorescent proteins such as CFP, was not used in this study. A 1080-nm wavelength was used for experiments where PGCs expressed reporters with tdTomato and tdKatushka2, while a 950-nm wavelength was used when PGCs coexpressed a given sfGFP or mNeongreen-tagged transgene. All three fluorescent proteins (sfGFP/mNeongreen, tdTomato, and tdKatushka2) could be detected under sufficiently strong laser power at 950 nm. All filters were purchased from Chroma Technology Corp.

Live imaging of extracted PGCs and insect S2 cells in vitro was performed on a Nikon W1 spinning disk confocal microscope with an SR HP Plan Apo 100× 1.35 NA objective and Andor 888 electron-multiplying charge-coupled device (EMCCD) cameras driven by Nikon Elements software. The experimental setup is described below.

### PGC purification and experimentation

We capitalized on the early formation of PGCs before somatic cellularization is complete to develop an efficient protocol to specifically extract PGCs. We based our protocol off an existing protocol for FACS (fluorescence-activated cell sorting) sorting *Drosophila* embryonic cells (*64*). Embryos were first aged en masse to stage 5 of embryogenesis, homogenized in a Dounce homogenizer in Schneider’s medium (Thermo Fisher Scientific, 21720024), filtered through a 100-μm mesh to separate large embryonic fragments, centrifuged (500*g* for 1 min), exchanged into hemolymph-like buffer [25 mM KCl, 90 mM NaCl, 4.8 mM NaHCO_3_, 80 mM d-glucose, 5 mM trehalose, and 10 mM Hepes (pH 6.9)] with 0.25% trypsin, and incubated in a 37°C water bath for 10 min to break up any cell clumps. The trypsin was then neutralized by adding Schneider’s medium with 10% fetal bovine serum (FBS), the cell suspension was centrifuged, and the medium was exchanged for Schneider’s medium with 1% (w/v) bovine serum albumin (BSA).

For experiments without compression, PGCs were seeded directly onto an eight-well #1.5 glass Lab-Tek slide (Nunc) and allowed to settle for 1 hour before imaging. Treatment with A-769662 (300 μM; Sigma-Aldrich, SML2578) was conducted after PGCs had adhered, and imaging was performed after a 1-hour incubation. For under agarose experiments, a polydimethylsiloxane stencil was first created by curing Sylgard 184 (Dow) at a 1:10 crosslinker to polymer ratio and punching a central hole with a 14-mm punch. The stencil was then aligned and placed over the well of a 14-mm #1.5 glass bottom dish (MatTek). A 1% (w/v) ultrapure agarose (Thermo Fisher Scientific, 16500100) in phosphate-buffered saline (PBS) solution was then placed within the stencil and allowed to cure at room temperature. After the gel solidified, one end of the gel was gently picked up with forceps, the PGC cell suspension was pipetted into the well, and the gel was gently placed back down. Schneider’s medium was then pipetted around the gel. For experiments with cytochalasin D (100 μM; Sigma-Aldrich, C8273), Y-27632 (100 μM; Millipore Sigma, 688001), CK-666 (100 μM; EMD Millipore, 182515), and SMIFH2 (50 μM; EMD Millipore, 344092), the drugs were diluted in Schneider’s medium, added around the gel in the dish, and allowed to incubate at room temperature for at least 1 hour before imaging.

### PIV analysis

All PIV analysis was performed in MATLAB R2021a (MathWorks) using PIVLab (*65*, *66*) with the following analysis settings: (i) image preprocessing: CLAHE window size, 10 pixels; (ii) PIV settings: fast Fourier transform window deformation: pass 1, 64 pixels; step, 32 pixels; pass 2, 32 pixels; step, 16 pixels; pass 3, 16 pixels; step, 8 pixels; subpixel estimator: Gauss, 2 × 3-point; correlation robustness: standard; (iii) postprocessing: velocity-based validation: standard deviation filter, 8; local median filter, 3; image-based validation: filter low contrast—this parameter was tuned between 0.005 and 0.02 depending on a given cell.

All results were exported to MATLAB and subsequently analyzed and visualized using custom-written code. For average cortical flow speed, the average vector magnitude of the 10 fastest vectors for a given *xy* position across all time points was averaged to account for flows sweeping across different regions of a given PGC. For cumulative distributions, all vector magnitudes at each *xy* position across all time points were aggregated. For cosine similarity analysis, the cosine similarity between vectors at each *xy* position at consecutive time points was computed with the following formula

$$\text{sim}(V_{t1},V_{t2})=\frac{V_{t1}\cdot V_{t2}}{\left\|V_{t1}\|\;\|V_{t2}\right\|}$$

The computed cosine similarities were then averaged to obtain the mean value for each cell.

### Image processing and analysis

Two-photon images presented in figures and movies were denoised using the CANDLE (*67*) package for MATLAB in MATLAB 2016a (MathWorks). The settings used were beta = 0.3, patch radius = 2, and search radius = 2. All quantitative analysis was performed on raw data. For insect S2 or PGC in vitro imaging, a Gaussian filter (Sigma adjusted between 0.05 and 1) was applied for denoising for figure presentation.

To quantify actin cortical flow in vivo, kymographs were produced for five actin clusters per cell and used to estimate flow speed. The mean of the five clusters was taken as the actin flow speed. Cell speed was computed by creating a kymograph with the membrane images. In small Rho GTPase overexpression experiments, flow speed was also quantified using kymographs, generally generated on five distinct actin structures. Some PGCs had fewer structures, and in this case, this lower number was averaged. To quantify myosin II cortical flow in vivo, a kymograph was used to estimate the flow speed of a single myosin II foci. Cell speed was calculated as described above.

To quantify the period of circular flows in PGCs, a kymograph was generated across the cell from the myosin II image and the resulting distance between three high-intensity regions, indicating flow passing through this region, was used to calculate the period. We defined cells as exhibiting periodic cortical flows if the flows traveled around the circumference of the cell at least three times during imaging. Other PGC phenotypes were classified on the basis of the following criteria: (i) blebbing PGCs generated blebs identified as cytoplasmic extrusions from the cell body without cortical actin, (ii) stochastic PGCs exhibited brief crescents of F-actin and myosin II that did not travel, and (iii) inactive PGCs did not display any coincident crescents of F-actin and myosin II. In experiments with A-769662, we counted a given PGC as displaying cortical flow if flow swept across the cell perimeter at least once within the imaging time frame. Inactive and stochastic PGCs were binned into the same category.

For cell tracking and quantification of cell speed in ImageJ and MATLAB, regions of interest (ROIs) were first defined in ImageJ for cell segmentation. ROIs were subsequently imported into MATLAB, and cells were automatically segmented from ROIs using the “func_threshold” function (*68*) from all *Z* planes. The cell centroid was calculated as the average *XYZ* pixel value for all segmented pixels. Only cells that exhibited a persistence (minimal distance between initial and endpoint/total distance traveled) > 0.5 were used in analysis. All other analysis was completed using a combination of ImageJ and custom-written scripts in MATLAB described below.

Quantification of polarized myosin II in cells has been previously described (*24*). Briefly, regions of high myosin II (at least ≥1.2-fold greater intensity than cytoplasm) along with a corresponding region in the cytoplasm were manually segmented in ImageJ and imported into MATLAB. The computed ratios between these ROIs were taken as the polarized intensity. Quantification of polarized myosin II orientation with respect to the endoderm center in individual cells has been previously described (*24*). Briefly, the angle between the following line segments was used to calculate orientation: (i) PGC nuclei and region of polarized myosin II (defined above) and (ii) PGC nuclei and endoderm center.

Quantification of fluorescence intensity as a function of distance from cluster centroid has previously been described (*24*). Briefly, individual PGC clusters were manually segmented in ImageJ with the PGC-specific membrane marker (tdKatushka2-CAAX) along with a second ROI in the background for normalization. The ROIs were then imported into MATLAB and used as masks for the other fluorescent channels of interest. Pixel intensities were then placed into 50 equally spaced bins according to distance from the cluster centroid.

Quantification of posterior myosin II intensity over time has previously been described (*24*). Briefly, individual PGCs were manually segmented in ImageJ in the *Z* plane, which contained the greatest polarized myosin II intensity. ROIs were subsequently imported into MATLAB, segmented PGCs were computationally rotated vertically with posterior toward the bottom, and posterior was defined as the lowest 20% of segmented rows. The mean intensity in this posterior region over all time points was used as posterior myosin II intensity.

To quantify sfGFP-RhoGEF2 degradation, an ROI was defined in the central plane of the PGC cluster using the PGC membrane marker (tdKatushka2-CAAX) in ImageJ. This ROI was then used as a mask for the sfGFP-RhoGEF2 channel, and the mean intensity of the segmented pixels was quantified.

To quantify phospho–myosin II intensity in fixed S2 cells as a function of mNeongreen RhoGEF2 construct expression, maximum intensity projections were first generated from Z stacks taken from S2 cells expressing a given mNeongreen RhoGEF2 construct and stained for phospho–myosin II. Each cell was manually segmented using the mNeongreen signal with an ROI to obtain the mean construct expression level, and this ROI was subsequently used as a mask to generate the mean phospho–myosin II intensity. Each individual cell’s construct expression level and corresponding mean phospho–myosin II intensity were then placed into three equally spaced bins (low, medium, and high). The bins were equivalent across experimental conditions in the same plots.

To quantify cellularization furrow intensity in stage 5 embryos, 10 somatic furrow tip intensities were calculated by manual segmentation from PGC membrane marker images (this reporter has low expression in somatic cells) and normalized to the intensity of the top of the furrow, near the apical surface of the cell. To quantify centroid displacement, PGCs were automatically segmented with intensity-based thresholding in MATLAB and the centroids of the segmented PGCs were tracked over time. To quantify PGC dispersal rate, PGCs trans-migrating across the endoderm were manually annotated from time-lapse image stacks. To quantify immunoblot or gel images, ROIs were defined in ImageJ around each band and the integrated density was extracted and normalized to the loading control or input.

### Cell culture and transfection

S2 cells were cultured in Schneider’s medium with 10% (v/v) FBS (Thermo Fisher Scientific, 16140071) and 1% (v/v) penicillin-streptomycin (Thermo Fisher Scientific, 15140122). Effectene (Qiagen, 301425) was used for all transfections following the manufacturer’s recommendations. Transfected S2 cells were plated onto Lab-Tek slides (Thermo Fisher Scientific, 155409) coated with concanavalin A (50 μg/ml) (Cayman Chemical, 14951) diluted in PBS for 1 hour at room temperature for live imaging. For immunofluorescence, S2 cells were seeded onto 16-mm circular concanavalin A–coated coverslips (same as above) for at least 1 hour before proceeding to immunostaining.

### Antibodies

Primary antibodies used in this study for immunofluorescence are the following: rabbit anti-vasa (1:5000; R. Lehmann), guinea pig anti–phospho–myosin II (1:1000; R. Ward), chicken anti-vasa (1:500; R. Lehmann), and rabbit anti-RhoGEF2 (1:2500; J. Grosshans).

Secondary antibodies used in this study for immunofluorescence are the following: Cy3 AffiniPure Donkey Anti-Rabbit IgG (immunoglobulin G) (Jackson ImmunoResearch, 711-165-152), Cy3 AffiniPure Donkey Anti-Guinea Pig IgG (Jackson ImmunoResearch, 706-165-148), Alexa Fluor 488 AffiniPure Donkey Anti-Rabbit IgG (Jackson ImmunoResearch, 711-545-152), and Alexa Fluor 488 AffiniPure Donkey Anti-Chicken IgY (Jackson ImmunoResearch, 703-545-155).

Primary antibodies used for immunoblotting are the following: mouse anti-mNeongreen (1:1000; Chromotek, 32F6), rabbit anti-mNeongreen (1:1000; Cell Signaling Technology, 53061), mouse anti–α-tubulin (1:4000; Sigma-Aldrich, T6199), mouse anti-RFP (1:1000; Chromotek, 6G6), rabbit anti-GST (1:2000; Cell Signaling Technology, 2622), rabbit anti–phospho-AMPK (1:1000; Cell Signaling Technology, 2535), and mouse anti-AMPK (1:3000; Bio-Rad, MCA2672GA).

Secondary antibodies used in this study for immunoblotting are the following: horseradish peroxidase (HRP) goat anti-rabbit IgG (1:10,000; Abcam, ab6721) and HRP rabbit anti-mouse IgG (1:10,000; Abcam, ab6728).

### Immunofluorescence

Embryos were first dechorionated in 50% bleach for 3 min, extensively washed, collected on a nylon mesh, and transferred to a scintillation vial containing a 1:1 (v/v) mixture of heptane and 4% paraformaldehyde (Electron Microscopy Sciences, 15714-S) in PBS on a shaker for 20 min. The paraformaldehyde was subsequently removed with a Pasteur pipette and replaced with methanol, and the scintillation vial was vigorously agitated by hand for 30 s to remove the vitelline membrane. Embryos were kept in methanol at −20°C until subsequent processing. Embryos stored in methanol were gradually rehydrated with PBS–Triton X-100 (PBST) [0.3% Triton X-100 (Sigma-Aldrich, T8787)] and blocked in PBST with 1% BSA (Sigma-Aldrich, A4503) for 60 min at room temperature. All primary antibodies were diluted in PBST with 1% BSA and applied overnight at 4°C. After extensive washing, appropriate secondary antibodies (1:500; Jackson ImmunoResearch) were diluted in PBST with 1% BSA and incubated with samples for 3 hours at room temperature. Embryos were washed and subsequently mounted in VECTASHIELD (Vector Laboratories, H-1000) and imaged with Zeiss LSM 800 using Zen Blue 2.3 with a 20× 0.8 NA air objective using a pinhole size of 1 Airy unit.

Insect S2 cells were fixed in 4% paraformaldehyde, briefly permeabilized with 0.1% Triton X-100, washed, and blocked overnight in 5% (w/v) BSA in PBS. Primary and secondary antibodies, diluted in 5% BSA in PBS, were then applied at room temperature for 1 hour with extensive washes between these steps. The samples were then mounted with ProLong Diamond (Molecular Probes, P36965) onto glass slides and imaged with a Nikon W1 spinning disk confocal microscope with an Apo 60× 1.40 NA oil objective and Andor 888 EMCCD cameras driven by Nikon Elements software.

### In vitro translation

The TNT T7 Quick Coupled Transcription/Translation System (Promega, L1170) with Transcend tRNA (Promega, L5061) was used to in vitro translate the RhoGEF2 PH domain according to the manufacturer’s recommendation.

### Coimmunoprecipitation

S2 cell lysates were harvested 3 to 4 days after transfection in co-IP buffer [50 mM tris-HCl (pH 7.5), 250 mM NaCl, 1 mM EDTA, 1 mM MgCl_2_, 0.2% NP-40, and 10% glycerol] with protease/phosphatase inhibitors (Thermo Fisher Scientific, 78442), incubated on ice for 30 min, clarified by centrifugation, and measured with a bicinchoninic acid (BCA) assay (Thermo Fisher Scientific, 23225). Equal amounts of lysate (1 mg) were then immunoprecipitated with mNeongreen-trap magnetic agarose resin (Chromotek, ntma) for 1 hour at 4°C, washed, and then eluted with lithium dodecyl sulfate (LDS) buffer (Thermo Fisher Scientific, NP0007).

### GST protein purification

pGEX-Rho1, pGEX-RG2-164, pGEX-RG2-164 7A, and pGEX6P1-N-HA were transformed into BL21(DE3)-competent cells (New England Biolabs, C2527H) and grown overnight at 37°C in a starter culture. The starter cultures were then diluted 1:200 into 1-liter cultures, shaken for ~1.5 hours at 37°C until reaching an OD_600_ (optical density at 600 nm) of 0.6 to 0.8, induced with 100 μM isopropyl-β-d-thiogalactopyranoside (IPTG), and shaken for 20 to 24 hours at room temperature. The bacterial cultures were then pelleted by centrifugation, washed once with PBS, and flash-frozen and stored at −80°C until further processing. To harvest recombinant GST proteins, frozen bacteria pellets were lysed in lysis buffer [20 mM Hepes (pH 7.5), 150 mM NaCl, 5 mM MgCl_2_, 1% Triton, and 1 mM dithiothreitol (DTT) with protease/phosphatase inhibitors], sonicated in Bioruptor Pico (Diagenode), and clarified by centrifugation at 4°C. The clarified lysate was then incubated with Glutathione Sepharose 4B resin (Millipore Sigma, GE17-0756-01) for 2 hours at 4°C, washed, and then resuspended in storage buffer (Hepes buffered saline, 5 mM MgCl_2_, 1 mM DTT) with 33% glycerol.

### In vitro kinase assay

GST-RG2-164 and GST-RG2-164 7A were eluted from Glutathione Sepharose 4B resin with elution buffer [50 mM tris-Cl (pH 8) and 30 mM glutathione] and concentrated and exchanged into Hepes-Brij buffer [50 mM Na-Hepes (pH 7.4), 1 mM DTT, 5 mM MgCl_2_, and 0.02% Brij-35] with an Amicon Ultra-2 10K NMWL filtration unit (UFC201024). Protein concentrations were then estimated by SDS–polyacrylamide gel electrophoresis (PAGE) with BSA standards followed by InstantBlue Coomassie stain (ab119211). In vitro kinase assays were performed with ~2 μg of substrate, 50 ng of AMPK holoenzyme (EMD Millipore, 14-840), 0.2 mM adenosine triphosphate (ATP) (EMD Millipore, 1191-5GM), and 0.3 mM AMP (EMD Millipore, 118110-5GM) where indicated for 30 min at 30°C. Reactions were terminated by adding LDS buffer, and samples were separated by SDS-PAGE. Phosphorylated proteins were detected with a ProQ diamond stain (Thermo Fisher Scientific, P33302) using a modified protocol (*69*), and total proteins were subsequently detected with InstantBlue Coomassie stain.

### In vitro binding assay with GST-Rho1

Ten micrograms of GST-Rho1 per reaction was first resuspended in nucleotide exchange buffer [50 mM Hepes (pH 7.08), 5 mM EDTA, 0.1 mM EGTA, 50 mM NaCl, and 0.1 mM DTT] and loaded with 0.5 mM GDP (Sigma-Aldrich, G7127) or GTPγS (Cytoskeleton, bs01) for 30 min at 30°C. The reaction was terminated by adding 20 mM MgCl_2_. GST-Rho1-GDP and GST-Rho1-GTPγS were then exchanged into Hepes-LS buffer [20 mM Hepes (pH 7.5), 150 mM NaCl, 10% glycerol, and 0.1% Triton X-100] and incubated with ~1 μg (estimated by SDS-PAGE) of in vitro translated RG2-PH domain for 1 hour at 4°C. Following washes, the remaining bound products were eluted with LDS buffer.

### Protein extraction from embryos

Overnight embryo collections were dechorionated in 50% bleach for 3 min and transferred with a paint brush to ice-cold radioimmunoprecipitation assay (RIPA) buffer (Thermo Fisher Scientific, 89900) with protease/phosphatase inhibitors in a 1.5-ml Eppendorf tube. The embryos were subsequently homogenized with a motorized pestle and incubated with periodic mixing for 30 min on ice. The lysates were then clarified by centrifugation, and protein concentrations were measured with a BCA assay for subsequent SDS-PAGE.

### Protein extraction from PGCs

PGCs were purified from embryos using the protocol described above, resuspended in Schneider’s medium containing either control (DMSO) or A-769662 (300 μM), and seeded onto a non–tissue culture–treated plates to prevent cell adhesion. After a 1-hour incubation, PGCs were detached by pipetting, spun down, resuspended in ice-cold RIPA buffer with protease/phosphatase inhibitors, and incubated on ice for 15 min. The lysates were sonicated with Bioruptor Pico and clarified by centrifugation, and protein concentrations were measured with a BCA assay before proceeding to SDS-PAGE.

### SDS-PAGE and immunoblotting

Equal amounts of protein were separated via SDS-PAGE using a 1.5-cm 4 to 12% bis-tris gel (Thermo Fisher Scientific, NP0336) or 1-cm 4 to 12% bis-tris gel for ProQ diamond stain with 1× Mops running buffer (Thermo Fisher Scientific, NP0001), transferred onto a polyvinylidene difluoride membrane (EMD Millipore, IPFL07810), blocked for 1 hour in 2% (v/v) BSA (Bioworld, 40220068-1) in TBST (tris-buffered saline with 0.3% Tween 20), and probed overnight with primary antibodies diluted in the above blocking buffer. Following extensive washes, HRP-linked enhanced chemiluminescence (ECL)–conjugated secondary antibodies, goat anti-rabbit IgG and rabbit anti-mouse IgG, were used to identify proteins of interest and were visualized with a SuperSignal West Pico PLUS ECL substrate (Thermo Fisher Scientific, 34579).

For in vitro binding assays with GST-Rho1, in vitro translated RhoGEF2 PH domains were detected on immunoblots with streptavidin-HRP (1:5000; Thermo Fisher Scientific, SA10001) and visualized with a SuperSignal West Pico PLUS ECL substrate (Thermo Fisher Scientific, 34579). The membranes were subsequently incubated with hydrogen peroxide to quench the bound HRP, washed, and subjected to GST immunoblotting using the above protocol.

### Statistics

All experiments were performed with at least two independent replicates, and the number of embryos/cells analyzed is noted in the figures. Imaging data were excluded if cells moved out of the focal plane or if signal intensity became substantially reduced due to photobleaching. All statistical comparisons were carried out in Prism (GraphPad) using Mann-Whitney tests for pairwise comparisons, as most data did not fulfill assumptions of normality.

## Supplementary Materials

### This PDF file includes:

Figs. S1 to S13 Tables S1 and S2 References

## Notes

View/request a protocol for this paper from *Bio-protocol*.
