# Supplementary material for: An AMPK phosphoregulated RhoGEF feedback loop tunes cortical flow–driven amoeboid migration in vivo
Source: Sci Adv. 2022 Sep 14;8(37):eabo0323. doi: 10.1126/sciadv.abo0323 (PMC9473612; doi:10.1126/sciadv.abo0323)
Supplement: Supplementary file 1 — Figs. S1 to S13 Tables S1 and S2 References [file sciadv.abo0323_sm.pdf]

Science Advances
AAAS

Supplementary Materials for

An AMPK phosphoregulated RhoGEF feedback loop tunes cortical flow–driven amoeboid migration in vivo

Benjamin Lin *et al.*

Corresponding author: Ruth Lehmann, lehmann@wi.mit.edu; Benjamin Lin, benjamin.lin@nyulangone.org

Sci. Adv. **8**, eabo0323 (2022)
DOI: 10.1126/sciadv.abo0323

The PDF file includes:

Figs. S1 to S13
Tables S1 and S2
Legends for movies S1 to S26
References

Other Supplementary Material for this manuscript includes the following:

Movies S1 to S26

Fig. S1- Clarification of myosin II flow and phenotypes from constitutively active RhoA overexpression

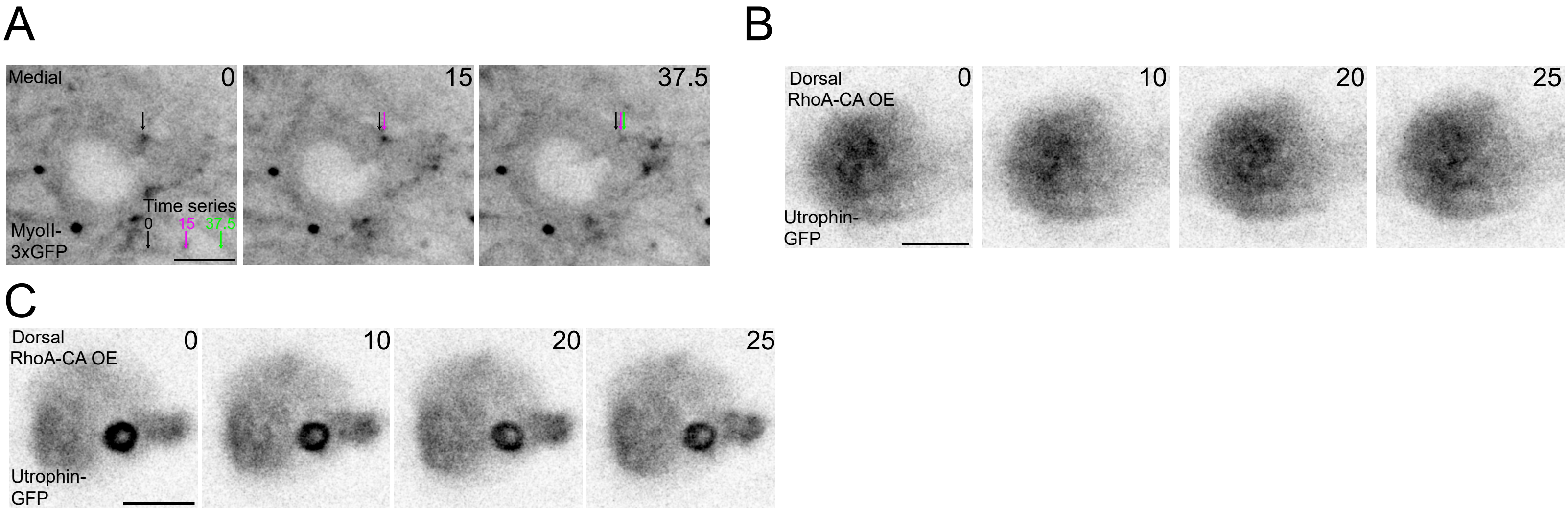

(**A**) Timelapse images from a single Z medial plane of the representative PGC expressing myoII-3xGFP shown in Fig. 1D. The different colored arrows track the retrograde movement of the myosin II foci at the indicated time points. (**B-C**) Two photon time-lapse imaging of the dorsal plane of a representative PGC expressing Utrophin-GFP and RhoA-constitutively active (CA). PGCs have a bright homogeneous cortex in 41/41 cells imaged while 11/41 cells additionally have a bright ring structure (**C**). Times are in seconds. Scale bars, 5 μm.

Fig. S2- PGC dynamics *in vitro*

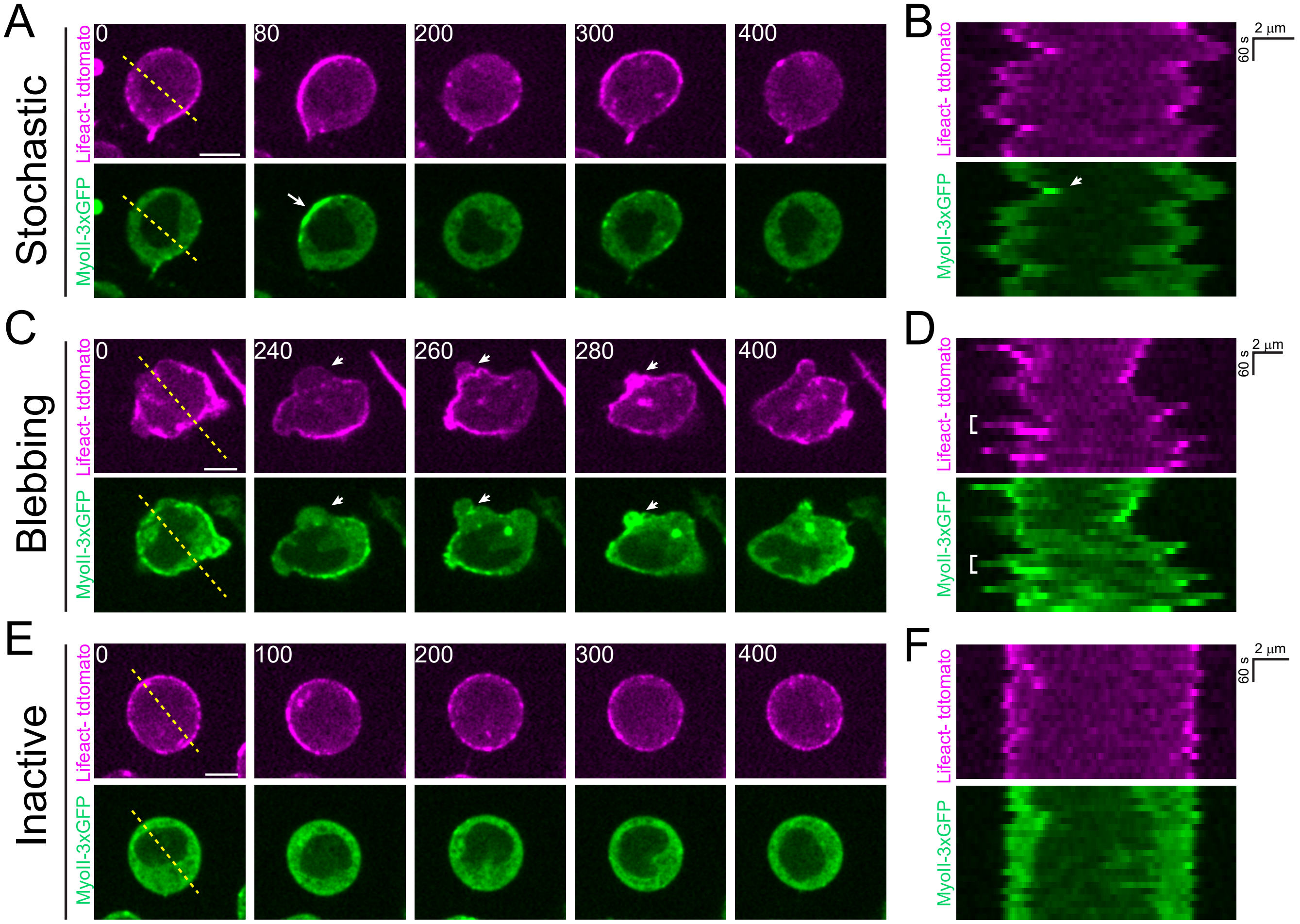

(**A-F**) Timelapse imaging of representative extracted PGCs expressing lifeact-tdTomato and myosin II-3xGFP in medium without serum on coverslips which are stochastic (**A-B**), blebbing (**C-D**), or inactive (**E-F**). Yellow dotted lines indicate where the kymographs in **B**,**D**,**F** were taken. White arrow in **A** indicates a region where myosin II has transiently accumulated. The equivalent region in the kymograph in **B** is also indicated with a white arrow. White arrows in **C** highlight a bleb and its subsequent retraction. Bleb emergence and retraction are highlighted by a white bracket in **D**. Scale bars, 5 µm. Times are in seconds.

Fig. S3- Additional cortical flow analysis and control images

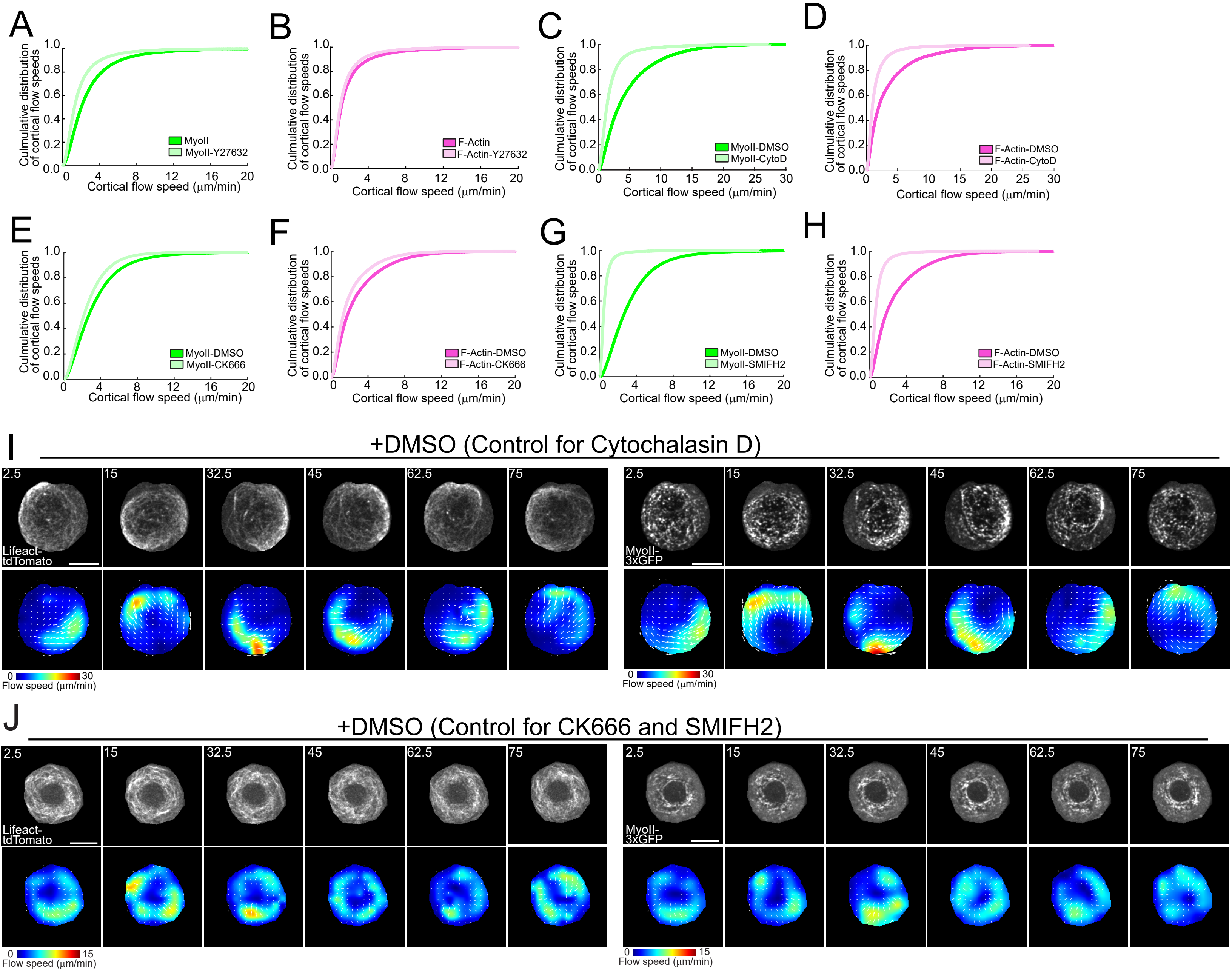

(**A-H**) Culmulative distribution of cortical flow speeds of myosin II (**A,C,E,G**) and F-actin (**B,D,F,H**) after the indicated drug treatments. (**I-J**) Top- time-lapse imaging of representative extracted PGCs under agarose expressing lifeact-tdTomato (left panels) and myosin II-3xGFP (right panels) treated with DMSO. Bottom- PIV flow analysis between consecutive images. Flow speed is color coded with the indicated color bar. White arrows are flow vectors scaled to flow magnitude. Scale bar, 10 μm. All times are in seconds.

Fig. S4- RhoGEF2 localization under endogenous regulation in PGCs

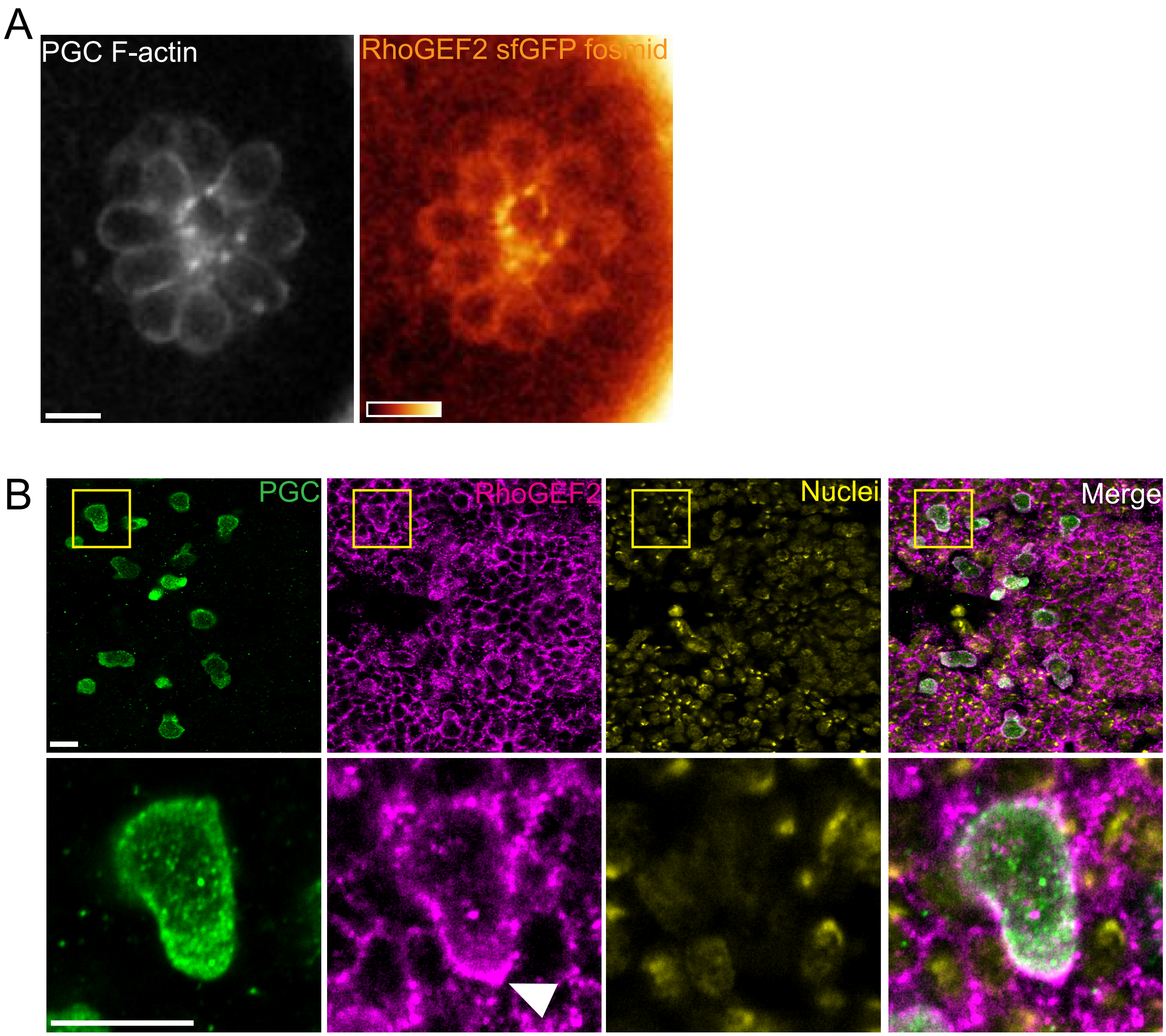

(**A**) Representative two photon image from a central plane in a PGC cluster expressing lifeact-tdTomato, tdKatushka2-CAAX (not shown), and a RhoGEF2-sfGFP fosmid. The image is representative of 9 embryos imaged. The RhoGEF2 sfGFP fosmid is color coded with the indicated color bar. (**B**) Representative immunofluorescent image from a stage 11 embryo, where PGCs are migrating towards the mesoderm (top and bottom of this image). The yellow box in top images show the enlarged image below. The white arrow indicates where RhoGEF2 has accumulated at the rear of the PGC. This image is representative of 5 embryos imaged. Scale bars, 10 μm in all images.

Fig. S5- Characterization of the relationship between RhoGEF2 and Tre1 along with validation of degradation with degradFP

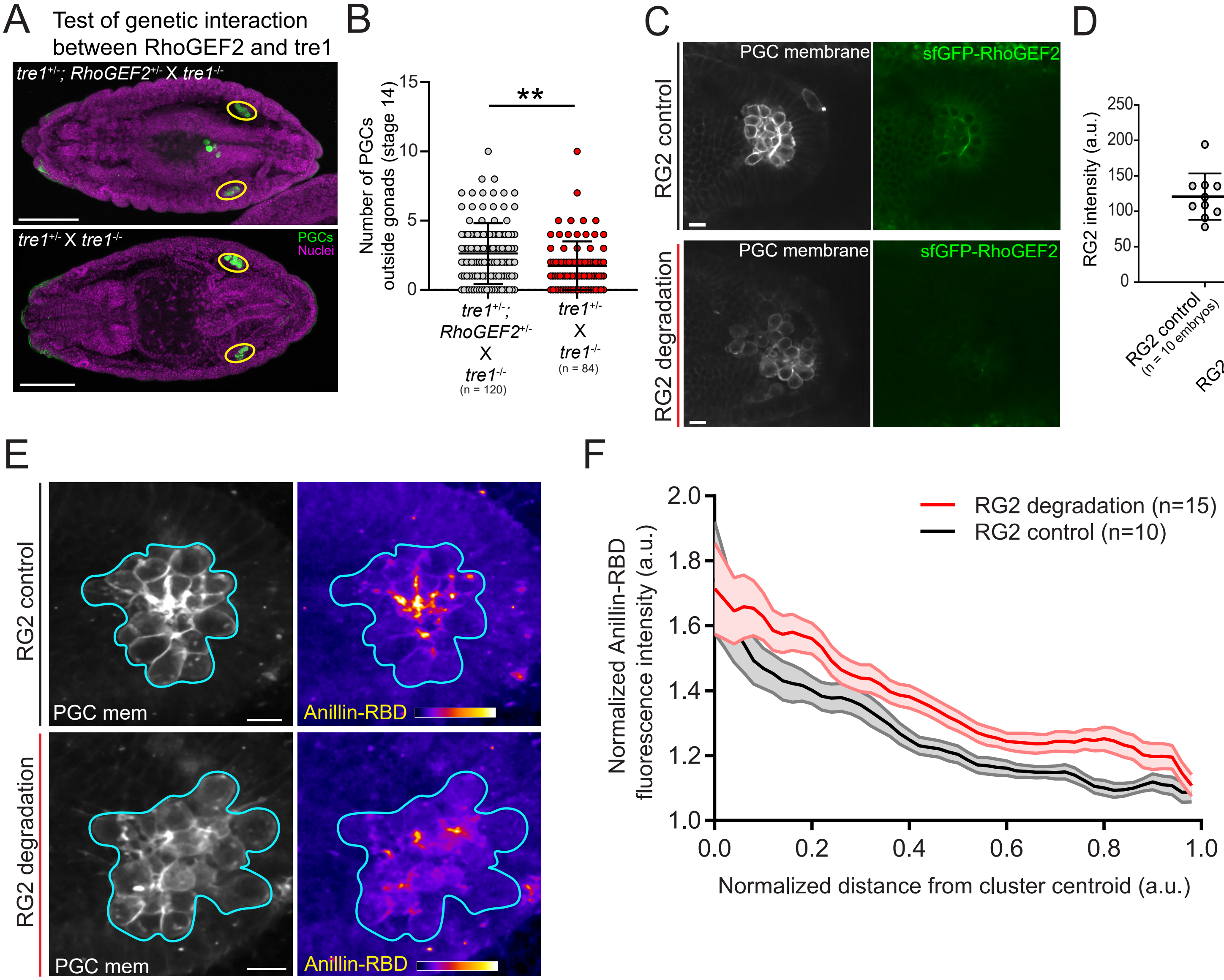

(**A**) Representative immunofluorescence images of stage 14 embryos with the indicated genotypes. The maternal genotype is listed first folllowed by the paternal genotype it was crossed to. Yellow ovals indicate the gonads. Scale bars, 100 μm. (**B**) Quantification of the number of PGCs outside gonads with the genotypes described below. The number of embryos analyzed is indicated. Error bars are SD. (**C)** Representative two photon image from a central plane in a PGC cluster expressing tdkatushka2-CAAX (PGC membrane marker) and sfGFP-RhoGEF2 under control (top) and RhoGEF2 degradation (bottom) conditions. The image is representative of n=10 embryos (RG2 control) and n=14 embryos (RG2 degradation). RhoGEF2, RG2. Scale bars, 10 μm. (**D**) Quantification of mean RhoGEF2 intensity in the central plane of PGC clusters under the indicated conditions. n = number of embryos imaged. (**E**) Representative two photon image from a central plane in a PGC cluster expressing tdkatushka2-CAAX (PGC membrane marker) and Anillin-RhoA-GTP binding domain (RBD)-tdTomato under control (top) and RhoGEF2 degradation (bottom) conditions. Cyan lines trace the boundary of the cluster. The image is representative of n = 10 embryos (RG2 control) and n = 15 embryos (RG2 degradation). Anillin-RBD-tdTomato is pseudocolored with the color bar shown below and both images are scaled to the same intensity range. Scale bars, 10 μm (**F**) Quantification of Anillin-RBD-tdTomato intensity as a function of distance from the cluster centroid. n = number of embryos analyzed. Error bars are SEM. Statistical comparisions are pairwise from a Mann-Whitney test in **B**. **=p<.01.

Fig. S6- Expression of constitutively active G$\alpha_{12/13}$ enhances active RhoA polarity in PGC clusters

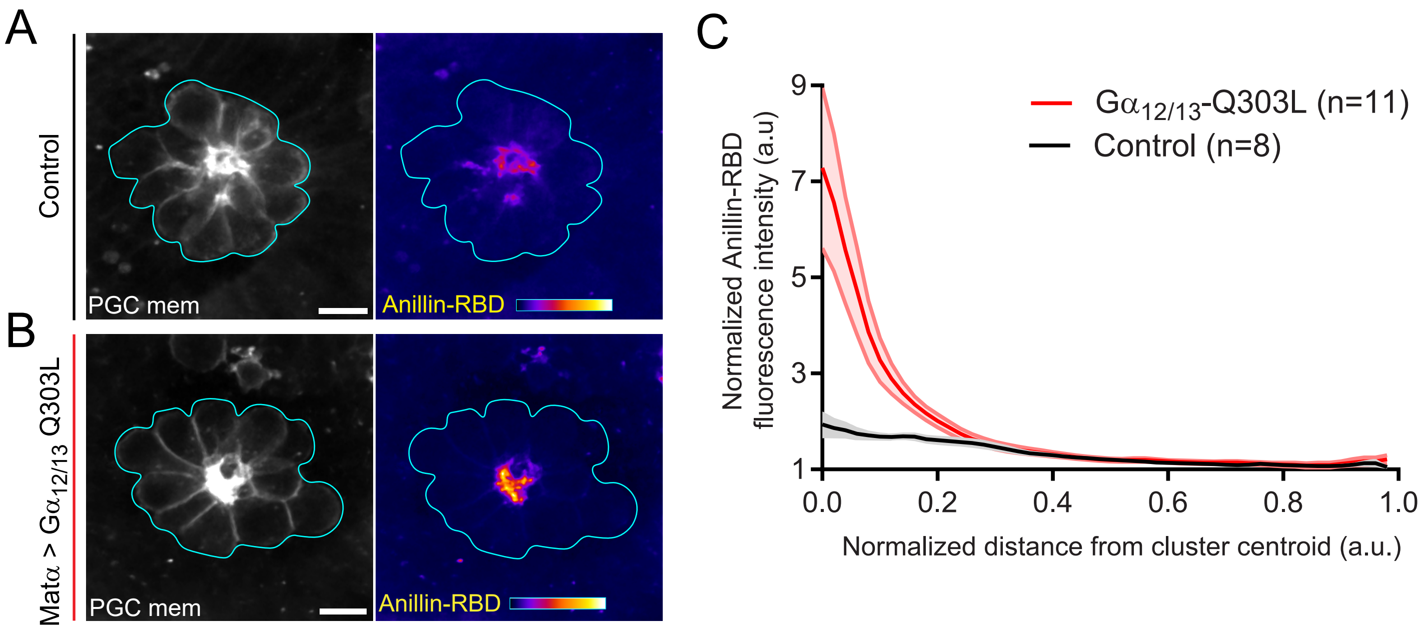

(**A-B)** Representative two photon image from a central plane in a PGC cluster expressing Anillin-RBD-tdTomato and tdkatushka2-CAAX under the indicated conditions. The image is representative of n=8 embryos (control) and n=11 embryos (G$\alpha_{12/13}$-Q303L). The color bar used for the Anillin-RBD image is shown below and both images are scaled to the same intensity range. Scale bars, 10 μm. (**C**) Quantification of Anillin-RBD-tdTomato intensity as a function of distance from the cluster centroid. n = number of embryos analyzed. Error bars are SEM.

Fig. S7- Characterization of RhoGEF2 domain truncation constructs

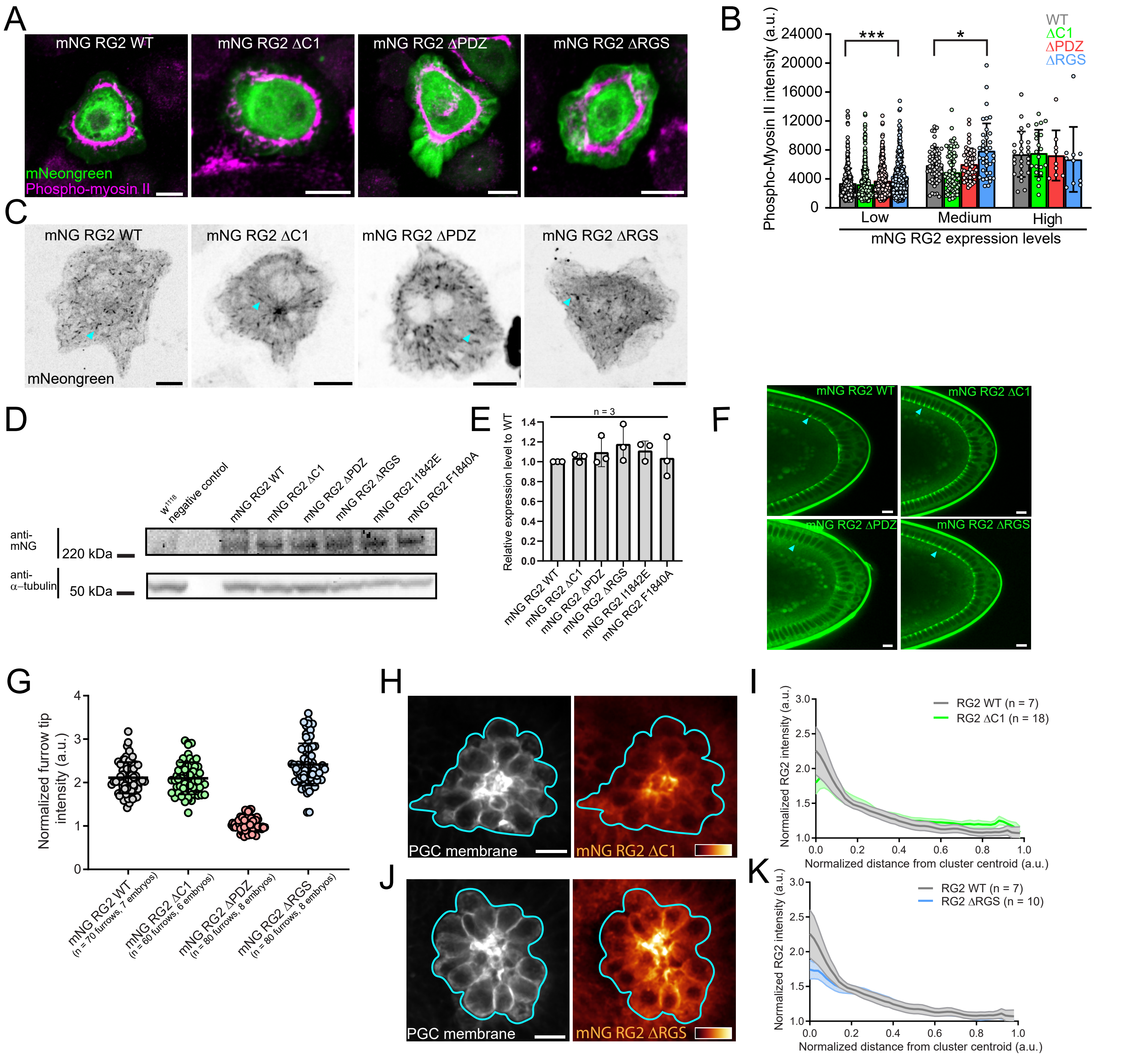

(**A**) Representative immunofluorescence images from insect S2 cells overexpressing the indicated mNeongreen RhoGEF2 construct and co-stained with a phospho-myosin II antibody. Note that microtubule plus-end tracking is abolished under the utilized fixation conditions. (**B**) Quantification of phospho-myosin II intensity as a function of mNeongreen RhoGEF2 construct expression level. Expression levels were split into 3 equally sized bins. The number of cells analyzed from 3 independent experiments is- WT low- 317, WT medium- 50, and WT high- 26, ΔC1 low- 517, ΔC1 medium- 74, and ΔC1 high- 20. ΔPDZ low- 410, ΔPDZ medium- 47, and ΔPDZ high- 9. ΔRGS low- 259, ΔRGS medium- 38, and ΔRGS high- 10. (**C**) Representative image from live S2 cells expressing the indicated mNeongreen RhoGEF2 construct. The cyan arrows indicate that microtubule plus-end tracking is intact in all constructs. (**D**) Representative immunoblot from embryonic lysates expressing the indicated mNeongreen RhoGEF2 transgene probed with the indicated antibodies. w^{1118} is a negative control for antibody specificity. (**E**) Quantification of mNeongreen RhoGEF2 transgene expression levels relative to WT. n = number of independent replicates. (**F**) Representative two photon image from the posterior pole of stage 5 embryos expressing the indicated mNeongreen RhoGEF2 transgene. Cyan arrows inicate mNeongreen RhoGEF2 transgene enrichment on furrow tips, with the exception of the ΔPDZ transgene. (**G**) Quantification of furrow tip enrichment of different mNeongreen RhoGEF2 transgenes. The number of furrows and embryos analyzed are indicated. Error bars are SD. (**H,J**) Representative two photon image from central plane of PGC clusters expressing tdKatushka2 CAAX (PGC membrane marker) and the indicated mNeongreen RhoGEF2 transgene. mNeongreen RhoGEF2 is pseudocolored with the color bar in the image and scaled to the same intensity range. (**I,K**) Quantification of mNeongreen RhoGEF2 transgene intensity as a function of distance from the cluster centroid. n = number of embryos analyzed. Error bars are SEM. Scale bars, 10 μm in all images. Statistical comparisons are pairwise from a Mann-Whitney test in **B.** *=p<.05, *** = p<.001.

Fig. S8- Characterization of RhoGEF2 PH mutants

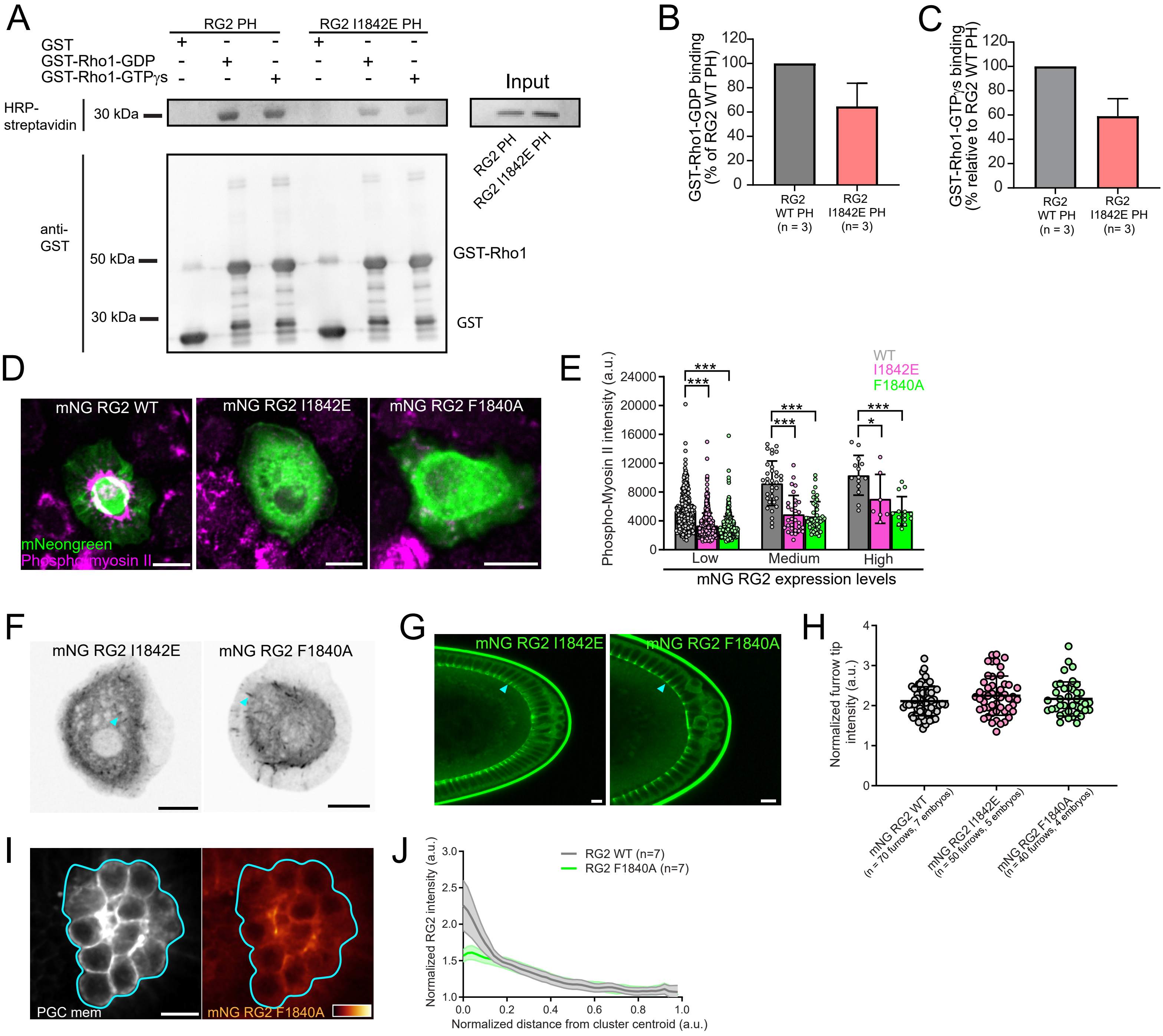

(**A**) Representative immunoblot from an in vitro binding assay between the in vitro translated, biotin conjugated, RhoGEF2 PH domains and purified GST fusion proteins. The top image is probed with HRP-streptavidin to identify the amount of biotin-conjugated RhoGEF2 PH domain pulled down with each GST protein. The same membrane was then stripped and stained with anti-GST antibody to determine the amount of GST protein present in each sample (bottom). (**B-C**) Quantification of the amount of RhoGEF2 PH domain bound to GST-Rho1-GDP (**B**) or GST-Rho1-GTPγs (**C**) relative to the WT PH domain. (**D**) Representative immunofluorescence images from insect S2 cells overexpressing the indicated mNeongreen RhoGEF2 construct and co-stained with a phospho-myosin II antibody. Note that microtubule plus-end tracking is abolished under the utilized fixation conditions. (**E**) Quantification of phospho-myosin II intensity as a function of mNeongreen RhoGEF2 construct expression level. Expression levels were split into 3 equally sized bins. The number of cells analyzed from 3 independent experiments is- WT low- 403, WT medium- 34, and WT high- 14, 1842E low- 573, 1842E medium- 30, and 1842E high- 7. F1840A low- 501, F1840A medium- 47, and F1840A high- 14. (**F**) Representative image from live S2 cells expressing the indicated mNeongreen RhoGEF2 construct. Cyan arrows indicate that microtubule plus-end tracking is intact in all constructs. (**G**) Representative two photon image from the posterior pole of stage 5 embryos expressing the indicated mNeongreen RhoGEF2 transgene. Cyan arrows note mNeongreen RhoGEF2 transgene enrichment on furrow tips. (**H**) Quantification of furrow tip enrichment of different RhoGEF2 transgenes. The number of furrows and embryos analyzed are indicated. Error bars are SD. (**I**) Representative two photon image from central plane of PGC cluster expressing tdKatushka2-CAAX (PGC membrane marker) and the noted mNeongreen RhoGEF2 transgene. mNeongreen RhoGEF2 is pseudocolored with the color bar in the image. (**J**) Quantification of mNeongreen RhoGEF2 transgene intensity as a function of distance from the cluster centroid compared to WT. n = number of embryos analyzed. Error bars are SEM. Scale bars, 10 μm in all images. Statistical comparisons are pairwise from a Mann-Whitney test in **E.** *=$p<.05$, *** = $p<.001$.

Fig. S9- Cortical flow analysis of RhoGEF2 PDZ domain truncation and PH mutant

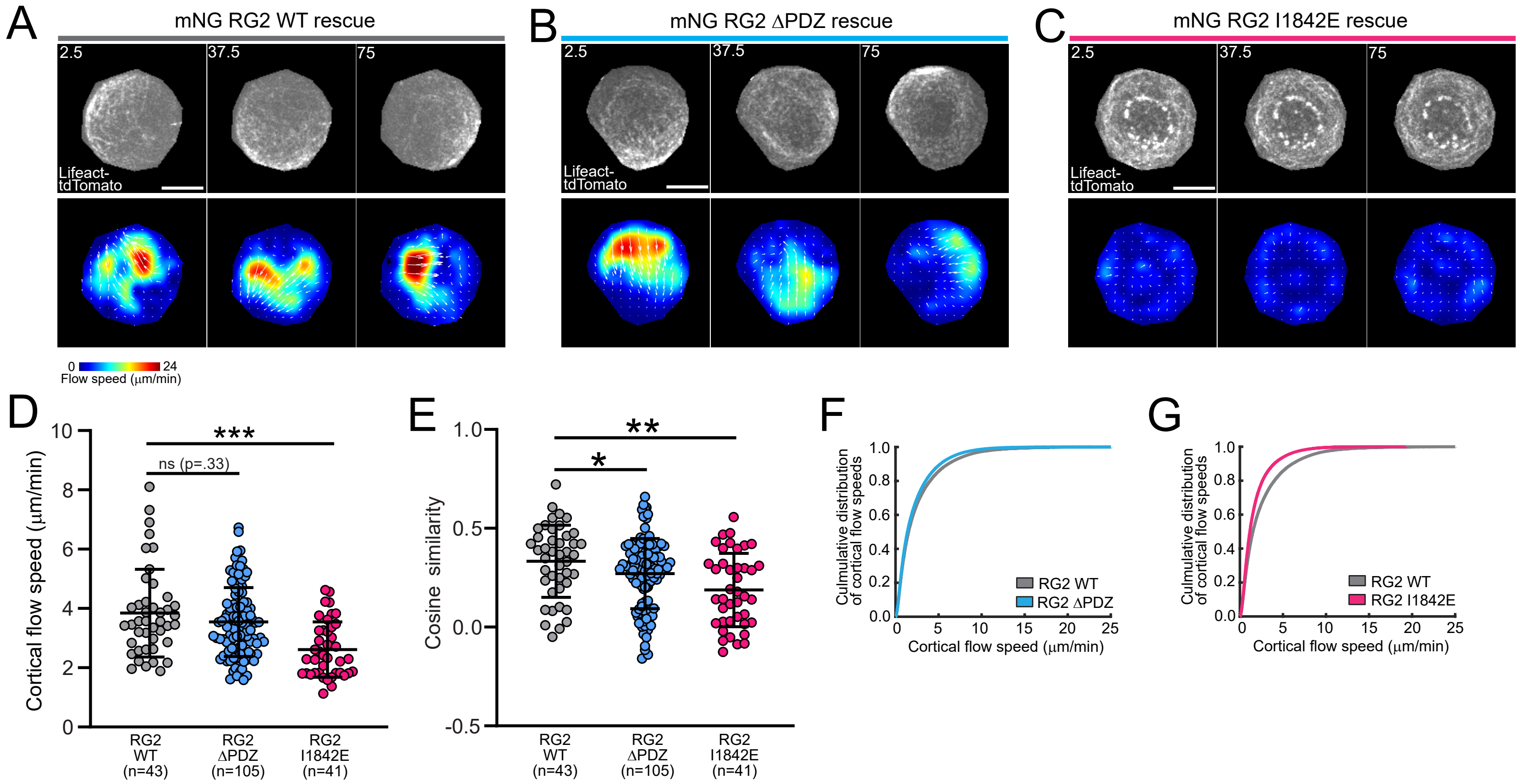

(**A-C**) Top- time-lapse imaging of representative extracted PGCs under agarose expressing lifeact-tdTomato and rescued with the labeled mNeongreen RhoGEF2 constructs (PGCs co-express sfGFP-RhoGEF2 and a given mNeongreen RhoGEF2 construct. sfGFP-RhoGEF2 is degraded with the degradFP system, leaving the PGCs with solely the mNeongreen RhoGEF2 construct). Bottom- PIV flow analysis between consecutive images. Flow speed is color coded with the indicated color bar. White arrows are flow vectors scaled to flow magnitude. Scale bar, 10 μm. All times are in seconds. (**D-E**) Quantification of mean cortical actin flow speed (**D**) and mean cosine similarity between vectors over consecutive time points (**E**) under the labeled rescue conditions. n = number of cells analyzed. Error bars are SD. (**F-G**) Culmulative distribution of cortical flow speeds under the indicated rescue conditions. Statistical comparisons are pairwise from a Mann-Whitney test in **D-E.** *=p<.05, **=p<.01, ***=p<.001.

Fig. S10- Microtubule dynamics in PGCs and relationship with RhoGEF2

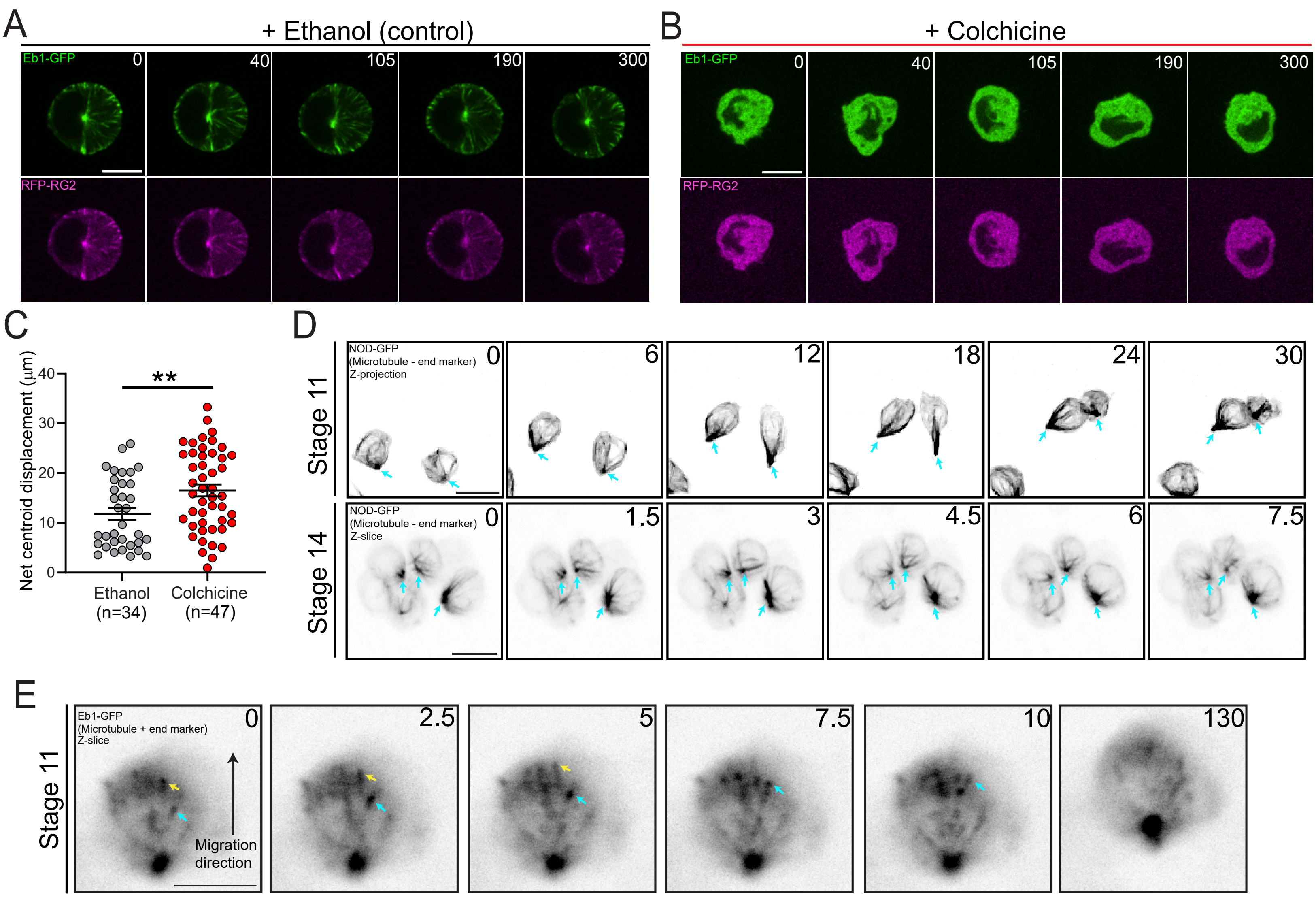

(**A-B**) Representative time-lapse imaging of extracted PGCs on a coverslip co-expressing EB1-GFP (top) and RFP-RhoGEF2 (bottom) treated with ethanol (control) (**A**) or colchicine (**B**). Times are in seconds. (**C**) Quantification of net PGC centroid displacement under the indicated experimental conditions. n = the number of cells analyzed. (**D**) Time-lapse imaging of representative PGCs expressing NOD-GFP migrating toward the mesoderm (stage 11, top, Z-projection) or arresting within the primitive gonad (stage 14, bottom, Z-slice). Cyan arrows mark areas where NOD-GFP accumulates at the posterior of PGCs and where microtubules emanate from. Images are representative of n = 6 embryos (Stage 11) and n = 6 embryos (Stage 14). Times are in minutes. (**E**) Time-lapse imaging of a representative PGC expressing EB1-GFP migrating toward the mesoderm (stage 11). Images are representative of n = 12 PGCs imaged from n = 7 embryos. Yellow and cyan arrows track individual EB1 comets over time as they travel across the PGC from back to front. The direction of migration is noted with a black arrow. The large, bright EB1 foci at the rear of the cell is a centrosome. Times are in seconds. All scale bars, 10 μm. Statistical comparisons are pairwise from a Mann-Whitney test in **C.** **=p<.01.

Fig. S11- Characterization of RhoGEF2 SKIP mutants

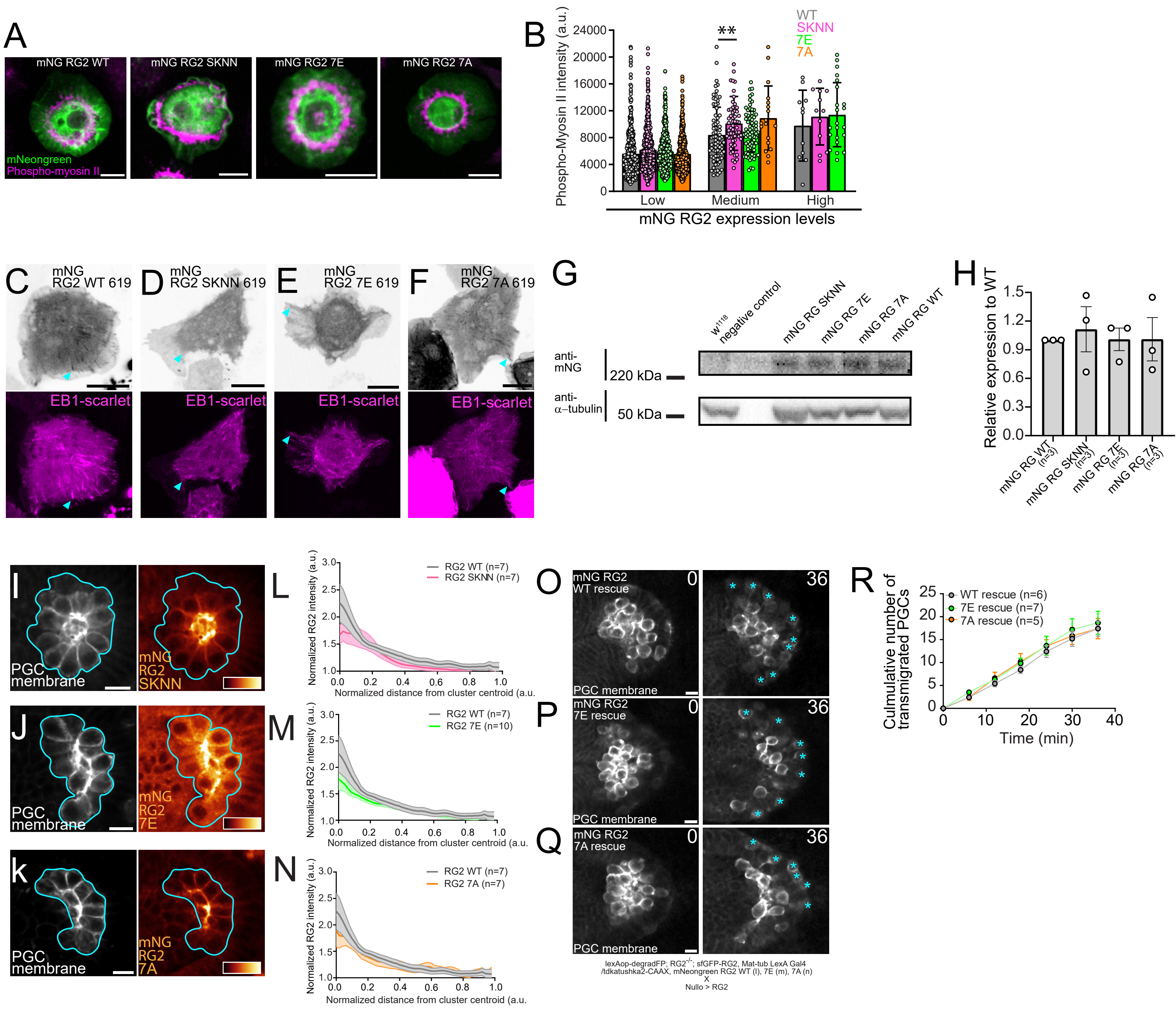

(**A**) Representative immunofluorescence images from insect S2 cells overexpressing the indicated mNeongreen RhoGEF2 construct and co-stained with a phospho-myosin II antibody. Note that microtubule plus-end tracking is abolished under the utilized fixation conditions. (**B**) Quantification of phospho-myosin II intensity as a function of mNeongreen RhoGEF2 construct expression level. Expression levels were split into 3 equally sized bins. The number of cells analyzed from 3 independent experiments is- WT low- 474, WT medium- 76, and WT high- 11, SKNN low- 573, SKNN medium- 59, and SKNN high- 12. 7E low- 450, 7E medium- 74, and 7E high- 22. 7A low- 606 and 7A medium- 18. We did not observe any high expressing mNeongreen RhoGEF2 7A S2 cells. Error bars are SD. (**C-F**) Representative images from live S2 cells co-expressing a given truncated mNeongreen RhoGEF2 construct (AA 1-619) and EB1-mScarlet. Cyan arrows indicate that microtubule plus-end tracking is maintained in **C,F** but abolished in **D,E**. (**G**) Representative immunoblot from embryonic lysates expressing the labeled mNeongreen RhoGEF2 transgenes stained with the indicated antibodies. w^{1118} is a negative control for antibody specificity. (**H**) Quantification of mNeongreen RhoGEF2 transgene expression levels relative to WT. n = number of independent replicates. Error bars are SEM. (**I-K**) Representative two photon image from central plane of a PGC cluster expressing tdKatushka2-CAAX (PGC membrane marker) and the indicated mNeongreen RhoGEF2 transgene. mNeongreen RhoGEF2 is pseudocolored with the color bar in the image and scaled to the same intensity range. Cyan outlines the PGC cluster. (**L-N**) Quantification of mNeongreen RhoGEF2 transgene intensity as a function of distance from the cluster centroid. n = number of embryos analyzed. Error bars are SEM. (**O-Q**) Representative two photon time-lapse imaging of PGC clusters expressing tdKatushka2-CAAX (PGC membrane marker) dispersing under the indicated rescue conditions. Genotypes are indicated below. Cyan asterisks mark transmigrated PGCs. Times are in minutes. (**R**) Quantification of the total number of transmigrated PGCs over time under the indicated resuce conditions. n = number of embryos analyzed. Error bars are SEM. Scale bars, 10 μm in all images. Statistical comparisons are pairwise from a Mann-Whitney test in **B**. ** = p<.01.

Fig. S12- Cortical flow analysis of RhoGEF2 7E mutant

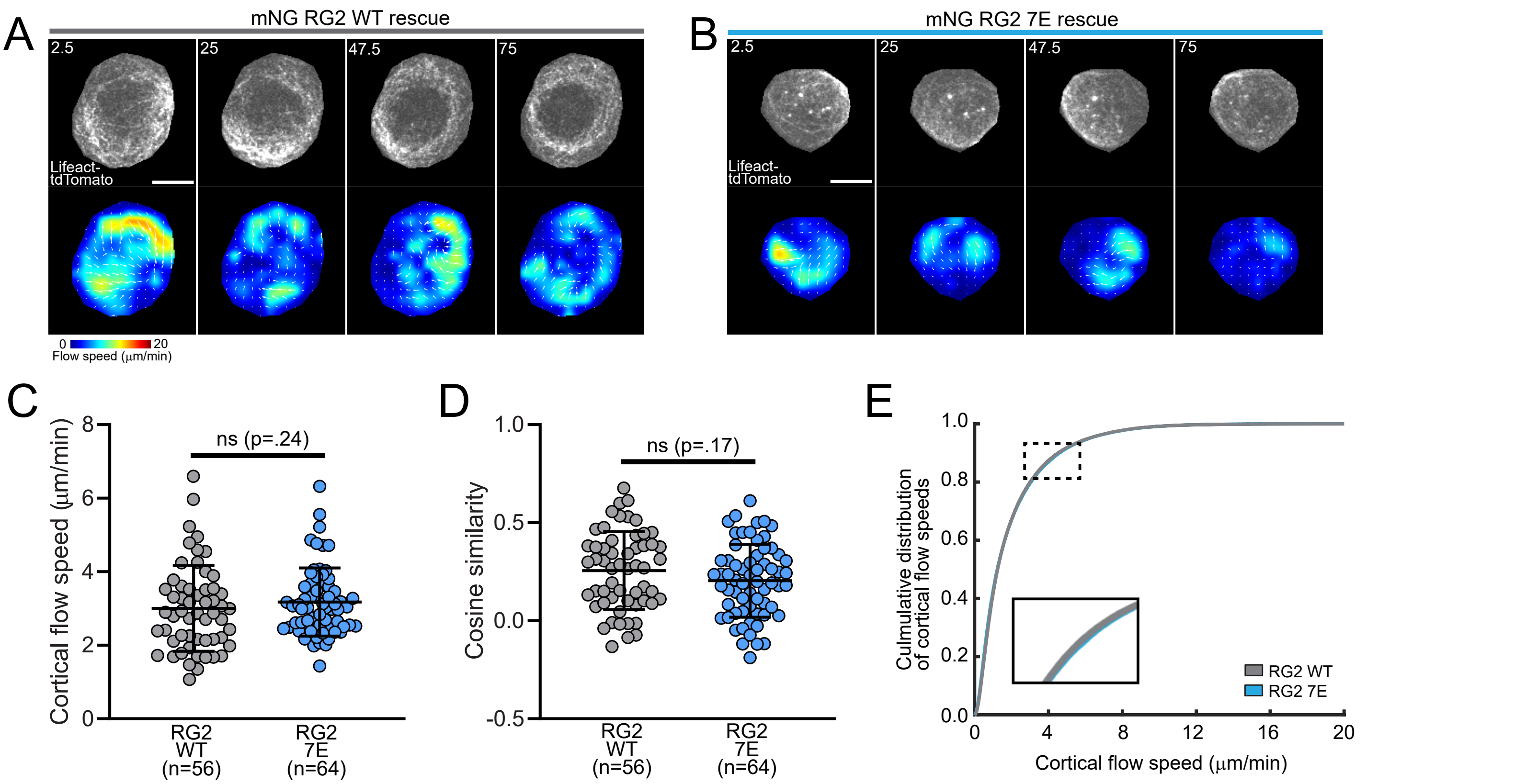

(**A-B**) Top- time-lapse imaging of representative extracted PGCs under agarose expressing lifeact-tdTomato and rescued with the labeled mNeongreen RhoGEF2 constructs (PGCs co-express sfGFP-RhoGEF2 and a given mNeongreen RhoGEF2 construct. sfGFP-RhoGEF2 is degraded with the degradFP system, leaving the PGCs with solely the mNeongreen RhoGEF2 construct). Bottom- PIV flow analysis between consecutive images. Flow speed is color coded with the indicated color bar. White arrows are flow vectors scaled to flow magnitude. Scale bar, 10 μm. All times are in seconds. (**C-D**) Quantification of mean cortical actin flow speed (**C**) and mean cosine similarity between vectors over consecutive time points (**D**) under the labeled rescue conditions. n = number of cells analyzed. Error bars are SD. (**E**) Culmulative distribution of cortical flow speeds under the indicated rescue conditions. The black dotted box is magnified in the inset to show the overlap between the two plotted distributions. Statistical comparisons are pairwise from a Mann-Whitney test in **C-D.**

Fig. S13- Representative images of PGCs with and without pharmacological AMPK activation

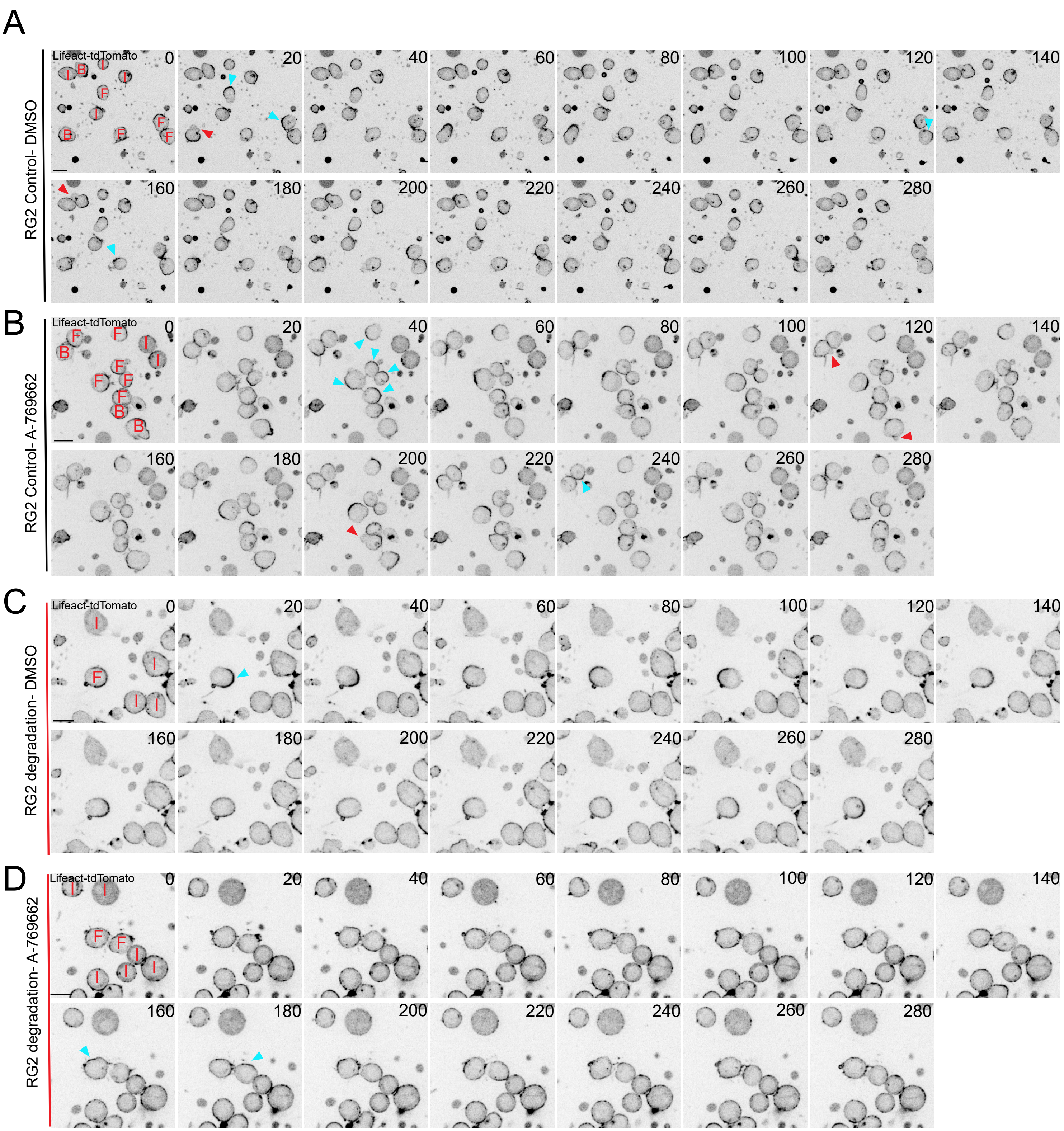

(**A-D**) Time-lapse imaging of representative extracted PGCs on a coverslip expressing lifeact-tdTomato under RhoGEF2-control (**A-B**) and degradation (**C-D**) conditions with (**B,D**) and without (**A,C**) pharmacological AMPK activation with A-769662. The initial timepoint in each image series is labeled with red letters corresponding to different classes of behaviors (B- blebbing, F- flow, I- inactive) observed during the experiment. Cyan arrows mark regions where F-actin accumulates in a clear crescent during circular cortical flow. The circular movement(s) of these F-actin crescents are best appreciated in Supplemental Movies 25 (**A-B**) and 26 (**C-D**). Red arrows mark instances of blebs defined as cellular projections devoid of cortical F-actin. Only one crescent or bleb is marked for each cell demonstrating cortical flow or blebbing, respectively, for clarity. Scale bars, 10 μm. All times are in seconds.

**Table S1-** mNeongreen RhoGEF2 transgene rescue				
**mNeongreen RhoGEF2 transgene rescue**	RhoGEF2^{04291}/DF(2R)BSC331 (RhoGEF2 homozygous mutant, females rescued)	RhoGEF2^{04291}/Cyo or DF(2R)BSC331/Cyo (RhoGEF2 heterozygous mutant, females)	RhoGEF2^{04291}/DF(2R)BSC331 (RhoGEF2 homozygous mutant, males rescued)	RhoGEF2^{04291}/Cyo or DF(2R)BSC331/Cyo (RhoGEF2 heterozygous mutant, males)
RhoGEF2^{04291}/Cyo<br>X<br>DF(2R)BSC331/Cyo	0	132	0	149
RhoGEF2^{04291}/Cyo<br>X<br>DF(2R)BSC331/Cyo; **squash> mNG RhoGEF2 WT**/TM3,Ser	48	94	51	97
RhoGEF2^{04291}/Cyo<br>X<br>DF(2R)BSC331/Cyo; **squash> mNG RhoGEF2 ΔC1**/TM3,Ser	49	89	54	119
RhoGEF2^{04291}/Cyo<br>X<br>DF(2R)BSC331/Cyo; **squash> mNG RhoGEF2 ΔPDZ**/TM3,Ser	3	104	1	76
RhoGEF2^{04291}/Cyo<br>X<br>DF(2R)BSC331/Cyo; **squash> mNG RhoGEF2 ΔRGS**/TM3,Ser	2	82	2	65
RhoGEF2^{04291}/Cyo<br>X<br>DF(2R)BSC331/Cyo; **squash> mNG RhoGEF2 1842E**/TM3,Ser	0	115	0	136
RhoGEF2^{04291}/Cyo<br>X<br>DF(2R)BSC331/Cyo; **squash> mNG RhoGEF2 F1840A**/TM3,Ser	0	64	0	85

Quantification of genotypes emerging from crosses shown in the first column to assess if a given mNeongreen RhoGEF2 transgene can rescue RhoGEF2 mutants. Only the mNeongreen RhoGEF2 WT and ΔC1 transgenes can rescue RhoGEF2 mutants, as the ΔPDZ and ΔRGS transgenes provide extremely rare rescue.

Table S2- Experimental genotypes

Figure	Genotype	Source
1B,D	nos-Lifeact-tdTomato-P2A-tdKatushka2-CAAX; MyoII::3XGFP	nos-Lifeact-tdTomato-P2A-tdKatushka2-CAAX- **Ruth Lehmann** (*24*)<br>MyoII::3xGFP- **Yohanns Bellaïche** (*70*)
1F (left)	P{w[+mC]=GAL4::VP16-nos.UTR}CG6325[MVD1] (female)<br>X<br>P{UASp-hUTRN.ABD.EGFP} , P{w[+mC]=UAS-Rac1.L}3 (male)	P{UASp-hUTRN.ABD.EGFP} - **Thomas Lecuit** (*71*)**, provided by Tina Tootle**<br>P{w[+mC]=UAS-Rac1.L}3- **BL6293**
1F (right)	P{w[+mC]=GAL4::VP16-nos.UTR}CG6325[MVD1] (female)<br>X<br>P{UASp-hUTRN.ABD.EGFP} ; P{UAS-Rac1.V12}1 (male)	P{UAS-Rac1.V12}1- **BL6291**
1H (left)	P{w[+mC]=GAL4::VP16-nos.UTR}CG6325[MVD1] (female)<br>X<br>P{UASp-hUTRN.ABD.EGFP} (male)	
1H (right)	P{w[+mC]=GAL4::VP16-nos.UTR}CG6325[MVD1] (female)<br>X<br>P{UASp-hUTRN.ABD.EGFP} , P{w[+mC]=UAS-Rho1.N19}2 (male)	P{w[+mC]=UAS-Rho1.N19}2- **BL58818**
2A-B, C-D, E-F, I-J	nos-Lifeact-tdTomato-P2A-tdKatushka2-CAAX; MyoII::3XGFP	
3A, E-F	nos-Lifeact-tdTomato-P2A-tdKatushka2-CAAX/+; P{sqh-sfGFP-RhoGEF2}30/+	P{sqh-sfGFP-RhoGEF2}30- **BL76260**
3B	tre1$^{ep\Delta5}$; nos-Lifeact-tdTomato-P2A-tdKatushka2-CAAX/+; P{sqh-sfGFP-RhoGEF2}30/+	tre1$^{ep\Delta5}$- **Ruth Lehmann** (*25*)
4A, D, G, J	**RG2 control**- cn^{1} P{PZ}RhoGEF2^{04291}/Df(2R)BSC331;<br>LexAop-degradFP-*nos* TCE-*pgc* 3'UTR, P{sqh-sfGFP-RhoGEF2}30/ nos-Lifeact-tdTomato-P2A-tdKatushka2-CAAX (female)<br>X<br>P{nullo-GAL4.G}5.20/+; P{w[+mC]=UASp-T7.RhoGEF2}5 (male)	cn^{1} P{PZ}RhoGEF2^{04291}- **BL11369**<br>Df(2R)BSC331- **BL24356**<br>LexAop-degradFP-nosTCE-pgc 3'UTR- **This study**<br>P{nullo-GAL4.G}5.20- **BL26875**<br>P{w[+mC]=UASp-T7.RhoGEF2}5- **BL9386**
4B, E, H, K	**RG2 degradation**- cn^{1} P{PZ}RhoGEF2^{04291}/Df(2R)BSC331;<br>LexAop-degradFP-*nos* TCE-*pgc* 3'UTR, P{sqh-sfGFP-RhoGEF2}30/ nos-Lifeact-tdTomato-P2A-tdKatushka2-CAAX, MatTub-LexA-GAD (female)<br>X<br>P{nullo-GAL4.G}5.20/+; P{w[+mC]=UASp-T7.RhoGEF2}5 (male)	MatTub-LexA-GAD- **This study**
5B, E (top), H (top), J	**Control**- P{matα4-GAL-VP16}67, nos-myosin II-tdTomato-P2A-tdKatushka2-CAAX/+ ;<br>P{matα4-GAL-VP16}15, nos-myosin II-tdTomato-P2A-tdKatushka2-CAAX/+	P{matα4-GAL-VP16}67, P{matα4-GAL-VP16}15- **BL80361**<br>nos-myosin II-tdTomato-P2A-tdKatushka2-CAAX- **This study**
5C, E (bottom),<br>H (bottom), K	**Matα > Gα$_{12/13}$-Q303L**- P{matα4-GAL-VP16}67, nos-myosin II-tdTomato-P2A-tdKatushka2-CAAX/UASp-Gα$_{12/13}$-Q303L-*nos* TCE-*pgc* 3'UTR ;<br>P{matα4-GAL-VP16}15, nos-myosin II-tdTomato-P2A-tdKatushka2-CAAX/+	UASp-Gα$_{12/13}$-Q303L-*nos* TCE-*pgc* 3'UTR- **This study**
6C	nos-Lifeact-tdTomato-P2A-tdKatushka2-CAAX/+; sqh-mNeongreen-RhoGEF2/+	sqh-mNeongreen-RhoGEF2- **This study**
6D	nos-Lifeact-tdTomato-P2A-tdKatushka2-CAAX/+; sqh-mNeongreen-RhoGEF2 ΔPDZ/+	sqh-mNeongreen-RhoGEF2 ΔPDZ- **This study**
6E	nos-Lifeact-tdTomato-P2A-tdKatushka2-CAAX/+; sqh-mNeongreen-RhoGEF2 I1842E/+	sqh-mNeongreen-RhoGEF2 I1842E- **This study**
6F	nos-Lifeact-tdTomato-P2A-tdKatushka2-CAAX/+; sqh-mNeongreen-RhoGEF2 ΔPDZ, I1842E/+	sqh-mNeongreen-RhoGEF2 ΔPDZ, I1842E- **This study**
6G	nos-Lifeact-tdTomato-P2A-tdKatushka2-CAAX/+; sqh-mNeongreen-RhoGEF2 ΔPH, Anillin-RhoA binding domain (RBD)/+	sqh-mNeongreen-RhoGEF2 DPH, Anillin-RhoA binding domain (RBD)- **This study**
6M, Q, U	LexAop-degradFP-nosTCE-pgc 3'UTR/+; cn^{1} P{PZ}RhoGEF2^{04291}/Df(2R)BSC331;<br>MatTub-LexA-GAD, P{sqh-sfGFP-RhoGEF2}30/ nos-Lifeact-tdTomato-P2A-tdKatushka2-CAAX, sqh-mNeongreen-RhoGEF2 (female)<br>X<br>P{nullo-GAL4.G}5.20/+; P{w[+mC]=UASp-T7.RhoGEF2}5 (male)	
6N, S, W	LexAop-degradFP-nosTCE-pgc 3'UTR/+; cn^{1} P{PZ}RhoGEF2^{04291}/Df(2R)BSC331;<br>MatTub-LexA-GAD, P{sqh-sfGFP-RhoGEF2}30/ nos-Lifeact-tdTomato-P2A-tdKatushka2-CAAX, sqh-mNeongreen-RhoGEF2 I1842E (female)<br>X<br>P{nullo-GAL4.G}5.20/+; P{w[+mC]=UASp-T7.RhoGEF2}5 (male)	
6O, R, V	LexAop-degradFP-nosTCE-pgc 3'UTR/+; cn^{1} P{PZ}RhoGEF2^{04291}/Df(2R)BSC331;<br>MatTub-LexA-GAD, P{sqh-sfGFP-RhoGEF2}30/ nos-Lifeact-tdTomato-P2A-tdKatushka2-CAAX, sqh-mNeongreen-RhoGEF2 ΔPDZ (female)<br>X<br>P{nullo-GAL4.G}5.20/+; P{w[+mC]=UASp-T7.RhoGEF2}5 (male)	
7F (top), H	LexAop-degradFP-nosTCE-pgc 3'UTR/+; cn^{1} P{PZ}RhoGEF2^{04291}/Df(2R)BSC331;<br>MatTub-LexA-GAD, P{sqh-sfGFP-RhoGEF2}30/ nos-Lifeact-tdTomato-P2A-tdKatushka2-CAAX, sqh-mNeongreen-RhoGEF2 (female)<br>X<br>P{nullo-GAL4.G}5.20/+; P{w[+mC]=UASp-T7.RhoGEF2}5 (male)	
7F (middle), I	LexAop-degradFP-nosTCE-pgc 3'UTR/+; cn^{1} P{PZ}RhoGEF2^{04291}/Df(2R)BSC331;<br>MatTub-LexA-GAD, P{sqh-sfGFP-RhoGEF2}30/ nos-Lifeact-tdTomato-P2A-tdKatushka2-CAAX, sqh-mNeongreen-RhoGEF2 7E (female)<br>X<br>P{nullo-GAL4.G}5.20/+; P{w[+mC]=UASp-T7.RhoGEF2}5 (male)	sqh-mNeongreen-RhoGEF2-7E- **This study**
7F (bottom), J	LexAop-degradFP-nosTCE-pgc 3'UTR/+; cn^{1} P{PZ}RhoGEF2^{04291}/Df(2R)BSC331;<br>MatTub-LexA-GAD, P{sqh-sfGFP-RhoGEF2}30/ nos-Lifeact-tdTomato-P2A-tdKatushka2-CAAX, sqh-mNeongreen-RhoGEF2 7A (female)<br>X<br>P{nullo-GAL4.G}5.20/+; P{w[+mC]=UASp-T7.RhoGEF2}5 (male)	sqh-mNeongreen-RhoGEF2-7A- **This study**
8C (top)	Initial cross-<br>P{neoFRT}19A (female)<br>X<br>P{ovoD1-18}P4.1, P{hsFLP}12, y^{1} w^{1118} sn^{3} P{neoFRT}19A/C(1)DX, y^{1} f^{1} (male)<br><br>Progeny were heat shocked then the following virgin females were selected and mated as follows-<br>P{neoFRT}19A (female)<br>X<br>P{nullo-GAL4.G}5.20, P{w[+mC]=UAS-GFP-AMPKalpha}28E(male)	y1 w1118 P{neoFRT}19A- **BL1744**<br>P{ovoD1-18}P4.1, P{hsFLP}12, y^{1} w^{1118} sn^{3} P{neoFRT}19A/C(1)DX, y^{1} f^{1}- **BL23880**<br>P{w[+mC]=UAS-GFP-AMPKalpha}28E- **BL50745**
8C (bottom)	Initial cross-<br>P{neoFRT}19A, Ampkα^{D2}/Fm7c (female)<br>X<br>P{ovoD1-18}P4.1, P{hsFLP}12, y^{1} w^{1118} sn^{3} P{neoFRT}19A/C(1)DX, y^{1} f^{1} (male)<br><br>Progeny were heat shocked then the following virgin females were selected and mated as follows-<br>P{neoFRT}19A, Ampkα^{D2} (female)<br>X<br>P{nullo-GAL4.G}5.20, P{w[+mC]=UAS-GFP-AMPKalpha}28E(male)	Ampkα^{D2}- **Jongkyeong Chung** (*48*)**, provided by Daniela Drummond-Barbosa**
8E (top), G (top)	Initial cross-<br>P{neoFRT}19A; nos-Lifeact-tdTomato-P2A-tdKatushka2-CAAX (female)<br>X<br>P{ovoD1-18}P4.1, P{hsFLP}12, y^{1} w^{1118} sn^{3} P{neoFRT}19A/C(1)DX, y^{1} f^{1} (male)<br><br>Progeny were heat shocked then the following virgin females were selected and mated as follows-<br>P{neoFRT}19A; nos-Lifeact-tdTomato-P2A-tdKatushka2-CAAX/+ (female)<br>X<br>P{nullo-GAL4.G}5.20, P{w[+mC]=UAS-GFP-AMPKalpha}28E(male)	
8E (bottom), G (bottom)	Initial cross-<br>P{neoFRT}19A, Ampkα^{D2}/Fm7c; nos-Lifeact-tdTomato-P2A-tdKatushka2-CAAX (female)<br>X<br>P{ovoD1-18}P4.1, P{hsFLP}12, y^{1} w^{1118} sn^{3} P{neoFRT}19A/C(1)DX, y^{1} f^{1} (male)<br><br>Progeny were heat shocked then the following virgin females were selected and mated as follows-<br>P{neoFRT}19A, Ampkα^{D2}; nos-Lifeact-tdTomato-P2A-tdKatushka2-CAAX/+ (female)<br>X<br>P{nullo-GAL4.G}5.20, P{w[+mC]=UAS-GFP-AMPKalpha}28E(male)	

9A	Initial cross-<br>P{neoFRT}19A; nos-Lifeact-tdTomato-P2A-tdKatushka2-CAAX (female)<br>X<br>P{ovoD1-18}P4.1, P{hsFLP}12, y^{1} w^{1118} sn^{3} P{neoFRT}19A/C(1)DX, y^{1} f^{1} (male)<br><br>Progeny were heat shocked then the following virgin females were selected and mated as follows-<br>P{neoFRT}19A; nos-Lifeact-tdTomato-P2A-tdKatushka2-CAAX/+ (female)<br>X<br>P{nullo-GAL4.G}5.20, P{w[+mC]=UAS-GFP-AMPKalpha}28E(male)	
9B	Initial cross-<br>P{neoFRT}19A, Ampkα^{D2}/Fm7c; nos-Lifeact-tdTomato-P2A-tdKatushka2-CAAX (female)<br>X<br>P{ovoD1-18}P4.1, P{hsFLP}12, y^{1} w^{1118} sn^{3} P{neoFRT}19A/C(1)DX, y^{1} f^{1} (male)<br><br>Progeny were heat shocked then the following virgin females were selected and mated as follows-<br>P{neoFRT}19A, Ampkα^{D2}; nos-Lifeact-tdTomato-P2A-tdKatushka2-CAAX/+ (female)<br>X<br>P{nullo-GAL4.G}5.20, P{w[+mC]=UAS-GFP-AMPKalpha}28E(male)	
S1A	nos-Lifeact-tdTomato-P2A-tdKatushka2-CAAX; MyoII::3XGFP	
S1B, C	P{w[+mC]=GAL4::VP16-nos.UTR}CG6325[MVD1] (female)<br>X<br>P{UASp-hUTRN.ABD.EGFP} , P{w[+mC]=UAS-Rho1.V14}5.1 (male)	w[*]; P{w[+mC]=UAS-Rho1.V14}5.1- **BL7330**
S2	nos-Lifeact-tdTomato-P2A-tdKatushka2-CAAX; MyoII::3XGFP	
S3I, J	nos-Lifeact-tdTomato-P2A-tdKatushka2-CAAX; MyoII::3XGFP	
S4A	nos-Lifeact-tdTomato-P2A-tdKatushka2-CAAX/+; PBac{fTRG00591.sfGFP-TVPTBF}VK00033/+	PBac{fTRG00591.sfGFP-TVPTBF}VK00033- **(VDRC) v318165**
S4B	w^{1118}	
S5A (top)	tre1$^{ep\Delta5}$/+; cn^{1} P{PZ}RhoGEF2^{04291}/+ (female)<br>X<br>tre1$^{ep\Delta5}$ (male)	
S5B (bottom)	tre1$^{ep\Delta5}$/+ (female)<br>X<br>tre1$^{ep\Delta5}$ (male)	
S5C (top)	**RG2 control-** cn^{1} P{PZ}RhoGEF2^{04291}/Df(2R)BSC331;<br>LexAop-degradFP-nosTCE-pgc 3'UTR, P{sqh-sfGFP-RhoGEF2}30/ nos-Lifeact-tdTomato-P2A-tdKatushka2-CAAX (female)<br>X<br>P{nullo-GAL4.G}5.20/+; P{w[+mC]=UASp-T7.RhoGEF2}5 (male)	
S5C (bottom)	**RG2 degradation-** cn^{1} P{PZ}RhoGEF2^{04291}/Df(2R)BSC331;<br>LexAop-degradFP-nosTCE-pgc 3'UTR, P{sqh-sfGFP-RhoGEF2}30/ nos-Lifeact-tdTomato-P2A-tdKatushka2-CAAX, MatTub-LexA-GAD (female)<br>X<br>P{nullo-GAL4.G}5.20/+; P{w[+mC]=UASp-T7.RhoGEF2}5 (male)	
S5E (top)	**RG2 control-** cn^{1} P{PZ}RhoGEF2^{04291}/Df(2R)BSC331;<br>LexAop-degradFP-nosTCE-pgc 3'UTR, P{sqh-sfGFP-RhoGEF2}30/ nos-tdTomato-Anillin-RBD-P2A-tdKatushka2-CAAX (female)<br>X<br>P{nullo-GAL4.G}5.20/+; P{w[+mC]=UASp-T7.RhoGEF2}5 (male)	nos-tdTomato-Anillin-RBD-P2A-tdKatushka2-CAAX-**Ruth Lehmann** (*61*)
S5E (bottom)	**RG2 degradation-** cn^{1} P{PZ}RhoGEF2^{04291}/Df(2R)BSC331;<br>LexAop-degradFP-nosTCE-pgc 3'UTR, P{sqh-sfGFP-RhoGEF2}30/ nos-tdTomato-Anillin-RBD-P2A-tdKatushka2-CAAXX, MatTub-LexA-GAD (female)<br>X<br>P{nullo-GAL4.G}5.20/+; P{w[+mC]=UASp-T7.RhoGEF2}5 (male)	
S6A	P{matα4-GAL-VP16}67, nos-tdTomato-Anillin-RBD-P2A-tdKatushka2-CAAX/+ ;<br>P{matα4-GAL-VP16}15/+	
S6B	P{matα4-GAL-VP16}67, nos-tdTomato-Anillin-RBD-P2A-tdKatushka2-CAAX/ UASp-G$\alpha_{12/13}$-Q303L-*nos* TCE-*pgc* 3'UTR;<br>P{matα4-GAL-VP16}15/+	
S7D	negative control- w^{1118}<br>All transgenes are heterozygous- squ-mNeongreen-RhoGEF2 transgene/+	squ-mNeongreen-RhoGEF2-ΔC1- **This study**<br>squ-mNeongreen-RhoGEF2-ΔRGS- **This study**<br>squ-mNeongreen-RhoGEF2-F1840A- **This study**
S7F (top left)	nos-Lifeact-tdTomato-P2A-tdKatushka2-CAAX/+; sqh-mNeongreen-RhoGEF2/+	
S7F (top right), H	nos-Lifeact-tdTomato-P2A-tdKatushka2-CAAX/+; sqh-mNeongreen-RhoGEF2 ΔC1/+	
S7F (bottom left)	nos-Lifeact-tdTomato-P2A-tdKatushka2-CAAX/+; sqh-mNeongreen-RhoGEF2 ΔPDZ/+	
S7F (bottom right), J	nos-Lifeact-tdTomato-P2A-tdKatushka2-CAAX/+; sqh-mNeongreen-RhoGEF2 ΔRGS/+	
S8G (left)	nos-Lifeact-tdTomato-P2A-tdKatushka2-CAAX/+; sqh-mNeongreen-RhoGEF2 I1842E/+	
S8G (right), I	nos-Lifeact-tdTomato-P2A-tdKatushka2-CAAX/+; sqh-mNeongreen-RhoGEF2 F1840A/+	
S9A	LexAop-degradFP-nosTCE-pgc 3'UTR; cn^{1} P{PZ}RhoGEF2^{04291}/Df(2R)BSC331;<br>MatTub-LexA-GAD, P{sqh-sfGFP-RhoGEF2}30/ nos-Lifeact-tdTomato-P2A-tdKatushka2-CAAX, sqh-mNeongreen-RhoGEF2 (female)<br>X<br>P{nullo-GAL4.G}5.20/+; P{w[+mC]=UASp-T7.RhoGEF2}5 (male)	
S9B	LexAop-degradFP-nosTCE-pgc 3'UTR; cn^{1} P{PZ}RhoGEF2^{04291}/Df(2R)BSC331;<br>MatTub-LexA-GAD, P{sqh-sfGFP-RhoGEF2}30/ nos-Lifeact-tdTomato-P2A-tdKatushka2-CAAX, sqh-mNeongreen-RhoGEF2 ΔPDZ (female)<br>X<br>P{nullo-GAL4.G}5.20/+; P{w[+mC]=UASp-T7.RhoGEF2}5 (male)	
S9C	LexAop-degradFP-nosTCE-pgc 3'UTR; cn^{1} P{PZ}RhoGEF2^{04291}/Df(2R)BSC331;<br>MatTub-LexA-GAD, P{sqh-sfGFP-RhoGEF2}30/ nos-Lifeact-tdTomato-P2A-tdKatushka2-CAAX, sqh-mNeongreen-RhoGEF2 I1842E (female)<br>X<br>P{nullo-GAL4.G}5.20/+; P{w[+mC]=UASp-T7.RhoGEF2}5 (male)	
S10A-B	P{w[+mC]=UAS-Eb1.EGFP.H}G/ MatTub-gal4; P{UASp-RhoGEF2.RFP}/+	P{w[+mC]=UAS-Eb1.EGFP.H}G- **BL36861**<br>MatTub-gal4- **Ruth Lehmann** (*24*)<br>P{UASp-RhoGEF2.RFP}- **Jörg Großhans** (*40*)
S10D	P{w[+mC]=GAL4::VP16-nos.UTR}CG6325[MVD1] (female)<br>X<br>P{w[+mC]=UAS-nod.GFP}2 (male)	P{w[+mC]=UAS-nod.GFP}2- **BL9282**
S10E	P{w[+mC]=GAL4::VP16-nos.UTR}CG6325[MVD1] (female)<br>X<br>P{w[+mC]=UAS-Eb1.EGFP.H}G (male)	
S11G	negative control- w1118<br>All transgenes are heterozygous- squ-mNeongreen-RhoGEF2 transgene/+	squ-mNeongreen-RhoGEF2-SKNN- **This study**
S11I	nos-Lifeact-tdTomato-P2A-tdKatushka2-CAAX/+; sqh-mNeongreen-RhoGEF2 SKNN/+	
S11J	nos-Lifeact-tdTomato-P2A-tdKatushka2-CAAX/+; sqh-mNeongreen-RhoGEF2 7E/+	
S11K	nos-Lifeact-tdTomato-P2A-tdKatushka2-CAAX/+; sqh-mNeongreen-RhoGEF2 7A/+	
S11O	LexAop-degradFP-nosTCE-pgc 3'UTR; cn1 P{PZ}RhoGEF204291/Df(2R)BSC331;<br>MatTub-LexA-GAD, P{sqh-sfGFP-RhoGEF2}30/ nos-Lifeact-tdTomato-P2A-tdKatushka2-CAAX, sqh-mNeongreen-RhoGEF2 (female)<br>X<br>P{nullo-GAL4.G}5.20/+; P{w[+mC]=UASp-T7.RhoGEF2}5 (male)	
S11P	LexAop-degradFP-nosTCE-pgc 3'UTR; cn^{1} P{PZ}RhoGEF204291/Df(2R)BSC331;<br>MatTub-LexA-GAD, P{sqh-sfGFP-RhoGEF2}30/ nos-Lifeact-tdTomato-P2A-tdKatushka2-CAAX, sqh-mNeongreen-RhoGEF2 7E (female)<br>X<br>P{nullo-GAL4.G}5.20/+; P{w[+mC]=UASp-T7.RhoGEF2}5 (male)	
S11Q	LexAop-degradFP-nosTCE-pgc 3'UTR; cn^{1} P{PZ}RhoGEF204291/Df(2R)BSC331;<br>MatTub-LexA-GAD, P{sqh-sfGFP-RhoGEF2}30/ nos-Lifeact-tdTomato-P2A-tdKatushka2-CAAX, sqh-mNeongreen-RhoGEF2 7A (female)<br>X<br>P{nullo-GAL4.G}5.20/+; P{w[+mC]=UASp-T7.RhoGEF2}5 (male)	

S12A	LexAop-degradFP-nosTCE-pgc 3'UTR; cn^{1} P{PZ}RhoGEF204291/Df(2R)BSC331;<br>MatTub-LexA-GAD, P{sqh-sfGFP-RhoGEF2}30/ nos-Lifeact-tdTomato-P2A-tdKatushka2-CAAX, sqh-mNeongreen-RhoGEF2 (female)<br>X<br>P{nullo-GAL4.G}5.20/+; P{w[+mC]=UASp-T7.RhoGEF2}5 (male)	
S12B	LexAop-degradFP-nosTCE-pgc 3'UTR; cn^{1} P{PZ}RhoGEF204291/Df(2R)BSC331;<br>MatTub-LexA-GAD, P{sqh-sfGFP-RhoGEF2}30/ nos-Lifeact-tdTomato-P2A-tdKatushka2-CAAX, sqh-mNeongreen-RhoGEF2 7E (female)<br>X<br>P{nullo-GAL4.G}5.20/+; P{w[+mC]=UASp-T7.RhoGEF2}5 (male)	
S13A-B	**RG2 control**- cn^{1} P{PZ}RhoGEF2^{04291}/Df(2R)BSC331;<br>LexAop-degradFP-*nos* TCE-*pgc* 3'UTR, P{sqh-sfGFP-RhoGEF2}30/ nos-Lifeact-tdTomato-P2A-tdKatushka2-CAAX (female)<br>X<br>P{nullo-GAL4.G}5.20/+; P{w[+mC]=UASp-T7.RhoGEF2}5 (male)	
S13C-D	**RG2 degradation**- cn^{1} P{PZ}RhoGEF2^{04291}/Df(2R)BSC331;<br>LexAop-degradFP-*nos* TCE-*pgc* 3'UTR, P{sqh-sfGFP-RhoGEF2}30/ nos-Lifeact-tdTomato-P2A-tdKatushka2-CAAX, MatTub-LexA-GAD (female)<br>X<br>P{nullo-GAL4.G}5.20/+; P{w[+mC]=UASp-T7.RhoGEF2}5 (male)	

Genotypes for all experiments and their origin.

Supplementary Movie 1- Retrograde cortical actin flow in a migrating PGC
Representative two-photon time-lapse imaging of the dorsal plane of a PGC expressing utrophin-GFP migrating toward the mesoderm. Times are in seconds.

Supplementary Movie 2- Retrograde cortical myosin II flow in a migrating PGC
Representative two-photon time-lapse imaging of the medial plane of a PGC expressing myosin II-3xGFP migrating toward the mesoderm. Times are in seconds.

Supplementary Movie 3- Cortical actin flow in a PGC expressing constitutively active Rac1
Representative two-photon time-lapse imaging of cortical flow along the dorsal plane of a PGC expressing utrophin-GFP along with Rac1 WT (left) or constitutively active Rac1 (Rac1-CA) (right). Note the appearance of filamentous like structures when overexpressing Rac1-CA. Times are in seconds.

Supplementary Movie 4- Cortical actin flow in a PGC expressing constitutively active or dominant negative RhoA
Representative two-photon time-lapse imaging of cortical flow along the dorsal plane of a PGC expressing utrophin-GFP alone (left) or along with constitutively active RhoA (RhoA-CA) (middle) or dominant negative RhoA (RhoA-DN) (right). Note that the PGC cortex becomes homogeneous when overexpressing Rho1-CA. Cortical filaments can be observed when overexpressing RhoA-DN. Times are in seconds.

Supplementary Movie 5- Circular cortical flows in an extracted PGC on a coverslip without serum stimulation
Representative time-lapse imaging of an extracted PGC expressing lifeact-tdTomato and myosin II-3xGFP seeded onto a coverslip displaying circular cortical flow. Times are in seconds.

Supplementary Movie 6- Cortical flows in an extracted PGC under agarose with Y-27632 treatment.
Representative time-lapse imaging of an extracted PGC expressing lifeact-tdTomato (top left in a given set of 4 images) and myosin II-3xGFP (bottom left in a given set of 4 images) under an agarose gel displaying cortical flow under control (left set of 4 images) or Y-27632 treatment (right set of 4 images). Particle image velicometry (PIV) analysis of the actin (top right in a given set of 4 images) and myosin II (bottom right in a given set of 4 images) networks are shown on the right. Red colors indicate fast flow magnitudes, while blue colors indicate slow flow magnitudes. White arrows are vector magnitudes which scale with flow magnitude and show flow orientation. Times are in seconds.

Supplementary Movie 7- Cortical flows in an extracted PGC under agarose with Cytochalasin D treatment.
Representative time-lapse imaging of an extracted PGC expressing lifeact-tdTomato (top left in a given set of 4 images) and myosin II-3xGFP (bottom left in a given set of 4 images) under an agarose gel displaying cortical flow under DMSO (left set of 4 images) or Cytochalasin-D treatment (right set of 4 images). Particle image velicometry (PIV) analysis of the actin (top right in a given set of 4 images) and myosin II (bottom right in a given set of 4 images) networks are

shown on the right. Red colors indicate fast flow magnitudes, while blue colors indicate slow flow magnitudes. White arrows are vector magnitudes which scale with flow magnitude and show flow orientation. Times are in seconds.

Supplementary Movie 8- Cortical flows in an extracted PGC under agarose with CK-666 and SMIFH2 treatment.
Representative time-lapse imaging of an extracted PGC expressing lifeact-tdTomato (top left in a given set of 4 images) and myosin II-3xGFP (bottom left in a given set of 4 images) under an agarose gel displaying cortical flow under DMSO (left set of 4 images), CK-666 (middle set of 4 images), or SMIFH2 treatment (right set of 4 images). Particle image velicometry (PIV) analysis of the actin (top right in a given set of 4 images) and myosin II (bottom right in a given set of 4 images) networks are shown on the right. Red colors indicate fast flow magnitudes, while blue colors indicate slow flow magnitudes. White arrows are vector magnitudes which scale with flow magnitude and show flow orientation. Times are in seconds.

Supplementary Movie 9- Localization of RhoGEF2 during PGC cluster dispersal
Representative two-photon time-lapse imaging of a dispersing PGC cluster expressing tdKatushka2-CAAX (PGC membrane marker) and sfGFP-RhoGEF2. sfGFP-RhoGEF2 is enriched at the rear of PGCs. Times are in minutes.

Supplementary Movie 10- Localization of RhoGEF2 during directed PGC migration toward the mesoderm
Representative two-photon time-lapse imaging of PGCs expressing tdKatushka2-CAAX (PGC membrane marker) and sfGFP-RhoGEF2 migrating toward the mesoderm. sfGFP-RhoGEF2 is enriched at the rear of PGCs. Times are in minutes.

Supplementary Movie 11- PGC cluster dispersal with and without RhoGEF2
Representative two-photon time-lapse imaging of dispersing PGC clusters expressing tdKatushka2-CAAX (PGC membrane marker) under control (left) and RhoGEF2 degradation (right) conditions. Times are in minutes.

Supplementary Movie 12- Directed PGC migration toward the mesoderm with and without RhoGEF2
Representative two-photon time-lapse imaging of PGCs expressing tdKatushka2-CAAX (PGC membrane marker) under control (left) and RhoGEF2 degradation (right) conditions migrating toward the mesoderm. Times are in minutes.

Supplementary Movie 13- Cortical flows in an extracted PGC under agarose with and without RhoGEF2
Representative time-lapse imaging of a control (top left) or RhoGEF2 degraded (bottom left) extracted PGC expressing lifeact-tdTomato under an agarose gel displaying cortical flow. Particle image velicometry (PIV) analysis of the actin networks are shown on the right. Red colors indicate fast flow magnitudes, while blue colors indicate slow flow magnitudes. White arrows are vector magnitudes which scale with flow magnitude and show flow orientation. Times are in seconds.

Supplementary Movie 14- Directed PGC migration toward the mesoderm with RhoGEF2 activation
Representative two-photon time-lapse imaging of PGCs expressing myosin-II tdTomato under control (left) and RhoGEF2 activation (constitutively active $G\alpha_{12/13}$, $G\alpha_{12/13}$-Q303L, right) conditions migrating toward the mesoderm. Times are in minutes.

Supplementary Movie 15- Cortical flows in an extracted PGC under agarose with RhoGEF2 activation
Representative time-lapse imaging of a control (top left) or RhoGEF2 activated (constitutively active $G\alpha_{12/13}$, $G\alpha_{12/13}$-Q303L, bottom left) extracted PGC expressing myosin-II tdTomato under an agarose gel displaying cortical flow. Particle image velicometry (PIV) analysis of the myosin II networks are shown on the right. Red colors indicate fast flow magnitudes, while blue colors indicate slow flow magnitudes. White arrows are vector magnitudes which scale with flow magnitude and show flow orientation. Times are in seconds.

Supplementary Movie 16- PGC cluster dispersal with different RhoGEF2 domain perturbations
Representative two-photon time-lapse imaging of dispersing PGC clusters expressing tdKatushka2-CAAX (PGC membrane marker) rescued with RhoGEF2 WT (left), RhoGEF2 ΔPDZ (middle), or RhoGEF2 I1842E (right) transgenes. Times are in minutes.

Supplementary Movie 17- Directed PGC migration toward the mesoderm with different RhoGEF2 domain perturbations
Representative two-photon time-lapse imaging of PGCs expressing tdKatushka2-CAAX (PGC membrane marker) rescued with RhoGEF2 WT (left), RhoGEF2 ΔPDZ (middle), or RhoGEF2 I1842E (right) transgenes migrating towards the mesoderm. Times are in minutes.

Supplementary Movie 18- RhoGEF2 microtubule plus-end tracking in PGCs with and without colchicine
Representative extracted PGCs expressing EB1-GFP and RFP-RhoGEF2 under control (ethanol, top) or colchicine (bottom) treatment. Times are in seconds.

Supplementary Movie 19- PGCs expressing NOD-GFP migrating toward the mesoderm
Representative two photon time-lapse imaging of PGCs expressing NOD-GFP (microtubule minus-end marker) migrating towards the mesoderm. A maximum intensity Z-projection is shown. Times are in minutes.

Supplementary Movie 20- PGCs expressing EB1-GFP migrating toward the mesoderm
Representative two photon time-lapse imaging of the dorsal plane of a PGC expressing EB1-GFP (microtubule plus-end marker) migrating towards the mesoderm. Times are in seconds.

Supplementary Movie 21- Directed PGC migration toward the mesoderm with different RhoGEF2 microtubule plus-end tracking mutants
Representative two photon time-lapse imaging of PGCs expressing tdKatushka2-CAAX (PGC membrane marker) rescued with RhoGEF2 WT (left), RhoGEF2 7E (middle), or RhoGEF2 7A (right) transgenes migrating towards the mesoderm. Times are in minutes.

Supplementary Movie 22- PGC cluster dispersal with and without AMPK
Representative two photon time-lapse imaging of dispersing PGC clusters expressing tdKatushka2-CAAX (PGC membrane marker) with (left) and without (right) AMPK. Times are in minutes.

Supplementary Movie 23- Directed PGC migration toward the mesoderm with and without AMPK
Representative two photon time-lapse imaging of PGCs expressing tdKatushka2-CAAX (PGC membrane marker) with (left) and without (right) AMPK migrating towards the mesoderm. Times are in minutes.

Supplementary Movie 24- Cortical flows in an extracted PGC under agarose with and without AMPK
Representative time-lapse imaging of an extracted PGC with (top left) or without (bottom left) AMPK expressing lifeact-tdTomato under an agarose gel displaying cortical flow. Particle image velicometry (PIV) analysis of the actin networks are shown on the right. Red colors indicate fast flow magnitudes, while blue colors indicate slow flow magnitudes. White arrows are vector magnitudes which scale with flow magnitude and show flow orientation. Times are in seconds.

Supplementary Movie 25- Extracted control PGCs with and without AMPK activation
Representative time-lapse imaging of extracted control PGCs expressing lifeact-tdTomato under control (DMSO, left) or AMPK activation (A-769662, right) conditions. Times are in seconds.

Supplementary Movie 26- Extracted RhoGEF2 degraded PGCs with and without AMPK activation
Representative time-lapse imaging of extracted RhoGEF2 degraded PGCs expressing lifeact-tdTomato under control (DMSO, left) or AMPK activation (A-769662, right) conditions. Times are in seconds.

REFERENCES AND NOTES

1. L. B. Case, C. M. Waterman, Integration of actin dynamics and cell adhesion by a three-dimensional, mechanosensitive molecular clutch. *Nat. Cell Biol.* **17**, 955–963 (2015).

2. R. Poincloux, O. Collin, F. Lizárraga, M. Romao, M. Debray, M. Piel, P. Chavrier, Contractility of the cell rear drives invasion of breast tumor cells in 3D Matrigel. *Proc. Natl. Acad. Sci. U.S.A.* **108**, 1943–1948 (2011).

3. P. R. O'Neill, J. A. Castillo-Badillo, X. Meshik, V. Kalyanaraman, K. Melgarejo, N. Gautam, Membrane flow drives an adhesion-independent amoeboid cell migration mode. *Dev. Cell* **46**, 9–22.e24 (2018).

4. W. Shih, S. Yamada, Myosin IIA dependent retrograde flow drives 3D cell migration. *Biophys. J.* **98**, L29–L31 (2010).

5. Y.-J. Liu, M. Le Berre, F. Lautenschlaeger, P. Maiuri, A. Callan-Jones, M. Heuzé, T. Takaki, R. Voituriez, M. Piel, Confinement and low adhesion induce fast amoeboid migration of slow mesenchymal cells. *Cell* **160**, 659–672 (2015).

6. V. Ruprecht, S. Wieser, A. Callan-Jones, M. Smutny, H. Morita, K. Sako, V. Barone, M. Ritsch-Marte, M. Sixt, R. Voituriez, C.-P. Heisenberg, Cortical contractility triggers a stochastic switch to fast amoeboid cell motility. *Cell* **160**, 673–685 (2015).

7. J. S. Logue, A. X. Cartagena-Rivera, M. A. Baird, M. W. Davidson, R. S. Chadwick, C. M. Waterman, Erk regulation of actin capping and bundling by Eps8 promotes cortex tension and leader bleb-based migration. *eLife* **4**, e08314 (2015).

8. M. Tozluoğlu, A. L. Tournier, R. P. Jenkins, S. Hooper, P. A. Bates, E. Sahai, Matrix geometry determines optimal cancer cell migration strategy and modulates response to interventions. *Nat. Cell Biol.* **15**, 751–762 (2013).

9. M. Bergert, A. Erzberger, R. A. Desai, I. M. Aspalter, A. C. Oates, G. Charras, G. Salbreux, E. K. Paluch, Force transmission during adhesion-independent migration. *Nat. Cell Biol.* **17**, 524–529 (2015).

10. V. Venturini, F. Pezzano, F. Català Castro, H.-M. Häkkinen, S. Jiménez-Delgado, M. Colomer-Rosell, M. Marro, Q. Tolosa-Ramon, S. Paz-López, A. Valverde Miguel, J. Weghuber, P. Loza-Alvarez, M. Krieg, S. Wieser, V. Ruprecht, The nucleus measures shape changes for cellular proprioception to control dynamic cell behavior. *Science* **370**, eaba2644 (2020).

11. A. J. Lomakin, C. J. Cattin, D. Cuvelier, Z. Alraies, M. Molina, G. P. F. Nader, N. Srivastava, P. J. Sáez, J. M. Garcia-Arcos, I. Y. Zhitnyak, A. Bhargava, M. K. Driscoll, E. S. Welf, R. Fiolka, R. J. Petrie, N. S. De Silva, J. M. González-Granado, N. Manel, A. M. Lennon-Duménil, D. J. Müller, M. Piel, The nucleus acts as a ruler tailoring cell responses to spatial constraints. *Science* **370**, eaba2894 (2020).

12. E. Kardash, M. Reichman-Fried, J.-L. Maître, B. Boldajipour, E. Papusheva, E.-M. Messerschmidt, C.-P. Heisenberg, E. Raz, A role for Rho GTPases and cell–cell adhesion in single-cell motility *in vivo*. *Nat. Cell Biol.* **12**, 47–53 (2010).

13. M. Mayer, M. Depken, J. S. Bois, F. Jülicher, S. W. Grill, Anisotropies in cortical tension reveal the physical basis of polarizing cortical flows. *Nature* **467**, 617–621 (2010).

14. P. Maiuri, J.-F. Rupprecht, S. Wieser, V. Ruprecht, O. Bénichou, N. Carpi, M. Coppey, S. De Beco, N. Gov, C.-P. Heisenberg, C. Lage Crespo, F. Lautenschlaeger, M. Le Berre, A.-M. Lennon-Dumenil, M. Raab, H.-R. Thiam, M. Piel, M. Sixt, R. Voituriez, Actin flows mediate a universal coupling between cell speed and cell persistence. *Cell* **161**, 374–386 (2015).

15. A. C. Callan-Jones, R. Voituriez, Actin flows in cell migration: From locomotion and polarity to trajectories. *Curr. Opin. Cell Biol.* **38**, 12–17 (2016).

16. M. F. Ullo, J. S. Logue, ADF and cofilin-1 collaborate to promote cortical actin flow and the leader bleb-based migration of confined cells. *eLife* **10**, e67856 (2021).

17. F. Medina, A. M. Carter, O. Dada, S. Gutowski, J. Hadas, Z. Chen, P. C. Sternweis, Activated RhoA is a positive feedback regulator of the Lbc family of Rho guanine nucleotide exchange factor proteins. *J. Biol. Chem.* **288**, 11325–11333 (2013).

18. K. Wong, A. Van Keymeulen, H. R. Bourne, PDZRhoGEF and myosin II localize RhoA activity to the back of polarizing neutrophil-like cells. *J. Cell Biol.* **179**, 1141–1148 (2007).

19. C. D. Wells, M.-Y. Liu, M. Jackson, S. Gutowski, P. M. Sternweis, J. D. Rothstein, T. Kozasa, P. C. Sternweis, Mechanisms for reversible regulation between G13 and Rho exchange factors. *J. Biol. Chem.* **277**, 1174–1181 (2002).

20. L. J. Barton, M. G. LeBlanc, R. Lehmann, Finding their way: Themes in germ cell migration. *Curr. Opin. Cell Biol.* **42**, 128–137 (2016).

21. B. E. Richardson, R. Lehmann, Mechanisms guiding primordial germ cell migration: Strategies from different organisms. *Nat. Rev. Mol. Cell Biol.* **11**, 37–49 (2010).

22. H. Blaser, M. Reichman-Fried, I. Castanon, K. Dumstrei, F. L. Marlow, K. Kawakami, L. Solnica-Krezel, C.-P. Heisenberg, E. Raz, Migration of Zebrafish primordial germ cells: A role for myosin contraction and cytoplasmic flow. *Dev. Cell* **11**, 613–627 (2006).

23. A. Olguin-Olguin, A. Aalto, B. Maugis, A. Boquet-Pujadas, D. Hoffmann, L. Ermlich, T. Betz, N. S. Gov, M. Reichman-Fried, E. Raz, Chemokine-biased robust self-organizing polarization of migrating cells in vivo. *Proc. Natl. Acad. Sci. U.S.A.* **118**, e2018480118 (2021).

24. B. Lin, J. Luo, R. Lehmann, Collectively stabilizing and orienting posterior migratory forces disperses cell clusters in vivo. *Nat. Commun.* **11**, 4477 (2020).

25. P. S. Kunwar, M. Starz-Gaiano, R. J. Bainton, U. Heberlein, R. Lehmann, Tre1, a G protein-coupled receptor, directs transepithelial migration of Drosophila germ cells. *PLOS Biol.* **1**, E80 (2003).

26. J. H. Henson, M. Yeterian, R. M. Weeks, A. E. Medrano, B. L. Brown, H. L. Geist, M. D. Pais, R. Oldenbourg, C. B. Shuster, Arp2/3 complex inhibition radically alters lamellipodial actin

architecture, suspended cell shape, and the cell spreading process. *Mol. Biol. Cell* **26**, 887–900 (2015).

27. M. Hons, A. Kopf, R. Hauschild, A. Leithner, F. Gaertner, J. Abe, J. Renkawitz, J. V. Stein, M. Sixt, Chemokines and integrins independently tune actin flow and substrate friction during intranodal migration of T cells. *Nat. Immunol.* **19**, 606–616 (2018).

28. P. S. Kunwar, H. Sano, A. D. Renault, V. Barbosa, N. Fuse, R. Lehmann, Tre1 GPCR initiates germ cell transepithelial migration by regulating Drosophila melanogaster E-cadherin. *J. Cell Biol.* **183**, 157–168 (2008).

29. M. G. LeBlanc, R. Lehmann, Domain-specific control of germ cell polarity and migration by multifunction Tre1 GPCR. *J. Cell Biol.* **216**, 2945–2958 (2017).

30. J. H. Kim, C. D. Hanlon, S. Vohra, P. N. Devreotes, D. J. Andrew, Hedgehog signaling and Tre1 regulate actin dynamics through PI(4,5)P_2 to direct migration of *Drosophila* embryonic germ cells. *Cell Rep.* **34**, 108799 (2021).

31. E. Caussinus, O. Kanca, M. Affolter, Fluorescent fusion protein knockout mediated by anti-GFP nanobody. *Nat. Struct. Mol. Biol.* **19**, 117–121 (2012).

32. J. Großhans, C. Wenzl, H.-M. Herz, S. Bartoszewski, F. Schnorrer, N. Vogt, H. Schwarz, H.-A. Müller, RhoGEF2 and the formin Dia control the formation of the furrow canal by directed actin assembly during Drosophila cellularisation. *Development* **132**, 1009–1020 (2005).

33. K. Barrett, M. Leptin, J. Settleman, The Rho GTPase and a Putative RhoGEF mediate a signaling pathway for the cell shape changes in Drosophila Gastrulation. *Cell* **91**, 905–915 (1997).

34. U. Häcker, N. Perrimon, DRhoGEF2 encodes a member of the Dbl family of oncogenes and controls cell shape changes during gastrulation in Drosophila. *Genes Dev.* **12**, 274–284 (1998).

35. R. E. Dawes-Hoang, K. M. Parmar, A. E. Christiansen, C. B. Phelps, A. H. Brand, E. F. Wieschaus, *Folded gastrulation*, cell shape change and the control of myosin localization. *Development* **132**, 4165–4178 (2005).

36. S. Parks, E. Wieschaus, The drosophila gastrulation gene concertina encodes a Gα-like protein. *Cell* **64**, 447–458 (1991).

37. A. J. Manning, K. A. Peters, M. Peifer, S. L. Rogers, Regulation of epithelial morphogenesis by the G protein-coupled receptor mist and its ligand fog. *Sci. Signal.* **6**, ra98 (2013).

38. N. Fuse, F. Yu, S. Hirose, Gprk2 adjusts Fog signaling to organize cell movements in *Drosophila* gastrulation. *Development* **140**, 4246–4255 (2013).

39. V. Kölsch, T. Seher, G. J. Fernandez-Ballester, L. Serrano, M. Leptin, Control of *Drosophila* Gastrulation by Apical localization of adherens junctions and RhoGEF2. *Science* **315**, 384–386 (2007).

40. C. Wenzl, S. Yan, P. Laupsien, J. Großhans, Localization of RhoGEF2 during *Drosophila* cellularization is developmentally controlled by *slam*. *Mech. Dev.* **127**, 371–384 (2010).

41. J. A. Stein, H. T. Broihier, L. A. Moore, R. Lehmann, Slow as Molasses is required for polarized membrane growth and germ cell migration in *Drosophila*. *Development* **129**, 3925–3934 (2002).

42. A. Rich, R. G. Fehon, M. Glotzer, Rho1 activation recapitulates early gastrulation events in the ventral, but not dorsal, epithelium of *Drosophila* embryos. *eLife* **9**, e56893 (2020).

43. S. L. Rogers, U. Wiedemann, U. Häcker, C. Turck, R. D. Vale, *Drosophila* RhoGEF2 associates with microtubule plus ends in an EB1-dependent manner. *Curr. Biol.* **14**, 1827–1833 (2004).

44. I. E. Clark, L. Y. Jan, Y. N. Jan, Reciprocal localization of Nod and kinesin fusion proteins indicates microtubule polarity in the Drosophila oocyte, epithelium, neuron and muscle. *Development* **124**, 461–470 (1997).

45. S. Honnappa, S. M. Gouveia, A. Weisbrich, F. F. Damberger, N. S. Bhavesh, H. Jawhari, I. Grigoriev, F. J. A. van Rijssel, R. M. Buey, A. Lawera, I. Jelesarov, F. K. Winkler, K. Wüthrich, A. Akhmanova, M. O. Steinmetz, An EB1-binding motif acts as a microtubule tip localization signal. *Cell* **138**, 366–376 (2009).

46. Y. Hu, R. Sopko, V. Chung, M. Foos, R. A. Studer, S. D. Landry, D. Liu, L. Rabinow, F. Gnad, P. Beltrao, N. Perrimon, iProteinDB: An integrative database of *Drosophila* post-translational modifications. *G3 (Bethesda.)* **9**, 1–11 (2019).

47. A. Nakano, H. Kato, T. Watanabe, K.-D. Min, S. Yamazaki, Y. Asano, O. Seguchi, S. Higo, Y. Shintani, H. Asanuma, M. Asakura, T. Minamino, K. Kaibuchi, N. Mochizuki, M. Kitakaze, S. Takashima, AMPK controls the speed of microtubule polymerization and directional cell migration through CLIP-170 phosphorylation. *Nat. Cell Biol.* **12**, 583–590 (2010).

48. J. H. Lee, H. Koh, M. Kim, Y. Kim, S. Y. Lee, R. E. Karess, S.-H. Lee, M. Shong, J.-M. Kim, J. Kim, J. Chung, Energy-dependent regulation of cell structure by AMP-activated protein kinase. *Nature* **447**, 1017–1020 (2007).

49. S. A. Koestler, A. Steffen, M. Nemethova, M. Winterhoff, N. Luo, J. M. Holleboom, J. Krupp, S. Jacob, M. Vinzenz, F. Schur, K. Schlüter, P. W. Gunning, C. Winkler, C. Schmeiser, J. Faix, T. E. B. Stradal, J. V. Small, K. Rottner, Arp2/3 complex is essential for actin network treadmilling as well as for targeting of capping protein and cofilin. *Mol. Biol. Cell* **24**, 2861–2875 (2013).

50. G. H. Koenderink, E. K. Paluch, Architecture shapes contractility in actomyosin networks. *Curr. Opin. Cell Biol.* **50**, 79–85 (2018).

51. W. Y. Ding, H. T. Ong, Y. Hara, J. Wongsantichon, Y. Toyama, R. C. Robinson, F. Nédélec, R. Zaidel-Bar, Plastin increases cortical connectivity to facilitate robust polarization and timely cytokinesis. *J. Cell Biol.* **216**, 1371–1386 (2017).

52. H. Ennomani, G. Letort, C. Guérin, J.-L. Martiel, W. Cao, F. Nédélec, E. M. De La Cruz, M. Théry, L. Blanchoin, Architecture and connectivity govern actin network contractility. *Curr. Biol.* **26**, 616–626 (2016).

53. H. A. Benink, C. A. Mandato, W. M. Bement, Analysis of cortical flow models in vivo. *Mol. Biol. Cell* **11**, 2553–2563 (2000).

54. W. J. Gan, F. Motegi, Mechanochemical control of symmetry breaking in the *Caenorhabditis elegans* zygote. *Front. Cell Dev. Biol.* **8**, 619869 (2021).

55. J. Renkawitz, K. Schumann, M. Weber, T. Lämmermann, H. Pflicke, M. Piel, J. Polleux, J. P. Spatz, M. Sixt, Adaptive force transmission in amoeboid cell migration. *Nat. Cell Biol.* **11**, 1438–1443 (2009).

56. A. Franz, W. Wood, P. Martin, Fat body cells are motile and actively migrate to wounds to drive repair and prevent infection. *Dev. Cell* **44**, 460–470.e3 (2018).

57. B. D. Pfeiffer, T.-T. B. Ngo, K. L. Hibbard, C. Murphy, A. Jenett, J. W. Truman, G. M. Rubin, Refinement of tools for targeted gene expression in Drosophila. *Genetics* **186**, 735–755 (2010).

58. J.-Q. Ni, M. Markstein, R. Binari, B. Pfeiffer, L.-P. Liu, C. Villalta, M. Booker, L. Perkins, N. Perrimon, Vector and parameters for targeted transgenic RNA interference in *Drosophila melanogaster*. *Nat. Methods* **5**, 49–51 (2008).

59. A. C. Martin, M. Kaschube, E. F. Wieschaus, Pulsed contractions of an actin–myosin network drive apical constriction. *Nature* **457**, 495–499 (2009).

60. D. Shcherbo, C. S. Murphy, G. V. Ermakova, E. A. Solovieva, T. V. Chepurnykh, A. S. Shcheglov, V. V. Verkhusha, V. Z. Pletnev, K. L. Hazelwood, P. M. Roche, S. Lukyanov, A. G. Zaraisky, M. W. Davidson, D. M. Chudakov, Far-red fluorescent tags for protein imaging in living tissues. *Biochem. J.* **418**, 567–574 (2009).

61. D. Kong, Z. Lv, M. Häring, B. Lin, F. Wolf, J. Großhans, *In vivo* optochemical control of cell contractility at single-cell resolution. *EMBO Rep.* **20**, e47755 (2019).

62. D. S. Bindels, L. Haarbosch, L. van Weeren, M. Postma, K. E. Wiese, M. Mastop, S. Aumonier, G. Gotthard, A. Royant, M. A. Hink, T. W. J. Gadella, mScarlet: A bright monomeric red fluorescent protein for cellular imaging. *Nat. Methods* **14**, 53–56 (2017).

63. M. González, I. Martín-Ruíz, S. Jiménez, L. Pirone, R. Barrio, J. D. Sutherland, Generation of stable *Drosophila* cell lines using multicistronic vectors. *Sci. Rep.* **1**, 75 (2011).

64. A. Defaye, L. Perrin, Tissue specific RNA isolation in Drosophila embryos: A strategy to analyze context dependent transcriptome landscapes using FACS, in *Hox Genes: Methods and Protocols,* Y. Graba, R. Rezsohazy, Eds. (Springer New York, 2014), pp. 183–195.

65. W. Thielicke, R. Sonntag, Particle image velocimetry for MATLAB: Accuracy and enhanced algorithms in PIVlab. *J. Open Res. Softw.* **9** (2021).

66. W. Thielicke, E. J. Stamhuis, PIVlab—Towards user-friendly, affordable and accurate digital particle image velocimetry in MATLAB. *J. Open Res. Softw.* **2**, e30 (2014).

67. P. Coupé, M. Munz, J. V. Manjón, E. S. Ruthazer, D. Louis Collins, A CANDLE for a deeper in vivo insight. *Med. Image Anal.* **16**, 849–864 (2012).

68. T. W. Ridler, S. Calvard, Picture thresholding using an iterative selection method. *IEEE Trans. Syst. Man Cybern.* **8**, 630–632 (1978).

69. G. K. Agrawal, J. J. Thelen, A modified Pro-Q diamond staining protocol for phosphoprotein detection in polyacrylamide gels, in *The Protein Protocols Handbook,* J. M. Walker, Ed. (Humana Press, 2009), pp. 579–585.

70. D. Pinheiro, E. Hannezo, S. Herszterg, F. Bosveld, I. Gaugue, M. Balakireva, Z. Wang, I. Cristo, S. U. Rigaud, O. Markova, Y. Bellaïche, Transmission of cytokinesis forces via E-cadherin dilution and actomyosin flows. *Nature* **545**, 103–107 (2017).

71. M. Rauzi, P.-F. Lenne, T. Lecuit, Planar polarized actomyosin contractile flows control epithelial junction remodelling. *Nature* **468**, 1110–1114 (2010).
